# Leuco Ethyl Violet as Self‐Activating Prodrug Photocatalyst for In Vivo Amyloid‐Selective Oxygenation

**DOI:** 10.1002/advs.202401346

**Published:** 2024-04-30

**Authors:** Masahiro Furuta, Suguru Arii, Hiroki Umeda, Ryota Matsukawa, Katsuyuki Shizu, Hironori Kaji, Shigehiro A. Kawashima, Yukiko Hori, Taisuke Tomita, Youhei Sohma, Harunobu Mitsunuma, Motomu Kanai

**Affiliations:** ^1^ Graduate School of Pharmaceutical Sciences The University of Tokyo 7‐3‐1 Hongo, Bunkyo‐ku Tokyo 113‐0033 Japan; ^2^ Institute for Chemical Research Kyoto University Kyoto 611‐0011 Japan; ^3^ School of Pharmaceutical Sciences Wakayama Medical University Wakayama 640‐8156 Japan; ^4^ PRESTO JST 4‐1‐8 Honcho, Kawaguchi Saitama 332‐0012 Japan

**Keywords:** Alzheimer disease, amyloid, ethyl violet, photooxygenation catalyst, prodrug

## Abstract

Aberrant aggregates of amyloid‐β (Aβ) and tau protein (tau), called amyloid, are related to the etiology of Alzheimer disease (AD). Reducing amyloid levels in AD patients is a potentially effective approach to the treatment of AD. The selective degradation of amyloids via small molecule‐catalyzed photooxygenation in vivo is a leading approach; however, moderate catalyst activity and the side effects of scalp injury are problematic in prior studies using AD model mice. Here, leuco ethyl violet (LEV) is identified as a highly active, amyloid‐selective, and blood‐brain barrier (BBB)‐permeable photooxygenation catalyst that circumvents all of these problems. LEV is a redox‐sensitive, self‐activating prodrug catalyst; self‐oxidation of LEV through a hydrogen atom transfer process under photoirradiation produces catalytically active ethyl violet (EV) in the presence of amyloid. LEV effectively oxygenates human Aβ and tau, suggesting the feasibility for applications in humans. Furthermore, a concept of using a hydrogen atom as a caging group of a reactive catalyst functional in vivo is postulated. The minimal size of the hydrogen caging group is especially useful for catalyst delivery to the brain through BBB.

## Graphical abstract

Leuco ethyl violet (LEV), identified as a highly active, amyloid‐selective, and blood‐brain barrier permeable photooxygenation catalyst, functions as a self‐activating pro‐catalyst. Under photoirradiation, LEV undergoes self‐oxidation through a hydrogen atom transfer process, forming catalytically active ethyl violet (EV) in amyloid's presence. This enables in vivo photooxygenation of amyloid‐β without inducing scalp injury and facilitates the oxygenation of amyloids in Alzheimer disease patients.

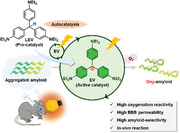

## 1 Introduction

Alzheimer disease (AD) is a chronic, progressive, neuro‐degenerative brain disorder associated with loss of memory and cognitive decline.^[^ ^1^ ^]^ AD is pathologically characterized by two types of lesions: senile plaques and neurofibrillary tangles. These are composed of aberrantly aggregated amyloid‐β (Aβ) and tau, respectively. Protein aggregates like these are called amyloid.^[^ ^2^, ^3^, ^4^, ^5^ ^]^ Decreasing amyloid levels in AD patients is an effective treatment of AD; the Food and Drug Administration (FDA) recently approved two monoclonal antibodies to Aβ, aducanumab^[^ ^6^ ^]^ and lecanemab,^[^ ^7^ ^]^ as anti‐AD drugs. The switch from biologics to small‐molecule drugs acting as functional surrogates of biologics, will be an important next step.

**Figure 1:**
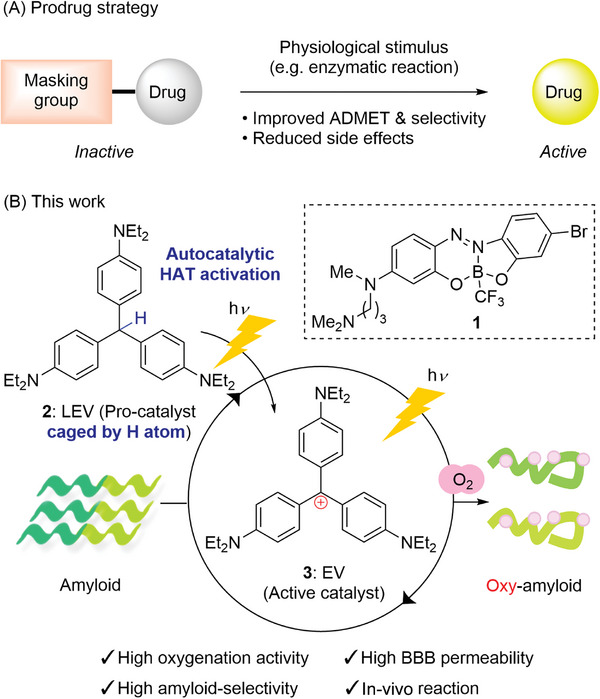
Catalytic photooxygenation of amyloids by prodrug strategy. A) General concept of prodrug strategy. B) Photooxygenation of amyloids by leuco ethyl violet **2** as a procatalyst.

Degradation of amyloids using small‐molecule catalysts is an emerging approach.^[^ ^8^, ^9^, ^10^, ^11^, ^12^, ^13^, ^14^, ^15^, ^16^, ^17^ ^]^ Targeting the cross‐β‐sheet structure characteristic to amyloids, we previously developed amyloid‐selective photooxygenation catalysts.^[^ ^18^, ^19^, ^20^ ^]^ When interacting with the cross‐β‐sheet structure, these catalysts acted as photosensitizers to generate singlet oxygen (^1^O_2_) under light irradiation. Due to the short‐lived nature of ^1^O_2_, it reacted selectively with the proximal amyloid. Specific amino acid residues, such as histidine (His)^[^ ^21^ ^]^ and/or methionine (Met), were oxygenated. The covalent installation of hydrophilic oxygen functionalities into amyloid decreased its aggregative propensity and toxicity. Furthermore, catalyzed photooxygenation of Aβ amyloid facilitated its phagocytotic degradation by microglia cells in the mouse brain.^[^ ^22^ ^]^ Specifically, we achieved non‐invasive photooxygenation and a significant (ca. 30%) decrease of Aβ amyloid in the brains of living AD model mice through intravenous administration of an azobenzene‐boron complex catalyst (ABB: **1**) and light irradiation (*λ* = 595 nm) to the head.^[^ ^20^ ^]^ However, two main challenges remain for catalyst **1**. First, the activity was moderate, and photooxygenation did not proceed when using lysate from an AD patient's brain. Second, the treatment induced scalp injury as a side effect, likely due to the low blood‐brain barrier (BBB) permeability and insufficient target selectivity of **1**. To overcome these challenges, we investigated using a prodrug strategy (**Figure** **1**A)^[^ ^23^ ^]^ with the catalytic treatment, so called catalysis medicine.^[^ ^24^ ^]^ This strategy would maximize the efficacy by improving the ADMET (absorption, distribution, metabolism, excretion, and toxicity) properties of a highly active catalyst while reducing its potential toxicity. We envisioned that an external stimulus (photoirradiation in this study) would generate a highly active photocatalyst from a pro‐catalyst with better ADMET properties in the vicinity of amyloid selectively, thereby enabling efficient photooxygenation with minimal side effects.

In this study, we report that leuco ethyl violet (LEV: **2**) functions as a less toxic and BBB‐permeable pro‐catalyst of ethyl violet (EV: **3**), whose photooxygenation activity is two orders of magnitude greater than that of ABB **1** (Figure 1B). Photoirradiation to **2** locally generates catalytically active **3** near amyloid through an autocatalytic hydrogen atom transfer (HAT) mechanism, enabling selective photooxygenation of Aβ amyloid. The high BBB permeability and amyloid selectivity of **2** have allowed us to achieve in vivo photooxygenation of Aβ without scalp injury. Furthermore, **2** can oxygenate AD patient‐derived human Aβ and tau amyloids, suggesting its potential as a multi‐targeting agent for the treatment of AD.

## 2 Results and Discussion

We envisioned trityl cations as a new photocatalyst scaffold with greater activity than **1**. The heteroatom‐substituted triarylmethane dyes (hsTMDs) are stable trityl cations of wide utility, being used as fluorescent probes,^[^ ^25^, ^26^, ^27^ ^]^ pH indicators,^[^ ^28^, ^29^ ^]^ photocatalysts,^[^ ^30^, ^31^ ^]^ and drugs.^[^ ^32^ ^]^ In the photoexcited state, hsTMDs furnish n–π* electron transition, which facilitates the intersystem crossing (ISC) process^[^ ^33^ ^]^ and enhances the photooxygenation activity. In addition, their molecular size is generally small, while still absorbing relatively long‐wavelength light. This property is advantageous to BBB permeability^[^ ^34^ ^]^ and in vivo applications. Furthermore, hsTMDs bear an aggregation‐induced emission (AIE) switch^[^ ^35^, ^36^ ^]^ to turn on/off depending on the environment (i.e., binding/non‐binding to amyloids), like Thioflavin T (ThT) dyes do.^[^ ^19^, ^37^ ^]^ Last but not least, TMDs were reported to bind to Aβ and other amyloids containing cross‐β‐sheet structures and reduce their toxicity and aggregation propensity by acting as aggregation inhibitors.^[^ ^38^, ^39^, ^40^, ^41^, ^42^, ^43^ ^]^

**Figure 2:**
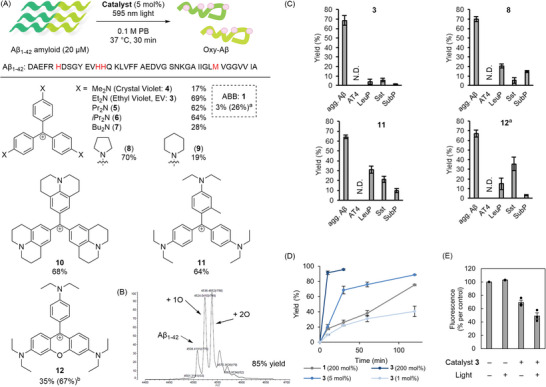
Photooxygenation of aggregated Aβ using triarylmethane catalysts. A) Activity‐based catalyst screening. A 0.1 m phosphate buffer (PB) solution (pH 7.4) containing aggregated Aβ_1–42_ (20 × 10^−6^ m) and catalyst (1 × 10^−6^ m, 5 mol%) was photoirradiated (*λ* = 595 nm, 10 mW) at 37 °C for 30 min. Oxygenation yield is the sum of the yields of products that are oxygenated in at least one sites. Yield was determined by MALDI‐TOF MS analysis (average of *n* = 3 experiments). ^a^200 mol% instead of 5 mol% catalyst was used. ^b^500 nm light instead of 595 nm light was used. B) A representative MALDI‐TOF MS chart for photooxygenation of Aβ using **3** (EV). C) Evaluation of amyloid selectivity. A PB solution (pH 7.4) containing aggregated Aβ_1–42_ (agg. Aβ), angiotensine‐IV (AT4: amino acid sequence with underlined potential oxidation sites: VYIHPF), leuprorelin (LeuP: PyroEHWSYLLRP), somatostatin (Sst: AGCKNFFWKTFTSC), or [Tyr^8^]‐substance P (SubP: RPKPQQFYGLM‐NH_2_) (20 × 10^−6^ m each) was photoirradiated (*λ* = 595 nm, 10 mW) in the presence of catalyst **3** (EV), **8**, **11**, or **12** (each 1 × 10^−6^ m, 5 mol%) at 37 °C for 30 min. Yield was analyzed using MALDI‐TOF MS (*n* = 3 experiments, mean ± SEM). ^a^500 nm light instead of 595 nm light was used. D) Reaction profile. A PB solution (pH 7.4) containing Aβ_1–42_ (20 × 10^−6^ m) and **3** (0.2 × 10^−6^, 1 × 10^−6^, or 40 × 10^−6^ m) or ABB (40 × 10^−6^ m) was photoirradiated (*λ* = 595 nm, 10 mW) at 37 °C for certain time periods. Yield was analyzed using MALDI‐TOF MS (*n* = 3 experiments, mean ± SEM). E) Evaluation of amyloidogenic cross‐β‐sheet propensity by Thioflavin T (ThT) fluorescence assay with or without treatment by catalyst **3** (EV). Lane 4 (catalyst + and light +): A 0.1 m PB solution (pH 7.4) containing monomer Aβ_1–42_ (20 × 10^−6^ m) and catalyst **3** (1 × 10^−6^ m, 5 mol%) was photoirradiated (*λ* = 595 nm, 10 mW) at 37 °C for 3 h. After the reaction, the aggregation level was evaluated with ThT fluorescence (*n* = 3 experiments, mean ± SEM).

We screened hsTMDs in photooxygenation of aggregated Aβ under 595 nm light irradiation to identify ethyl violet (EV: **3**) as the most active and selective catalyst (69% yield, **Figures** **2**A–C and S1, Supporting Information). Oxygenation yield was calculated from MALDI‐TOF MS analysis (Figure 2B). No heavy atom, such as a halogen atom, was required for the photooxygenation activity. The catalyst activity was dependent on the substituents on the nitrogen atoms (**3**‒**10**). The balance between water solubility and Aβ binding affinity might be attributable to this tendency. Catalyst **11**, bearing an *ortho*‐methyl group at the trityl core skeleton, and **12**, with a rhodamine skeleton, also showed high activity.

To evaluate Aβ amyloid selectivity, we compared photooxygenation yield of Aβ with four non‐aggregative peptides (angiotensin IV (AT4), somatostatin (Sst), leuprorelin (LeuP), and [Tyr^8^]‐substance P (SubP)) as off‐target models, using catalysts with high activity (**3**, **8**, **11**, or **12**) (Figure 2C). Yield for the photooxygenation of the non‐aggregative peptides was less than 6% using **3**, while it was 13–35% using **8**, **11**, or **12**. The high off‐target oxygenation level using **8** was likely due to the self‐aggregation of the catalyst in an aqueous solvent; the absorption spectrum of **8** showed a larger peak ratio at 600 nm/ca. 550 nm than **3** did, suggesting formation of self‐aggregates^[^ ^44^ ^]^ (Figures S2 and S3, Supporting Information). Self‐aggregation would turn on the AIE switch of **8** to generate ^1^O_2_ irrespective of the existence of amyloid or promote excimer formation and subsequent single electron reduction of molecular oxygen through a type I mechanism to generate highly oxidative superoxide anion radical (O_2_^•–^).^[^ ^45^ ^]^ Compared to **3**, the cyclic alkyl amine substituents of **8** diminished the molecular flexibility and steric hindrance, promoting self‐aggregation. For **11** and **12**, the single bond rotation between the aryl group and the cationic carbon atom was partly inhibited. This accelerated non‐selective oxygenation of off‐target peptides. We also confirmed the high amyloid selectivity of **3** by using lysozyme as an off‐target non‐aggregative protein model (Figure S4, Supporting Information).

Next, we characterized **3** regarding the reaction kinetics, optical properties, and structures/aggregation propensities of the products. The catalyst activity of **3** was ca. >100 times greater than **1** (Figure 2D). The initial kinetics using 1 mol% **3** were almost comparable to 200 mol% **1**, whereas the reaction did not reach completion. This was likely due to catalyst deactivation such as photodegradation during the reaction. However, because photooxygenated Aβ inhibited aggregation of native Aβ and thus markedly decreased the toxicity of Aβ amyloid despite the limited oxygenation (ca. 15% yield),^[^ ^18^, ^19^ ^]^ **3** can still be effective under low catalyst concentrations, especially in in vivo applications (see Figure 6A and Figure S29, Supporting Information). The maximum absorption wavelength of **3** redshifted from 595 to 603 nm in the presence of Aβ amyloid (Figure S3, Supporting Information). Furthermore, the fluorescence intensity was increased by 19‐fold in the presence of Aβ amyloid (Figure S5, Supporting Information). These optical properties indicate that **3** binds with Aβ amyloid, thereby inhibiting the relaxation process and promoting fluorescence emission. Based on the MALDI‐TOF MS analysis of the Aβ photooxygenation products after enzymatic digestion, the oxygenated amino acid residues were His^6^, His^13^, His^14^, and Met^35^ (Figure 2A and Figure S6, Supporting Information). It was reported that His oxygenation produced crosslinked products through the intermolecular addition reaction of nucleophilic amino acid residues to the electrophilic oxygenated His intermediates (Figure S7, Supporting Information).^[^ ^21^, ^46^ ^]^ Thus, a crosslinked fragment of FRHD at the oxygenated His residue was also detected (Figure S6, Supporting Information). The ThT fluorescence intensity, an indicator of the cross‐β‐sheet propensity,^[^ ^37^ ^]^ was significantly lower for the photooxygenated products than for the control samples without photooxygenation (Figure 2E). ThT fluorescence also decreased for the catalyst‐only sample. This result suggests that **3** shares the same binding site as ThT on Aβ amyloid.

**Figure 3:**
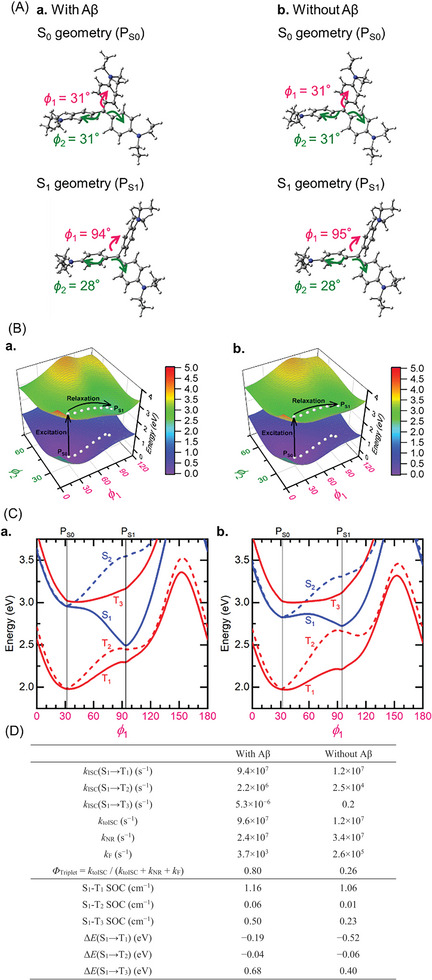
DFT calculation of EV (**3**). A) Optimized S_0_ and S_1_ geometries calculated using the M06‐2X/6‐31G(d) and TDA‐M06‐2X/6‐31G(d) methods, respectively. (a) **3** with binding Aβ (*ε*_r_ = 1); (b) **3** without binding Aβ (*ε*_r_ = 78). B) Potential energy surfaces for the ground state (S_0_) and lowest excited singlet states (S_1_). (a) **3** with binding Aβ (*ε*_r_ = 1); (b) **3** without binding Aβ (ε_r_ = 78). White dots depict geometry relaxation paths. C) Potential energy curves for the excited singlet states (S_1_ and S_2_) and excited triplet states (T_1_, T_2_, and T_3_) calculated along the decay paths in Figure 3B. (a) **3** with binding Aβ; (b) **3** without binding Aβ. D) Rate constants for ISC (*k*_ISC_), non‐radiative decay (*k*_NR_), and fluorescence (*k*_F_), spin–orbit couplings (SOCs), and excited‐state energy differences (Δ*E*), calculated at the TDA‐M06‐2X/6‐31G(d) level of theory.

We conducted density functional theory (DFT) calculations to understand why **3** shows a higher triplet generation ability when binding Aβ than when not (Tables S1‒S4, Supporting Information). First, we optimized the ground‐state (S_0_) geometries of **3** using the M06‐2X/6‐31G(d) method. Second, we optimized the lowest excited singlet state (S_1_) geometries using the M06‐2X/6‐31G(d) method under Tamm‐Dancoff approximation (TDA). To model **3** without binding Aβ in an aqueous solution, we employed the polarized continuum model and the relative permittivity of water (*ε*_r_ = 78). Meanwhile, we set *ε*_r_ = 1 to model **3** binding with Aβ, based on the reported docking structure between **4** and an Aβ trimer model (Figure S8, Supporting Information).^[^ ^43^ ^]^ The optimized S_0_ geometries (denoted P_S0_) with and without binding Aβ belong to the *D*_3h_ symmetry, whereas the optimized S_1_ geometries (denoted P_S1_) belong to the *C*_2_ symmetry (**Figure** **3**A). Comparing the optimized S_0_ and S_1_ geometries shows that the torsion angle of one of the benzene rings (*ϕ*_1_) increases from 31° to 94°/95° during the geometry relaxation after S_0_‐S_1_ excitation. Meanwhile, the torsion angles of the other benzene rings (*ϕ*_2_) essentially do not change (from 31° to 28°). Then, we calculated the S_0_ and S_1_ potential energy surfaces for *ϕ*_1_ and *ϕ*_2_ rotations with and without binding Aβ (Figure 3B). White dots on the green surface in Figure 3B show possible decay paths from the S_0_ to S_1_ geometries in the S_1_ state. The *ϕ*_1_ rotation is relevant for the geometry deformation. Finally, we calculated potential energy curves for S_0_, excited singlet states (S_1_ and S_2_), and excited triplet states (T_1_, T_2_, and T_3_) along the decay paths (as a function of *ϕ*_1_) (Figure 3C). There is no crossing point between the potential energy curves of the excited singlet and triplet states, indicating that the transition from the excited state to the ground state proceeds after relaxation to the most stable geometry in the S_1_ state. All the calculations were performed with the Gaussian 16 program package.^[^ ^47^ ^]^

We calculated the rate constants for S_1_→T_1_ ISC (*k*_ISC_(S_1_‐T_1_)), S_1_→T_2_ ISC (*k*_ISC_(S_1_‐T_2_)), S_1_→T_3_ ISC (*k*_ISC_(S_1_‐T_3_)), S_1_→S_0_ fluorescence (*k*_F_), and S_1_→S_0_ nonradiative decay (*k*_NR_) at the optimized S_1_ geometries (Figure 3D).^[^ ^48^ ^]^ We estimated the rate constant of total ISC (*k*_to_ _ISC_) and triplet generation quantum yield (ϕ_Triplet_) in **3** as:

(1) $$k_{\mathrm{toISC}}=k_{\mathrm{ISC}}\left(S_{1}\to T_{1}\right)+k_{\mathrm{ISC}}\left(S_{1}\to T_{2}\right)+k_{ISC}\left(S_{1}\to T_{3}\right)$$

(2) $$\phi_{\mathrm{Triplet}}=k_{\mathrm{toISC}}/\left(k_{\mathrm{toISC}}+k_{F}+k_{\mathrm{NR}}\right)$$

The calculated *ϕ*_Triplet_ is larger for **3** with binding Aβ (0.80) than without binding Aβ (0.26). This is primarily because the *k*_toISC_ value is more considerable with binding Aβ (9.6 × 10^7^ s^−1^) than without binding Aβ (1.2 × 10^7^ s^−1^). The *k*_F_ values are far smaller than the *k*_toISC_ values with and without Aβ and do not substantially change *ϕ*_Triplet_. From Figure 3D, the *k*_toISC_ values are almost identical to the *k*_ISC_(S_1_→T_1_) values, suggesting that the S_1_→T_1_ ISC dominates the total ISC process. Thus, the difference in *ϕ*_Triplet_ is attributed to the difference in *k*_ISC_(S_1_→T_1_). *k*_ISC_(S_1_→T_1_) increases by decreasing the S_1_–T_1_ energy difference (Δ*E*) and increasing the S_1_–T_1_ spin–orbit coupling (SOC). The theoretical calculations show that the S_1_–T_1_ SOCs are of the same order of magnitude regardless of with or without binding Aβ (1.16 and 1.06 cm^−1^, respectively), suggesting that the smaller ǀΔ*E*(S_1_–T_1_)ǀ with binding Aβ (0.19 eV; 0.52 eV without Aβ) is responsible for the larger *k*_ISC_(S_1_→T_1_). The energy transfer from the excited catalyst in the T_1_ state to molecular oxygen (^3^O_2_) affords ^1^O_2_, which reacts with nearby amyloid (Figure S9, Supporting Information). Thus, the activity turn‐on mechanism of **3** by binding amyloid is due to the enhanced ISC kinetics from S_1_ to T_1_ by sensing the hydrophobic environment (i.e., small ε*_r_*) of amyloid.

Toward in vivo applications, we evaluated cytotoxicity of catalyst **3** to find that **3** was highly toxic with the LD_50_ value for PC12 cells to be 0.14 × 10^−6^ m under dark conditions (Figure S10, Supporting Information). The high toxicity of **3** was likely due to its cationic character and interaction with cell membranes.^[^ ^49^ ^]^ We hypothesized that the reduced form **2** would act as a precursor of **3** through autocatalytic oxidation.^[^ ^50^ ^]^ This oxidative procatalyst activation would be accelerated in the presence of amyloids, further enhancing the amyloid selectivity. Based on this hypothesis, we synthesized leuco ethyl violet (LEV: **2**). As expected, toxicity of **2** was markedly decreased with its LD_50_ >10 × 10^−6^ m (Figure S10, Supporting Information). Furthermore, the BBB permeability of **2** was improved compared to **1** and **3**; the recovery rates of **2**, **1**, and **3** from mice brains at 10 min after intravenous injection were 1.7%, 0.58%, and 0.046%, respectively (Figure S11, Supporting Information). The maximum absorption wavelength of **2** blue‐shifted from 288 nm to 283 nm in the presence of Aβ amyloid (Figure S12, Supporting Information), suggesting that **2** interacts with Aβ amyloid.

**Figure 4:**
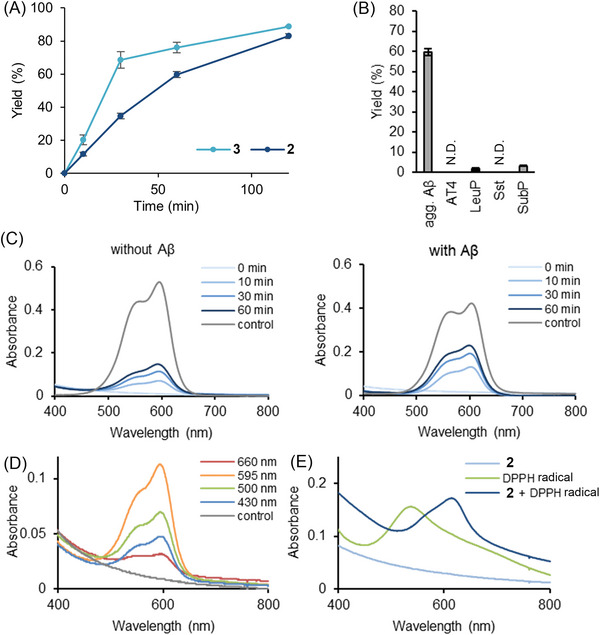
Photooxygenation of Aβ amyloid using LEV (**2**). A) Reaction time course. A PB solution (pH 7.4) containing aggregated Aβ_1–42_ (20 × 10^−6^ m) and **2** or **3** (1 × 10^−6^ m, 5 mol%) was photoirradiated (*λ* = 595 nm, 10 mW) at 37 °C, and the reaction progress was analyzed using MALDI‐TOF MS (*n* = 3 experiments, mean ± SEM). B) Evaluation of amyloid selectivity. A PB solution (pH 7.4) containing aggregated Aβ_1–42_ (agg. Aβ), AT4, LeuP, Sst, or SubP (20 × 10^−6^ m each) was photoirradiated (*λ* = 595 nm, 10 mW) in the presence of **2** (1 × 10^−6^ m, 5 mol%) at 37 °C for 60 min. Yield was analyzed using MALDI‐TOF MS (*n* = 3 experiments, mean ± SEM). C) Spectroscopic time course for the generation of **3** from **2**. A PB solution (pH 7.4) containing **2** (20 × 10^−6^ m) with (right) or without (left) Aβ amyloid (20 × 10^−6^ m) was photoirradiated (*λ* = 595 nm, 10 mW) at 37 °C, and the absorption spectra were measured. The control is the absorption spectrum of **3**. D) Conversion of **2** to **3** under photoirradiation with variable wavelength light. A PB solution (pH 7.4) containing **2** (20 × 10^−6^ m) was irradiated at the indicated wavelength (10 mW) at 37 °C for 30 min, and the absorption spectra were measured. The control is the absorption spectrum of **2**. E) Activation of **2** to **3** through a hydrogen atom transfer (HAT) process. To a PB solution (pH 7.4) of **2** (20 × 10^−6^ m), 1,1‐diphenyl‐2‐picrylhydrazyl (DPPH) radical (20 × 10^−6^ m) was added and the mixture was incubated in the dark at 37 °C for 30 min under air. After incubation, the absorption spectrum of the solution was measured.

We then studied the photooxygenation ability and selectivity of **2** to Aβ amyloid (**Figure** **4**). The reaction proceeded in high yield over 2 h using 5 mol% **2** (Figure 4A and Figure S13, Supporting Information). LEV **2** showed slower kinetics at the initial stage (ca. 1 h) than EV **3**, but both catalysts reached comparable yield after 2 h. Photooxygenated Aβ by **2** exhibited decreased cross‐β‐sheet propensity based on the ThT fluorescence detection (Figure S14, Supporting Information), as was observed using **3**. The Aβ amyloid selectivity of **2** to off‐target model peptides and a protein (lysozyme) was even higher than that of **3** (Figure 4B and Figures S3 and S15, Supporting Information).

We reasoned that the active catalyst when using **2** is indeed **3**, on the basis of the following results. First, we observed the formation of **3** from **2** by photoirradiation at *λ* = 595 nm either in the absence or presence of Aβ amyloid. The absorption peak at 600 nm, characteristic to **3**, increased according to the time course (Figure 4C). The conversion rate was greater in the presence of Aβ than in its absence (54% vs 28% at 60 min). The light‐induced conversion from **2** to **3** was also confirmed by LC‐MS analysis and fluorescence spectroscopy (Figure S16, Supporting Information). This conversion did not proceed without light irradiation. However, the light absorbance of **2** at *λ* = 595 nm was very weak (Figure S12, Supporting Information). To gain deeper insight into what species absorbs light for the conversion from **2** to **3**, we studied this process by irradiating **2** with variable wave‐length light. The formation of **3** was enhanced in the order of *λ* = 595, 500, 430, and 660 nm (Figure 4D). This tendency is consistent with the absorption coefficient of **3**. Therefore, **3** must be responsible for the activation of **2** to **3** and thus this process is autocatalytic.^[^ ^51^ ^]^

Then, we collected mechanistic information for the conversion from **2** to **3**. The formation of **3** was retarded under degassed conditions, indicating the involvement of ^3^O_2_ (Figure S17, Supporting Information). However, ^1^O_2_ was not likely relevant to this process. Thus, the addition of NaN_3_, a ^1^O_2_ scavenger,^[^ ^51^ ^]^ did not affect the light‐induced generation of **3** from **2** (Figure S18, Supporting Information). Furthermore, treatment of **2** with ^1^O_2_ generated from H_2_O_2_ and Na_2_MoO_4_ without photoirradiation,^[^ ^52^, ^53^ ^]^ did not produce **3** (Figure S19, Supporting Information). The addition of 1,1‐diphenyl‐2‐picrylhydrazyl (DPPH) radical^[^ ^54^ ^]^ acting as a hydrogen atom abstracting reagent from the central methine C─H bond of **2**, however, led to the formation of **3** under aerobic conditions without photoirradiation (Figure 4E). These results all support that photoexcited **3** works as a hydrogen atom transfer (HAT) catalyst in the activation of **2** to **3**.^[^ ^55^ ^]^

**Figure 5:**
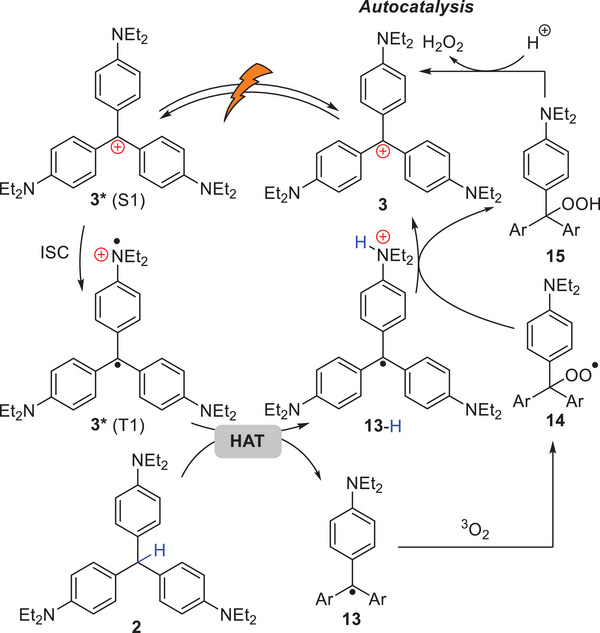
Proposed mechanism for conversion of **2** to **3**.

Based on the above results, we proposed a plausible mechanism for the activation of **2** to **3** (**Figure** **5**). A small amount of **3** is generated by autooxidation of **2**. Photoexcitation of **3**, followed by ISC affords excited **3** in a triplet state [3* (T_1_)], which abstracts the methine hydrogen atom of **2** to generate trityl radicals **13** and **13‐H**. DFT calculation suggested that this HAT process is thermodynamically feasible (Figure S20, Supporting Information). **13** reacts with ^3^O_2_ to produce peroxy radical **14**. Subsequently, the reaction between **13‐H** and **14** proceeds through another HAT process, or a stepwise single electron transfer (SET) from **13‐H** to **14** followed by proton transfer, to regenerate **3** and hydroperoxide **15**. Finally, elimination of hydrogen peroxide from **15** affords **3**. The generation of H_2_O_2_ in photoirradiation of **2** to form **3** was confirmed by an iodometry experiment (Figure S21, Supporting Information also see Figures S22–S25, Supporting Information, for further mechanistic supports). Furthermore, since the photocatalytic activity of **3** is turned on by binding to Aβ amyloid, the activation from **2** to **3** is also accelerated in the presence of amyloid. This enhances amyloid selectivity of **2** compared to **3**. Autocatalytically activated **3** initiates the facilitated generation of ^1^O_2_ under photoirradiation in the hydrophobic environment proximate to amyloid, leading to selective oxygenation of the amyloid.

**Figure 6:**
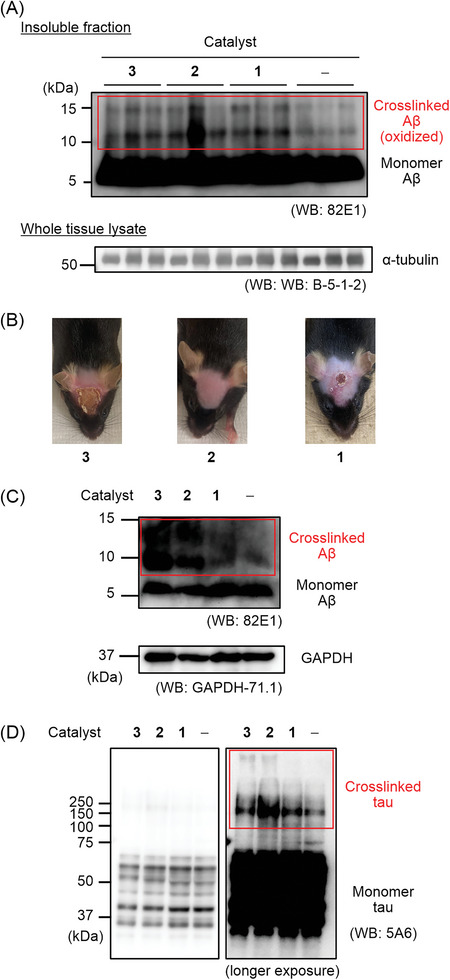
In vivo and ex‐vivo photooxygenation reaction. A) In vivo photooxygenation reaction. A solution of EV **3**, LEV **2**, or ABB **1** was intravenously injected into 5–6 month old *App^NL‐G‐F/NL‐G‐F^* mice (*n* = 3 experiments, each group) expressing human Arctic Aβ. After an interval, the mice were irradiated with LED (*λ* = 595 nm) for 10 min. The operation set (catalyst injection and photoirradiation) was repeated five times over 5 d. At 24 h after the final operation set, the brain was excised and homogenized using a 1× PBS buffer. After the fractionation, the insoluble fraction was analyzed by SDS‐PAGE using a 15% Tris‐Tricine gel and Western blot (WB) using an anti‐Aβ antibody. For loading controls, α‐tubulin in lysates before fractionation was analyzed. B) Photos of mouse scalp after the photooxygenation treatment in (A). C, D) Catalytic photooxygenation of human brain lysate. The temporal cortex of an AD patient was homogenized using a 10× volume of PBS (containing cOmplete EDTA+ (Roche) and PhosSTOP (Sigma)). A catalyst (2.5 × 10^−6^ m) was added to the brain lysate and the mixture was irradiated with 595 nm light for 3 h or kept in the dark at 37 °C. The resulting mixture was analyzed with SDS‐PAGE and WB (anti‐Aβ antibodies: 82E1 (IBL) and anti‐GAPDH antibodies: GAPDH‐71.1 for (C), anti‐tau antibodies: 5A6 for (D)). Human AD‐tau is comprised of 6 isoforms, whose sizes are 36.8–45.9 kDa. CBB staining was shown in Figure S30 (Supporting Information).

We confirmed the greater catalytic activity of **2** and **3** compared to **1** using brain lysates derived from AD model mice (*App^NL‐G‐F/NL‐G‐F^*)^[^ ^56^ ^]^ expressing Aβ_1–38_ and Aβ_1–42_ containing the human Arctic mutation (Figures S26‒S28, Supporting Information).^[^ ^57^ ^]^ Then, in vivo photooxygenation of Aβ amyloid in *App^NL‐G‐F/NL‐G‐F^* mice brains was examined. To a 6 month old *App^NL‐G‐F/NL‐G‐F^* mouse containing matured Aβ amyloid,^[^ ^58^ ^]^ a catalyst was intravenously injected, and the head of the mouse was irradiated with 595 nm LED light for 10 min. This treatment was repeated 5 times over 5 days. After the treatment, the brain was extracted and the progress of photooxygenation of Aβ was evaluated by Western blot (WB) analysis. When treated with catalyst **1**, **2**, or **3**, Aβ dimer and trimer bands at ca. 10 and 15 kDa, respectively, increased compared to the control samples without the catalyst treatment (**Figure** **6**A).^[^ ^21^ ^]^ This result indicates that the catalytic photooxygenation of Aβ indeed proceeded in vivo. Furthermore, while **3** and **1** damaged the mice scalp tissue, the treatment with **2** produced few if any apparent side effects (Figure 6B). This stark difference is likely due to the higher BBB permeability and amyloid selectivity of **2** relative to **1** and **3**.

A preventive approach inhibiting amyloid formation through intervention at an early stage of the disease before symptoms appear is also important for AD treatment. To mimic such preventive treatment, we investigated a chronic administration (2 months) of catalyst **2** to 2–3 month old *App^NL‐G‐F/NL‐G‐F^* mice containing less matured Aβ amyloid. The dimer and trimer Aβ bands again increased in the mice brains treated with **2** (Figure S29, Supporting Information). This result suggests the applicability of **2** towards the prevention of AD.

Finally, we investigated the catalytic photooxygenation of Aβ in human AD‐brain lysate. It has been argued that human Aβ fibrils expressed in AD model mice brains do not reproduce the higher‐order structures of Aβ fibrils in human brains of sporadic AD.^[^ ^59^ ^]^ Therefore, the applicability of the catalysts to Aβ amyloid in human AD‐brain lysate is critically important. Intensities of the WB bands corresponding to Aβ dimer and trimer were significantly greater for the samples treated with **3** or **2** than the samples treated with **1** or the negative control without treatment (Figure 6C and Figure S30, Supporting Information). Furthermore, we analyzed the effect of these catalysts on tau in the same samples, as aggregated tau is also photooxygenated by another catalyst.^[^ ^60^ ^]^ The reaction with an AD patient‐derived tau is challenging due to its large molecular size and the presence of many isoforms and post‐translational modifications. We found that crosslink products of tau also increased following catalytic photooxygenation, especially when using **2** (Figure 6D). These results suggest that **2** can be a multi‐targeting catalyst against Aβ and tau, both of which are related to AD etiology.^[^ ^61^ ^]^

## 3 Conclusion

Catalytic photooxygenation of amyloid is an emerging approach to the development of therapies treating AD, a cognitive disease currently difficult to cure. Considering the limited light intensity available in the deep brain by non‐invasive light irradiation from outside the body, however, increasing the catalyst activity is critically important from a chemical perspective. In this study, we identified EV **3** as a highly active and amyloid‐selective photooxygenation catalyst. The activity of **3** was two orders of magnitude greater than that of the previous catalyst **1**,^[^ ^20^ ^]^ which was applicable to non‐invasive in vivo photooxygenation of Aβ amyloid in AD model mice brains. Theoretical calculations rationalized the high activity and selectivity of **3** by the facilitated ISC under a hydrophobic environment when binding to Aβ amyloid. However, **3** was cytotoxic due to its cationic characteristics. Therefore, we developed a procatalyst, LEV **2**, which is charge neutral. Photoirradiation in the presence of amyloid activated **2** to generate **3** through an autocatalytic HAT mechanism. Catalyst **2** was less toxic and furnished favorable properties for in vivo applications; high activity and amyloid selectivity, enhanced BBB permeability, and absorption of tissue‐permeable long‐wavelength light. We established the superiority of **2** over **1** and **3** by demonstrating its reduced side effects and applicability to Aβ and tau amyloids derived from a human AD patient.

This is the first demonstration of autocatalytic oxidative activation of a leuco dye used as a caged prodrug by spatiotemporally controllable external stimuli, such as light, without the exogenous addition of other chemical species. This approach has improved ADMET and enhanced amyloid selectivity, while generating a highly active photo‐catalyst on‐demand. A room for improvement is the light wavelength required for the excitation of active catalyst **2** (595 nm). For applications to higher animal models having greater brain size, catalysts activatable by near infrared light is preferable.^[^ ^62^ ^]^ Further optimization of the catalyst structure, as well as the development of a photodevice to transfer light energy deep into the body, are currently ongoing.

## Conflict of Interest

The authors declare no conflict of interest.

## Supporting information

Supporting Information

## Data Availability

The data that support the findings of this study are available in the Supporting Information of this article.
