# Supplementary material for: Leuco Ethyl Violet as Self‐Activating Prodrug Photocatalyst for In Vivo Amyloid‐Selective Oxygenation
Source: Adv Sci (Weinh). 2024 Apr 30;11(26):2401346. doi: 10.1002/advs.202401346 (PMC11234409; doi:10.1002/advs.202401346)
Supplement: Supplementary file 1 — Supporting Information [file ADVS-11-2401346-s001.pdf]

ADVANCED SCIENCE
Open Access

# Supporting Information

for *Adv. Sci.*, DOI 10.1002/advs.202401346

# Leuco Ethyl Violet as Self-Activating Prodrug Photocatalyst for In Vivo Amyloid-Selective Oxygenation

*Masahiro Furuta, Suguru Arii, Hiroki Umeda, Ryota Matsukawa, Katsuyuki Shizu, Hironori Kaji, Shigehiro A. Kawashima, Yukiko Hori, Taisuke Tomita, Youhei Sohma, Harunobu Mitsunuma and Motomu Kanai**

**Supporting Information**

# Leuco Ethyl Violet as Self-Activating Prodrug Photocatalyst for In Vivo Amyloid-Selective Oxygenation

Masahiro Furuta,[1] Suguru Arii,[1] Hiroki Umeda,[1] Ryota Matsukawa,[1] Katsuyuki Shizu,[2] Hironori Kaji,[2] Shigehiro A. Kawashima,[1] Yukiko Hori,[1] Taisuke Tomita,[1] Youhei Sohma,[3] Harunobu Mitsunuma,[1,4] and Motomu Kanai[1,]*

[1]Graduate School of Pharmaceutical Sciences, The University of Tokyo, 7-3-1 Hongo, Bunkyo-ku, Tokyo 113-0033, Japan

[2]Institute for Chemical Research, Kyoto University, Gokasho, Uji, Kyoto, 611-0011 Japan

[3]Department of Medicinal Chemistry, School of Pharmaceutical Sciences, Wakayama Medical University, 25-1 Shichiban-cho, Wakayama 640-8156, Japan

[4]Presto, Japan Science and Technology Agency, 4-1-8 Honcho, Kawaguchi, Saitama 332-0012, Japan

*Correspondence: kanai@mol.f.u-tokyo.ac.jp

**Table of Contents**

General
Materials
Methods
Supplemental Figures (S1 to S32) and Tables (S1 to S4)
NMR charts

## General

NMR spectra were recorded on JEOL ECX500 spectrometer, operating at 500 MHz for $^{1}$H NMR and 124.51 MHz for $^{13}$C NMR, or JEOL ECS400 spectrometer, operating at 400 MHz for $^{1}$H NMR, 100 MHz for $^{13}$C NMR, and 369 MHz for $^{19}$F NMR. Chemical shifts were reported in ppm on the δ scale relative to residual $CHCl_3$ (δ = 7.24) for $^{1}$H NMR or to $CDCl_3$ (δ = 77.0) for $^{13}$C NMR as an internal reference, and hexafluorobenzene (δ = –164.90 ppm) for $^{19}$F NMR as an external reference. Analytical HPLC was conducted by using a JASCO HPLC system equipped with a UV-2075 spectrometer, PU-2080 pumps, a DG-2080-54 degasser, and an MX-2080-32 mixer. Preparative HPLC was conducted by using a JASCO HPLC system equipped with a UV-2075 spectrometer, PU-2086 pumps, a DG-2080-53 degasser, and an MX-2080-32 mixer. ESI-MS spectra were measured on Shimadzu LCMS-2020, Agilent Technologies 6120 (for LRMS) equipped with Prominence LC-2030, or a Bruker micrOTOF-II spectrometer (for HRMS). MALDI-TOF MS was obtained with a Shimadzu Biotech Axima ToF$^{2}$ spectrometer and Shimazu MALDI-8030 using α-cyano-4-hydroxy cinnamic acid as a matrix. We acquired and averaged many (200) single-shot spectra from several positions within an identical sample spot to obtain representative sample data. QCM (27 MHz) analysis was performed using an ULVAC AFFINIX QNμ with a gold-electrode sensor cell. Photoreaction was performed with a Valore INSIGHT VAL-S light emitting diode (λ = 430, 500, and 595 nm) and U-Technology UDP2430W-2 and UDB-220R100 (λ = 660 nm). Absorbance measurement was performed using a Shimadzu UV-1800 spectrometer with a rectangular quartz cell (5 mm path length). The fluorescence intensity and spectra were measured on a spectrofluorophotometer RF-5300 (Shimadzu Co.) using a rectangular quartz cell (3 mm path length). Circular dichroism (CD) spectra were recorded with a JASCO J-820 spectrometer with a 1 mm pathlength quartz cell with a scanning speed of 200 nm $min^{-1}$. Theoretical calculations were conducted with the following methods of Gaussian 16 ver. C: Geometry optimization and frequency analysis: M06-2X/6-31G(d) for $S_0$ and TDA-M06-2X/6-31G(d) for $S_1$. $S_0$-$S_n$ and $S_0$-$T_n$ excitation energies: TDA-M06-2X/6-31G(d). All experiments using animals in this study were performed according to the guidelines provided by the Institutional Animal Care Committee of the Graduate School of Pharmaceutical Sciences, The University of Tokyo (Protocol no. P31-11). All animals were maintained on a 12 h light/dark cycle with food and water ad libitum.

## Materials

$A\beta_{1\text{–}42}$ was prepared from the 26-*O*-acyl isopeptide *in situ* as described in reference [1]. Thioflavin-T (ThT) and insulin (human) were purchased from Sigma-Aldrich, Inc. (St. Louis, MO, USA). Angiotensin-IV (human), somatostatin, and [Tyr$^{8}$]-Substance P were purchased from Peptide Institute,

[1] Taniguchi, A., Shimizu, Y., Oisaki, K., Sohma, Y., and Kanai, M. (2016). Switchable photooxygenation catalysts that sense higher-order amyloid structures. Nat. Chem. 8, 974–982. 10.1038/nchem.2550.

Inc. Leuprorelin acetate was purchased from Tokyo Chemical Industry Co., Ltd. (Tokyo, Japan). α-Synuclein was purchased from ATGen Co., Ltd. (Seongam-si, Gyeonggi-do, Korea). Recombinantly expressed wild-type transthyretin[2] was provided by Prof. Mizuguchi Mineyuki. Other chemical species were purchased from commercial suppliers, Dojin Laboratories (Kumamoto, Japan), Kanto Chemical Co., Inc. (Tokyo, Japan), Nacalai Tesque, Inc. (Kyoto, Japan, Peptide Institute, Inc., Sigma-Aldrich, Inc., Tokyo Chemical Industry Co., Ltd., Wako Pure Chemical Industries, Ltd. (Osaka, Japan), and Watanabe Chemical Industries, Ltd. (Hiroshima, Japan), and were used without further purification.

## Methods

### Oxygenation of Aβ and peptide (protein) selectivity experiment

The reaction was performed similarly to that described in reference 1. In brief, stock solutions of $A\beta_{1-42}$ isopeptide (200 μM in 0.1% aqueous TFA), angiotensin-IV (200 μM in water), somatostatin (200 μM in water), leuprorelin acetate (200 μM in water), [Tyr$^{8}$]-substance P (200 μM in water), and lysozyme (200 μM in water) were diluted with 0.1 mM PB to final peptide concentrations of 20 μM (pH 7.4). For $A\beta_{1-42}$, the solution was incubated at 37 °C for 3 h for aggregation. To each solution, a catalyst (in DMSO) was added. A final concentration of 0.2 μM (1 mol%), 1 μM (5 mol%), or 40 μM (200 mol%) of the catalyst was used for the oxygenation study or peptide (protein) selectivity study. The mixture was irradiated with a light-emitting diode (LED) (λ = 430 nm, 500 nm, 595 nm, or 660 nm) at 37 °C. The powers of the light source were approximately 10 mW (430 nm, 500 nm, and 595 nm) or 5 mW (660 nm), and photoirradiation was performed at a distance of approximately 5 cm away from the samples. Corresponding reaction samples without light irradiation were also prepared as controls. The reactions were monitored and analyzed using MALDI-TOF MS. If necessary, an aliquot of the reaction mixture was de-salted with ZipTip U-C18 (Millipore Corporation) prior to the MS analysis. The degrees of oxygenation were expressed as the intensity ratio of oxygenation (%) = (sum of MS intensities of n[O] adducts) / (sum of MS peak intensities for remaining starting material and n[O] adducts) × 100. For the oxygenation of lysozyme, circular dichroism (CD) spectroscopy of the reaction mixture was measured.

### Selectivity experiment using a mixture of Aβ and other peptides

A stock solution of $A\beta_{1-42}$ isopeptide (200 μM in 0.1% aqueous TFA) was diluted with 0.2 mM PB to a final peptide concentration of 100 μM in 0.1 M PB (pH 7.4). The solution was incubated at 37 °C for 3 h for aggregation. To the solution, angiotensin-IV (200 μM in water), somatostatin (200 μM in

[2] Ueda, M., Okada, M., Mizuguchi, M., Kluve-Becherman, B., Kanenawa, K., Isoguchi, A., Misumi, Y., Tasaki, M., Ueda, A., Kanai, A., et al. (2019). A cell-based high-throughput screening method to directly examine transthyretin amyloid fibril formation at neutral pH. J. Biol. Chem. *294*, 11259–11275. 10.1074/jbc.RA119.007851

water), leuprorelin acetate (200 μM in water), and [$Tyr^8$]-substance P (200 μM in water) were added, and the mixture was diluted with 0.2 M PB to each final concentration of 20 μM in 0.1 M PB (pH 7.4). To the solution, **ABB** (2 mM in DMSO), **EV** (50 μM in DMSO), or **LEV** (100 μM in DMSO) was added. The final concentration of 40 μM for **ABB**, 1 μM for **EV**, or 2 μM for **LEV** was used. The oxygenation and analysis were conducted as described above.

**Identification of oxygenation position**

The oxygenation reaction with **EV** (5 mol%) for 120 min was conducted as described above. To the oxygenation reaction mixture, trypsin, Asp-N, or Glu-C was added and the mixture was incubated at 37 °C for 1 d. The resulting mixture was analyzed by MALDI-TOF MS.

**Absorption spectrum of catalysts**

A catalyst (2 mM in DMSO) was added to 0.1 M PB (final concentration: 20 μM), and the absorption spectrum was measured.

**Fluorescence spectrum of catalysts**

The solution for "**Absorption spectrum of catalysts**" was diluted twice with a 0.1 M PB solution, and the fluorescence spectrum was measured. The excitation wavelength was 550 or 600 nm.

**Absorption spectrum of catalysts in the presence of Aβ**

A 0.1 M PB solution (pH 7.4) containing a catalyst (20 μM) with or without $Aβ_{1–42}$ (20 μM) was incubated at 37 °C for 1 h, and the absorption spectrum was measured.

**Fluorescence spectrum of catalysts in the presence of Aβ**

The solution for "**Absorption spectrum of catalysts in the presence of Aβ**" was diluted twice with a 0.1 M PB solution, and the fluorescence spectrum was measured. The excitation wavelength was 550 nm (for **LEV**) or 600 nm (for **EV**).

**Quartz crystal microbalance (QCM)**

To a gold electrode sensor housed in the cell, 10 μL of 5% EtOH/water including 0.1 mM 20-(11-mercaptoundecanyloxy)-3,6,9,12,15,18-hexaoxaeicosanoic acid was added, and the solution was allowed to stand at room temperature for 1 h. The sensor was rinsed with water and dried with air blow. Then, to the resulting sensor with a self-assembled monolayer on the surface, 50 μL of an aqueous solution including *N*-(3-dimethylaminopropyl)-*N*'-ethylcarbodiimide hydrochloride (50 mg $mL^{-1}$) and *N*-hydroxy succinimide (50 mg $mL^{-1}$) was added, and the solution was allowed to stand at room temperature for 15 min. The sensor was rinsed with water and dried with air blow. After the sensor

cell was installed into the quartz crystal microbalance apparatus, preaggregated A$\beta_{1–42}$ in phosphate buffer was added to the sensor unit (final volume: 20 µL, 50 µM Aβ, 10 mM phosphate). The solutions were stirred at 25 °C until the frequencies become stable. After the sensors were washed with water, 200 µL of 10 mM PB (pH 7.4) was added. When a stable baseline was attained, 0.5 µL of DMSO solution containing a catalyst (0.1, 0.5, and 2 mM) was added, and the changes in the frequencies were measured. The binding curves were generated using KaleidaGraph 4.5. The concentration of a catalyst that elicited one-half of the maximum change of the frequency was calculated and assigned as the $K_d$ value.

**Assessment of the production of $^1O_2$ with aggregated Aβ**

A PB solution (pH 7.4) containing preaggregated Aβ (20 µM), furfuryl alcohol (2 mM), and a catalyst (0.2 mM) was photoirradiated (595 nm LED) at 37 °C for arbitrary periods (0 h, 2 h, 4 h), and the concentrations of furfuryl alcohol were quantified by UV absorbance using HPLC.

HPLC method: acetonitrile for 2 min, followed by a linear gradient of 0–100% acetonitrile over 20 min, and 100% acetonitrile for 5 min Triart C18 column, 215 nm.

**Assessment of the production of $^1O_2$ with glycerol/MeOH solvents**

A solution of furfuryl alcohol (2 mM) and a catalyst (0.2 mM) in glycerol/MeOH (1:1) was photoirradiated (595 nm LED) at 37 °C for arbitrary periods (0 h, 2 h, 4 h), and the concentrations of furfuryl alcohol were quantified by UV absorbance using HPLC.

HPLC method: acetonitrile for 2 min, followed by a linear gradient of 0–100% acetonitrile over 20 min, and 100% acetonitrile for 5 min Triart C18 column, 215 nm.

**Preparation and oxygenation of amyloids**

The reaction was performed similarly to that described in reference 1.

<u>Tau</u>

Preparation: Recombinant Tau Repeat Domain (wild type) (RDWT; the aa 244–332 fragment in full-length tau 2N4R comprising 441 amino acids) was prepared similarly as that described in reference 3. Briefly, RDWT tau was expressed in Rosetta (DE3) (Novagen) and purified using a cation exchange chromatography column with cellulose phosphate as previously reported[4]. After elution, the eluates were applied to reverse-phase chromatography to increase the purity. After evaporation of the elution buffer from reverse-phase chromatography, pellets were dissolved in PBS. The concentration of tau was determined by the bicinchoninic acid (BCA) method. The sample was stocked at –80 °C until use.

Oxygenation: To a solution of tau RDWT (40 μM) in sodium acetate buffer (pH 7.0), 1,4-dithiothreitol (2 μM) and heparin-sodium (40 μM) were added, and the mixture was incubated at 37 °C for 3 h. A catalyst (2 μM) was added and the mixture was photoirradiated (595 nm LED) at 37 °C for 1 h. Urea was added to the reaction mixture to 8 M and products were analyzed using MALDI-TOF MS. The reaction mixture was de-salted with ZipTip U-C18 (Millipore Corporation) prior to the MS analysis. The degrees of oxygenation were expressed as the intensity ratio of oxygenation (%) = (sum of MS intensities of n[O] adducts) / (sum of MS peak intensities for remaining starting material and n[O] adducts) × 100.

<u>Insulin</u>

Preparation: Insulin was dissolved in 25 mM aqueous HCl to adjust the concentration at 400 μM and the solution was filtered (φ = 0.45 μm). The final concentration was determined by UV absorption (276 nm, 6211 $M^{-1}cm^{-1}$) and the sample was stored at −80 °C until use.

Oxygenation: Neutralized buffer solutions containing insulin (100 μM; aggregated samples were prepared via incubation of insulin (400 μM) in 25 mM aqueous HCl solution at 60 °C for 24 h). Adding a catalyst (5 μM), the mixture was photoirradiated (595 nm LED) at 37 °C for 1 h. The reaction mixture was analyzed using MALDI-TOF MS. The reaction mixture was de-salted with ZipTip U-C18 (Millipore Corporation) prior to the MS analysis. The degrees of oxygenation were expressed as the intensity ratio of oxygenation (%) = (sum of MS intensities of n[O] adducts) / (sum of MS peak intensities for remaining starting material and n[O] adducts) × 100.

---

[3] Suzuki, T., Hori, Y., Sawazaki, T., Shimizu, Y., Nemoto, Y., Taniguchi, A., Ozawa, S., Sohma, Y., Kanai, M., Tomita, T. (2019). Photo-oxygenation inhibits tau amyloid formation. Chem. Commun. *55*, 6165–6168. 10.1039/C9CC01728C

[4] Goedert, M. and Jakes, R. (1990). Expression of separate isoforms of human tau protein: correlation with the tau pattern in brain and effects on tubulin polymerization. EMBO J. *9*, 4225–4230. 10.1002/j.1460-2075.1990.tb07870.x

α-Synuclein

Preparation: A commercially available solution of α-synuclein [69 μM in 20 mM Tris-HCl buffer (pH 7.5), 100 mM NaCl, and 1 mM $MgCl_2$] was stored at −80 °C until use.

Oxygenation: A solution containing α-synuclein (69 μM; an aggregated sample was prepared via incubation at 37 °C for 24 h) and a catalyst (3.5 μM) was photoirradiated (595 nm LED) at 37 °C for 1 h. The reaction mixture was treated with Trypsin Gold and analyzed using MALDI-TOF MS. The reaction mixture was de-salted with ZipTip U-C18 (Millipore Corporation) prior to the MS analysis. The degrees of oxygenation were expressed as the intensity ratio of oxygenation for peptide fragments 3 (the aa 103–140 fragment in full-length α-synuclein comprising 140 amino acids) (%) = (sum of MS intensities of n[O] adducts) / (sum of MS peak intensities for remaining starting material and n[O] adducts) × 100.

Transthyretin

Preparation: Recombinant wild-type transthyretin (WT-TTR) with a His6-tagged *N*-terminus was dissolved in PBS to 146 μM and the solution was stocked at –80 °C until use in a similar manner as that described in reference 2.

Oxygenation: To a solution of recombinant WT-TTR (73 μM), AcOH buffer (40 mM) and a KCl solution (100 mM) were added and the mixture was incubated at 37 °C for 48 h. After the incubation, the mixture was neutralized to pH 7 with $NH_3$ aqueous solution to obtain a solution of aggregated WT-TTR (37 μM). To the solution, a catalyst (1.85 μM) was added and the mixture was photoirradiated (595 nm LED) at 37 °C for 1 h. The reaction mixture was treated with Trypsin Gold and products were analyzed using MALDI-TOF MS. The reaction mixture was de-salted with ZipTip U-C18 (Millipore Corporation) prior to the MS analysis. The degrees of oxygenation were expressed as the intensity ratio of oxygenation for peptide fragment 9 (the aa 81–103 fragment in the full-length TTR comprising 127 amino acids) (%) = (sum of MS intensities of n[O] adducts) / (sum of MS peak intensities for remaining starting material and n[O] adducts) × 100.

**Thioflavin-T (ThT) assay**

The ThT assay was examined similarly to that described in reference 1. Briefly, 10 μL of the reaction mixture (20 μM Aβ and 1 μM catalyst in 0.1 M PB) was added to a 1.25 μM ThT solution [400 μL, freshly prepared by adding 50 μM ThT in water (10 μL; ThT was purchased from Sigma-Aldrich Inc.) to a 50 mM glycine-NaOH buffer (396 μL, pH 8.5)]. The fluorescence intensity of the solution (400 μL) was measured by excitation at 440 nm light and detection of the emission fluorescence at 480 nm at r.t..

**Conversion of LEV to EV by photoirradiation**

To a 20 μM **LEV** solution in 0.1 M PB (pH 7.4), aggregated Aβ (0 or 20 μM) was added. The solution was photoirradiated at 430, 500, 595, or 660 nm at 37 °C for a certain time. After irradiation, the absorption spectra of the solution were obtained. The resulting solution was diluted twice by 0.1 M PB, and the fluorescence spectrum was measured with the excitation wavelength at 600 nm. The conversion was also analyzed using LC-MS.

**$^3O_2$ control experiment for the conversion of LEV to EV**

**LEV** (20 μM) was dissolved in 0.1 M PB (degassed with the freeze-pump-thaw method) and the solution was photoirradiated for 30 min. After photoirradiation, absorption spectra of the resulting solution were obtained.

**Effect of $^1O_2$ on the conversion of LEV to EV**

External $^1O_2$ generation

To a 20 μM **LEV** solution in 0.1 M PB (pH 7.4), $H_2O_2$ (2 mM) and $Na_2MoO_4$ (2 mM)[5,6] were added and the mixture was incubated at 37 °C for 60 min. After incubation, the absorption spectrum of the solution was obtained.

Control experiment

To a 20 μM **LEV** solution in 0.1 M PB (pH 7.4), $NaN_3$ (20 μM) as a trapping agent of $^1O_2$ was added and the mixture was incubated at 37 °C for 60 min. After incubation, the absorption spectrum of the solution was obtained.

**Hydrogen atom transfer (HAT) process of DPPH**

To a 20 μM **LEV** solution in 0.1 M PB (pH 7.4), 2,2-diphenyl-1-picrylhydrazyl (DPPH) radical (20 μM) was added and the mixture was incubated at 37 °C for 60 min. After incubation, the absorption spectrum of the solution was obtained.

**Detection of $H_2O_2$**

A 100 μM **LEV** solution in 0.1 M PB (pH 7.4) was photoirradiated (595 nm) at 37 °C for 60 min. After photoirradiation, potassium iodide (4000 eq.) and potassium hydrogen phthalate (1000 eq.) as an acid were added, and the mixture was incubated at r.t. for 30 min. After incubation, the absorption

---

[5] Boehme, K. and Brauer, H. D. (1992). Deneration of singlet oxygen from hydrogen peroxide disproportionation catalyzed by molybdate ions. Inorg. Chem. *31*, 3468–3471. 10.1021/ic00042a024.
[6] Pham, D., Basu, U., Pohorilets, I., St. Crois, C. M., Watkins, S. C., Koide, K. (2020). Fluorogenic Probe Using a Mislow–Evans Rearrangement for Real-Time Imaging of Hydrogen Peroxide. Angew. Chem. Int. Ed. *59*, 17435–17441. 10.1002/anie.202007104.

spectrum of the solution was obtained. The control experiment was also conducted with no addition of **LEV**.

**ON/OFF experiment**

A 20 μM **LEV** solution in 0.1 M PB (pH 7.4) was photoirradiated for 5 min and then incubated for 55 min in the dark at 37 °C. After that, the absorption spectrum of the solution was obtained.

**Examination of electron-donor-acceptor (EDA) complex formation**

A 20 μM **LEV** and/or **EV** solution in 0.1 M PB (pH 7.4) was prepared and the absorption spectra of the solutions were obtained.

**Examination of exciplex formation**

Fluorescence spectra of a mixed solution of **EV** (20 μM) and **LEV** (0 to 80 μM) in variable concentrations in 0.1 M PB (pH 7.4) were measured. The relationship between the fluorescence intensity of $\lambda_{em}$ and the **LEV**/**EV** molar ratio was plotted as a Stern-Volmer plot.

**Cytotoxicity assay**

Rat pheochromocytoma PC12 cells suspended in DMEM that contained 5% horse serum and 10% fetal bovine serum were seeded at a density of 10,000 cells per 100 μL per well on a poly-D-lysine–coated 96-well plate and incubated at 37 °C under 5% $CO_2$ for 3 days. After removing the medium, the cells were washed with 150 μL serum-free DMEM, and 75 μL DMEM containing 0.1% horse serum was added. Then, the 96-well plate was incubated at 37 °C under 5% $CO_2$ for 1 day. A catalyst as dimethyl sulfoxide solution was added to the cell plate described above (final concentration of the catalyst: 0 to 10 μM). The mixture was irradiated or not irradiated with LED ($\lambda$ = 595 nm, approximately 5 cm distance) for 10 min at 37 °C. The cells were incubated for 48 h at 37 °C under 5% $CO_2$. Cell viability was determined using the cell-count reagent SF (Nacalai Tesque, Inc.) with WST-8.

**BBB penetration**

A solution containing the catalyst (1 mM in 10% DMSO/15% Kolliphor EL/75% PBS, 0.2 mL) was intravenously injected into C57BL/6J mice. The mice were perfused with saline, and the brains were excised at arbitrary time points after the injection. The brain samples were homogenized with 1.0 mL MeCN, and the homogenate was centrifuged at 14,000 g for 5 min at 4 °C. The supernatant was evaporated, the resulting solid was redissolved in 0.1 mL MeCN, and 20 μL of the solution was analyzed by HPLC.

HPLC conditions: 0% acetonitrile for 2 min, followed by a linear gradient of 0–100% acetonitrile over

20 min, and 100% acetonitrile for 5 min, Triart Phenyl column, 600 nm (for **EV** and **14**) or Triart C18, 230 nm (for **LEV**).

**Oxygenation of Aβ in mouse brain lysate**

The brain excised from a 10-month-old *App* knock-in ($App^{NL\text{-}G\text{-}F/NL\text{-}G\text{-}F}$) mouse expressing human Arctic Aβs[7] was homogenized in PBS (containing cOmplete EDTA+ (Roche) and PhosSTOP (Sigma)), and the suspensions were stored at −80 °C until use. The catalyst was added to the PBS-lysate suspension (final 50 μM) and the mixture was photoirradiated with LED (λ = 595 nm (for **EV**, **LEV**, or **ABB**) or 430 nm (Riboflavin)) or kept in the dark at 37 °C for 3 h. After the reaction, the mixture was centrifuged (260,000 *g*, 20 min, 4 °C), and the supernatant was removed. The resulting precipitate was redissolved in DMSO. The samples were separated by SDS-PAGE using a 15% Tris-Tricine gel. Molecular weight was estimated with Precision Plus Protein Standards Dual Xtra (Bio-Rad, California, USA). Western blotting analysis was performed using an anti-Aβ antibody (82E1; IBL).

In addition, the reaction mixture (without fractionation) was analyzed with MALDI-TOF MS with the addition of urea and de-saltation with ZipTip U-C18 (Millipore Corporation) prior to the MS analysis.

**Oxygenation of off-target proteins in mouse brain lysate**

The supernatant of the reaction mixture of "**Oxygenation of Aβ in mouse brain lysate**" was analyzed by SDS-PAGE using a 15% Tris-Tricine gel with CBB staining.

**Oxygenation of Aβ *in vivo* (for 5 days)**

A solution of a catalyst (1 mM in 10% DMSO/15% Kolliphor EL/75% PBS, 0.2 mL, ~8.0 mg/kg) was intravenously injected into 5–6-month-old $App^{NL\text{-}G\text{-}F/NL\text{-}G\text{-}F}$ mice (*n* = 3 experiments, each group) expressing human Arctic Aβ. After 5 min (for **LEV**) or 60 min (for **EV** or **ABB**), mice were irradiated with LED (λ = 595 nm) for 10 min. The operation set (catalyst injection and photoirradiation) was repeated five times over 5 days. At 24 hours after the final operation set, the brain was excised and homogenized using a 10× volume of 1× PBS buffer (containing cOmplete EDTA+ (Roche) and PhosSTOP (Sigma)). After the mixture was centrifuged (260,000 g, 20 min, 4 °C), the supernatant was removed, and the precipitate was homogenized using 2% Triton-X–containing PBS buffer. The mixture was then centrifuged (260,000 g, 20 min, 4 °C), and again, the supernatant was removed. The resulting precipitate was homogenized using 70% formic acid (FA), sonicated, and centrifuged (260,000 g, 20 min, 4 °C). The collected supernatant (=Insoluble fraction) was evaporated, redissolved

---

[7] Saito, T., Matsuba, Y., Mihara, N., Takano, J., Nilsson, P., Itohara, S., Iwata, N., and Saido, T. C. (2014). Single App knock-in mouse models of Alzheimer's disease. Nat. Neurosci. *17*, 661. 10.1038/nn.3697.

in DMSO, and analyzed by SDS-PAGE using a 15% Tris-Tricine gel and Western blotting using an anti-Aβ antibody (82E1; IBL). For loading controls, α-tubulin in lysates before fractionation was analyzed (B-5-1-2; Sigma). The protein subjected to SDS-PAGE was quantified using the BCA method, and the applied amount was normalized.

**Oxygenation of Aβ *in vivo* (for 2 months)**

Using 2–3-month-old $App^{NL\text{-}G\text{-}F/NL\text{-}G\text{-}F}$ mice ($n$ = 3 experiments, each group), the same operation set (catalyst injection and photoirradiation) as "**Oxygenation of Aβ *in vivo* (for 5 days)**" was repeated three times per week for 2 months. At 24 hours after the final operation set, the brain was excised and homogenized using a 10× volume of 1×PBS buffer (containing cOmplete EDTA+ (Roche) and PhosSTOP (Sigma)). The lysate was analyzed by SDS-PAGE using a 15% Tris-Tricine gel and Western blotting using an anti-Aβ antibody (82E1; IBL). The protein subjected to SDS-PAGE was quantified using the BCA method, and the applied amount was normalized. For loading controls, α-tubulin in lysates before fractionation was analyzed (B-5-1-2; sigma). Western blotting for a single sample was performed in triplicate, and the data was shown as the mean (of $n$ = 3 experiments) ± SEM.

**Human brain samples**

Samples of brain tissue from patients with Alzheimer disease were obtained from the tissue bank at the University of Pennsylvania Alzheimer's Disease Core Center (ADCC) and the Center for Neurodegenerative Disease Research (CNDR). All samples used in experiments were obtained from the temporal cortex under the approval of the institutional review board, ADCC-CNDR, and the institutional ethical committee of the Graduate School of Pharmaceutical Sciences, University of Tokyo (No. 2-1).

**Oxygenation of Aβ from lysates of human brain samples**

The temporal cortex of an Alzheimer disease patient was homogenized using 10 × volume of PBS (containing cOmplete EDTA+ (Roche) and PhosSTOP (Sigma)). For photo-oxygenation, a catalyst (2.5 μM) was added to the brain lysate and irradiated with 595 nm light for 3 h or kept in the dark at 37 °C. The resulting mixture was analyzed with SDS-PAGE, Western blotting (anti-Aβ antibodies: 82E1 (IBL), anti-tau antibodies: 5A6 anti-GAPDH antibodies: GAPDH-71.1) and CBB staining.

## Supplemental Figures

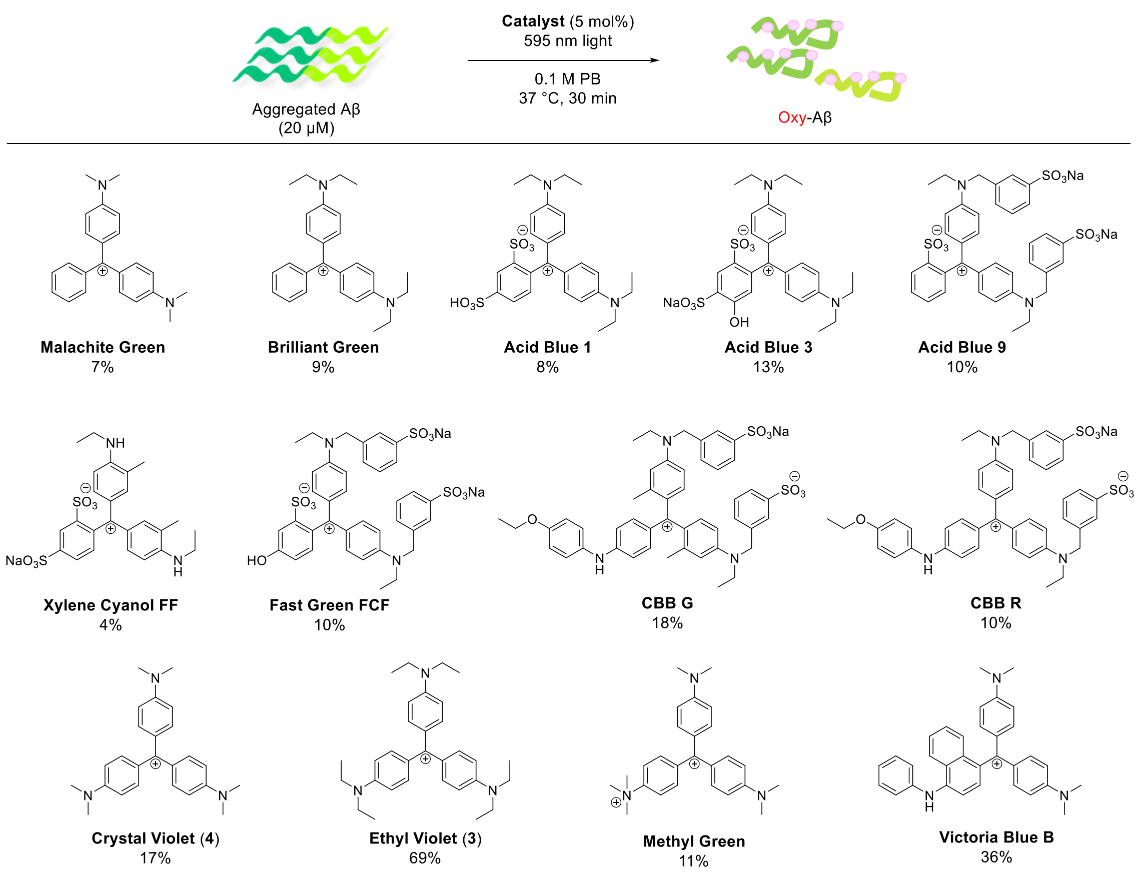


**Figure S1.** Screening of the photooxygenation catalysts. A phosphate buffer solution (pH 7.4) containing aggregated A$\beta_{1-42}$ (20 μM) and catalyst (1 μM, 5 mol%) was photoirradiated ($\lambda$ = 595 nm, 10 mW) at 37 °C for 30 min, and the reaction progress was analyzed using MALDI-TOF MS (average of n = 3 experiments).

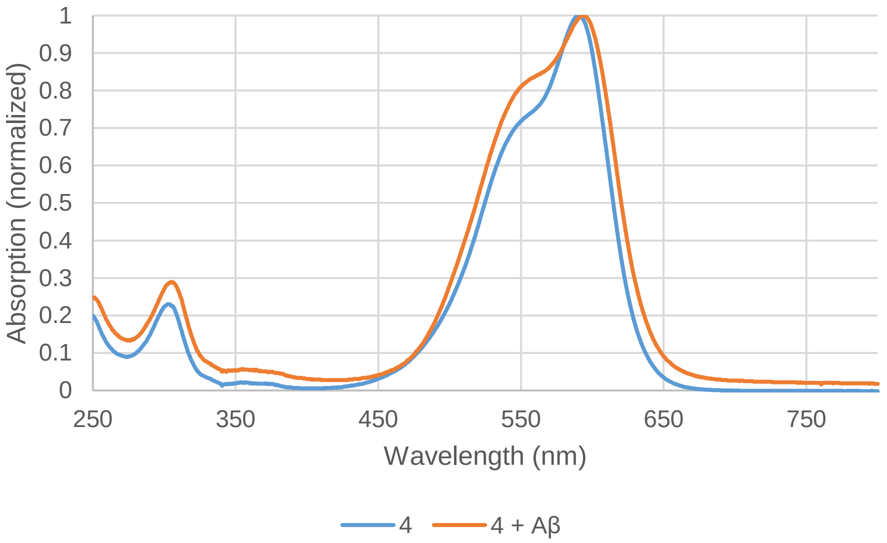
Absorption (normalized)
Wavelength (nm)
0
0.1
0.2
0.3
0.4
0.5
0.6
0.7
0.8
0.9
1
250
350
450
550
650
750
4
4 + Aβ

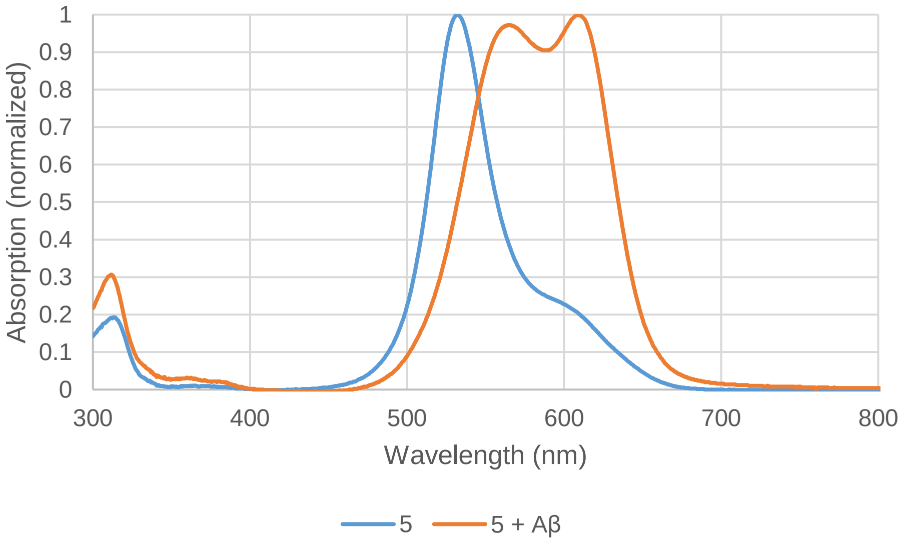
Absorption (normalized)
Wavelength (nm)
0
0.1
0.2
0.3
0.4
0.5
0.6
0.7
0.8
0.9
1
300
400
500
600
700
800
5
5 + Aβ

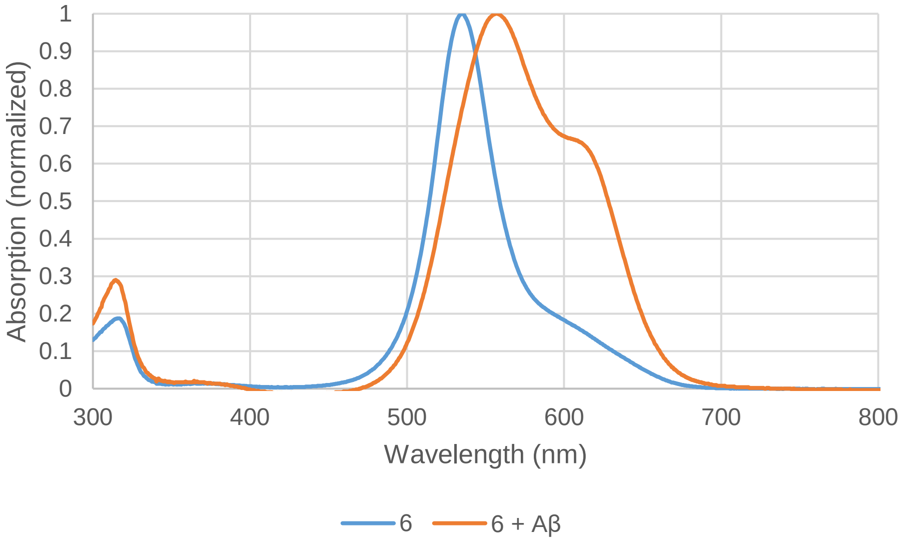
Absorption (normalized)
Wavelength (nm)
0
0.1
0.2
0.3
0.4
0.5
0.6
0.7
0.8
0.9
1
300
400
500
600
700
800
6
6 + Aβ

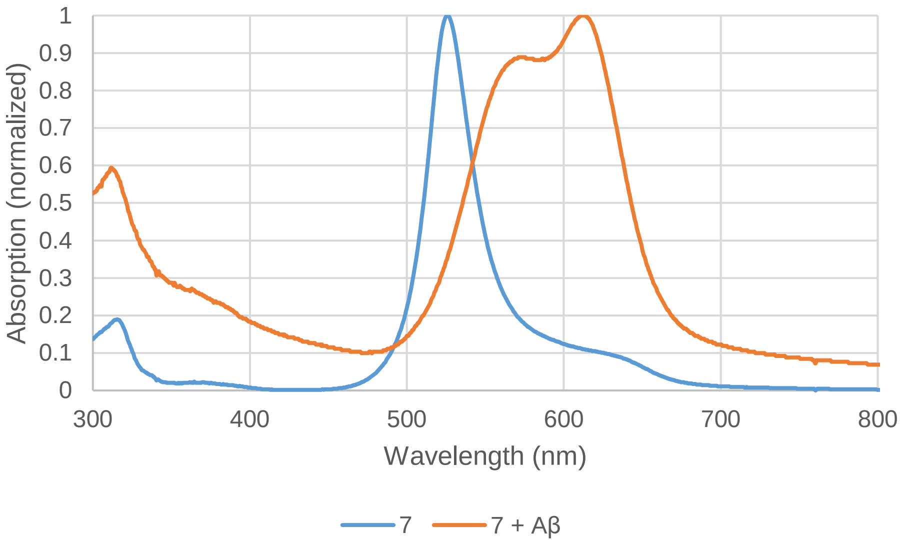

Absorption (normalized)
1
0.9
0.8
0.7
0.6
0.5
0.4
0.3
0.2
0.1
0
300
400
500
600
700
800
Wavelength (nm)
7
7 + Aβ


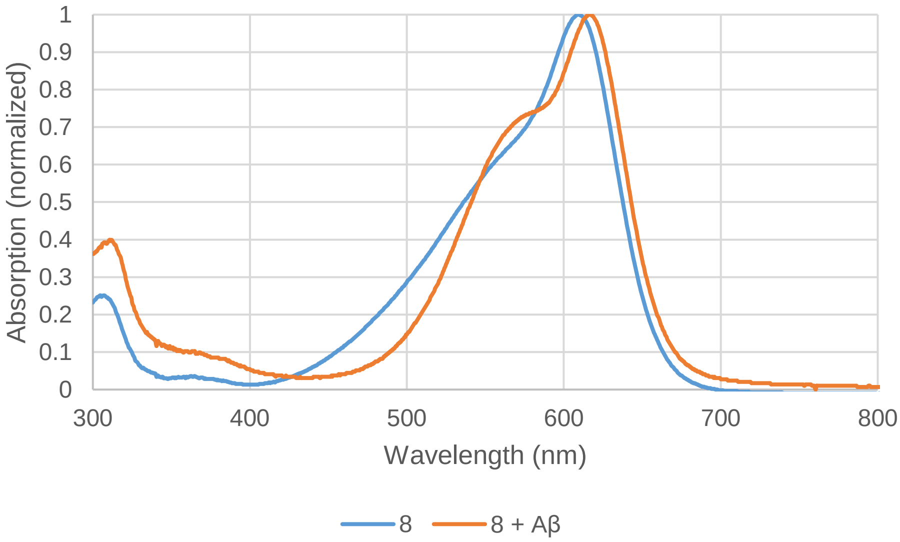

Absorption (normalized)
1
0.9
0.8
0.7
0.6
0.5
0.4
0.3
0.2
0.1
0
300
400
500
600
700
800
Wavelength (nm)
8
8 + Aβ


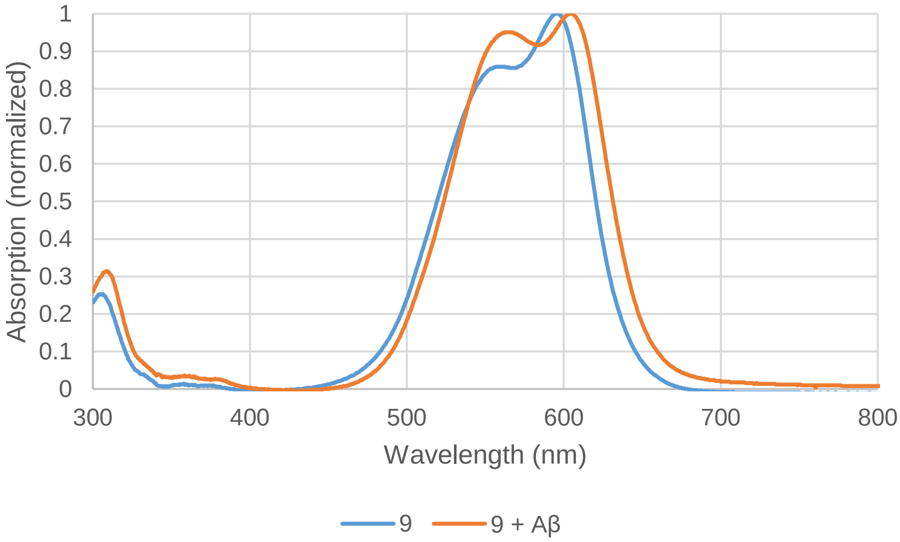

Absorption (normalized)
1
0.9
0.8
0.7
0.6
0.5
0.4
0.3
0.2
0.1
0
300
400
500
600
700
800
Wavelength (nm)
9
9 + Aβ

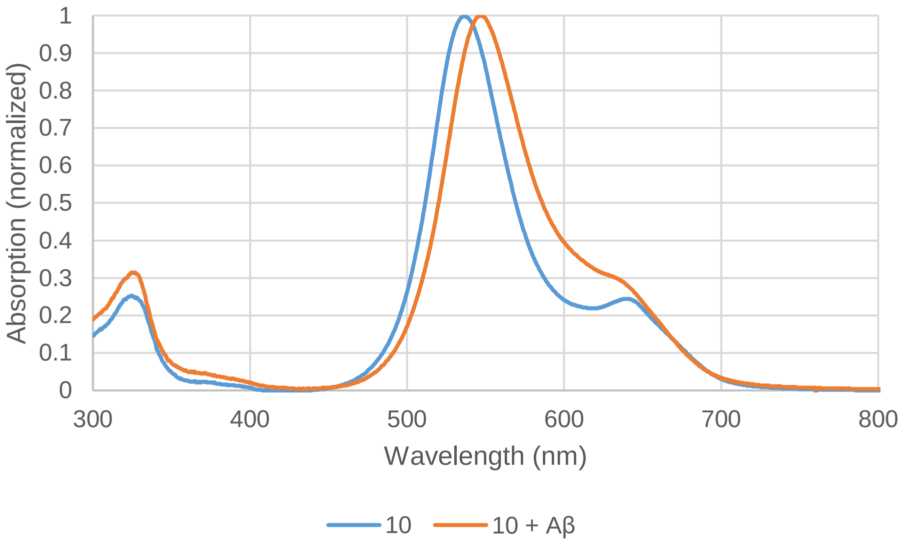
Absorption (normalized)
Wavelength (nm)
10
10 + Aβ

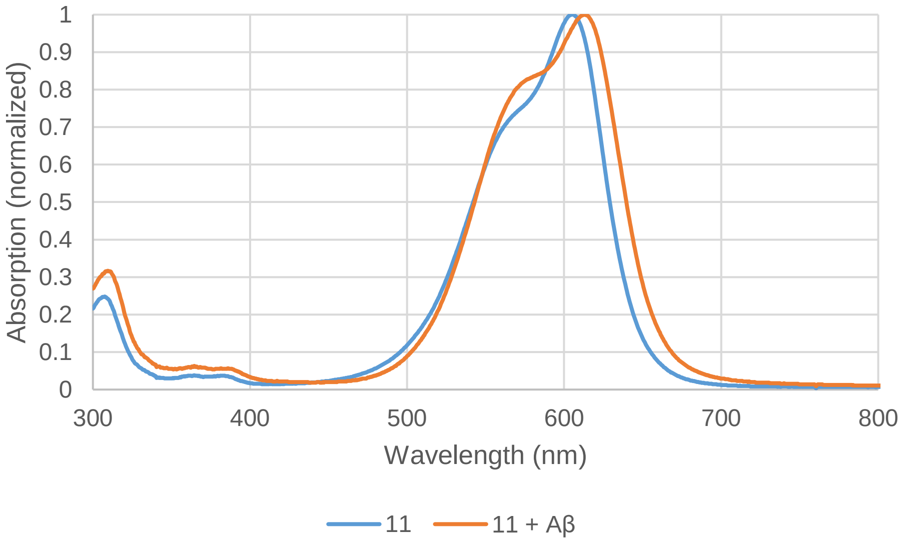
Absorption (normalized)
Wavelength (nm)
11
11 + Aβ

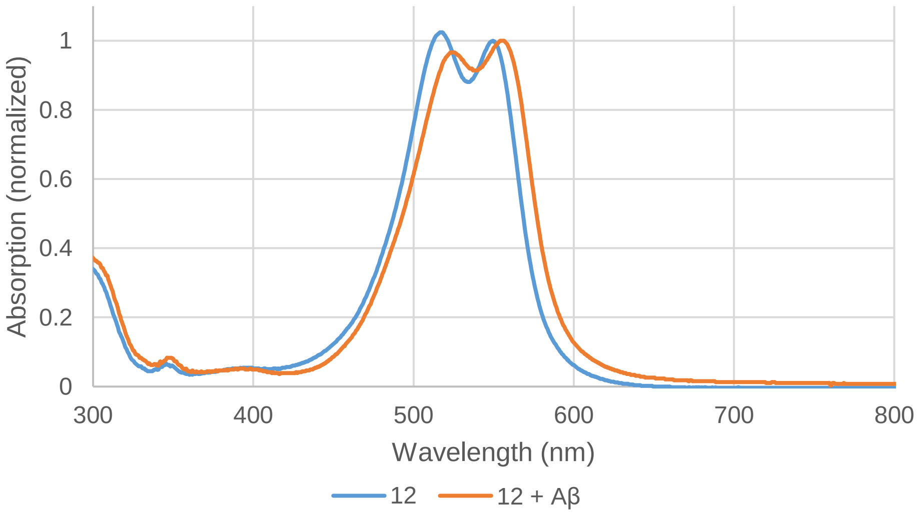
Absorption (normalized)
Wavelength (nm)
12
12 + Aβ

**Figure S2.** Absorption spectra of the triarylmethane catalysts. A 0.1 M PB solution (pH 7.4) containing a catalyst (20 µM) with or without preaggregated A$\beta_{1\text{–}42}$ (20 µM) was incubated at 37 °C for 1 h, and the absorption spectrum was measured.

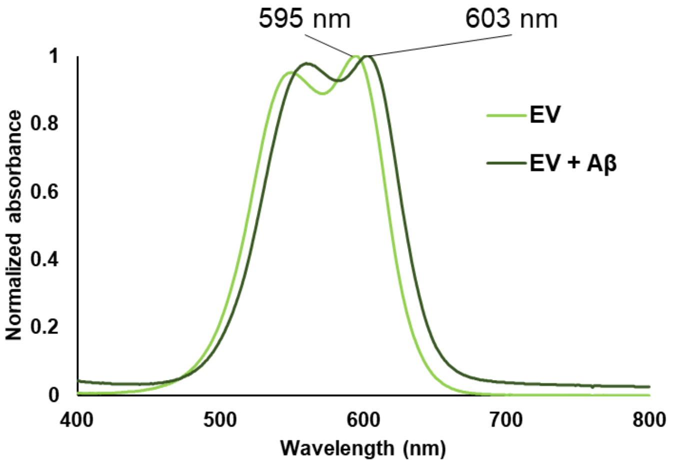


**Figure S3.** Absorption spectra of **EV** with or without aggregated Aβ. A phosphate buffer solution (pH 7.4) containing **EV** (20 µM) with aggregated Aβ (20 µM) was incubated at 37 °C for 1 hour, and the absorption spectrum was measured. In the condition without Aβ, **EV** (2 mM in DMSO) was added to a phosphate buffer solution (pH 7.4, the final concentration of **EV**: 20 µM), and the absorption wavelength was measured.

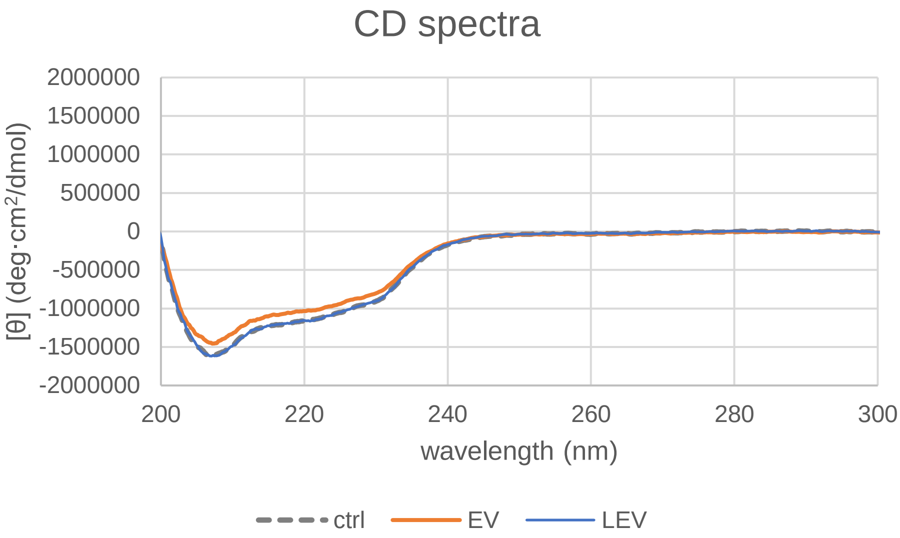


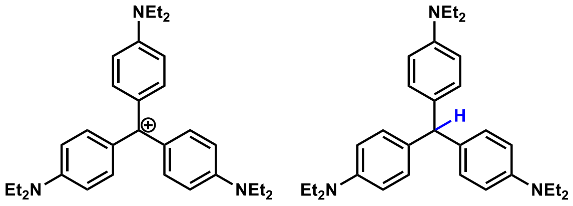


**EV** N.R.

**LEV** N.R.

**Figure S4.** Photooxygenation reaction of an off-target protein with **EV** or **LEV**. A phosphate buffer solution (pH 7.4) containing lysozyme (20 μM) and catalyst (1 μM, 5 mol%) was photoirradiated (λ = 595 nm, 10 mW) at 37 °C for 60 min, and the reaction progress was analyzed using MALDI-TOF MS. N.R. (no reaction) means that no oxygenated lysozyme was detected by MALDI-TOF MS analysis. Circular dichroism (CD) spectroscopy of the reaction mixture also indicates that the tertiary structure of the protein was unchanged before (ctrl) and after the reaction with **EV** and **LEV**.

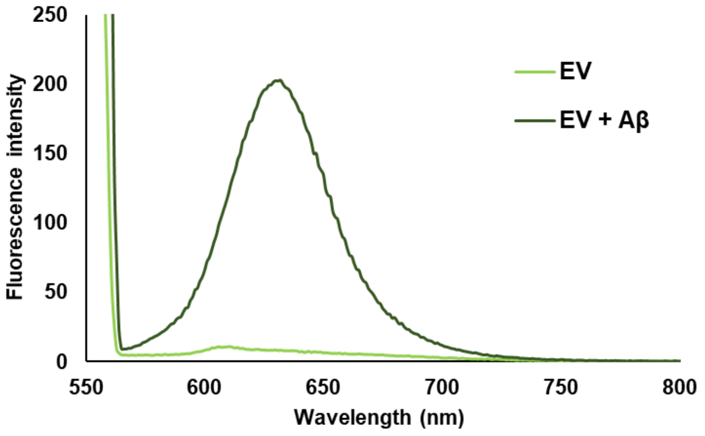


**Figure S5.** Fluorescence spectra of **EV** with or without aggregated Aβ. The solution of Figure S3 was diluted twice with a phosphate buffer solution (pH 7.4), and the fluorescence spectrum was measured. The excitation wavelength was 550 nm.

(a)

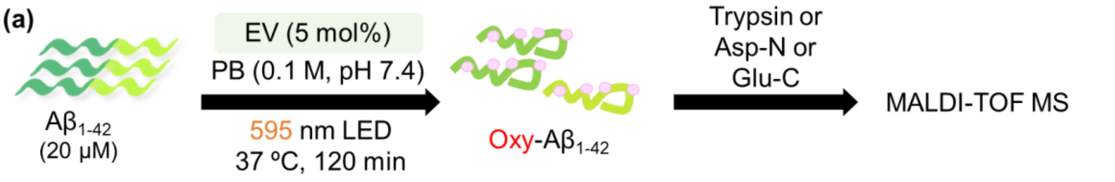


(b)

DAEFR HDSGY EVHHQ KLVFF
AEDVG SNKGA IIGLM VGGVV IA

Oxygenation sites: His$^{6}$, His$^{13}$ or His$^{14}$ , Met$^{35}$

Observed crosslinked product:
- His$^{6}$[O]-Lys$^{16}$ or His$^{13}$[O]-His$^{6}$ or His$^{14}$[O]-His$^{6}$ (same MW)
- His$^{6}$[O]-Lys$^{28}$

**(c)**

## LC-MS

**Trypsin**

F1: DAEFR

F2: HDSGYEVHHQK

F3: LVFFAEDVGSNK

F4: GAIIGLMVGGVVIA

【extracted m/z】

・Met35 in F4 ((1268.76+16.00)/2+1.01=643.4)

Oxygenated Aβ

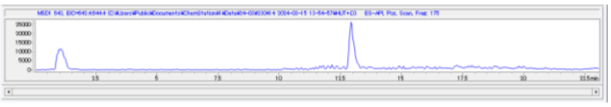

Control Aβ

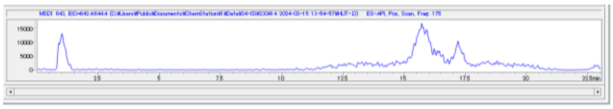

**Asp-N**

F1: DAEFRH

F2: DSGYEVHHQKLVFFAE

F3: DVGSNKGAIIGLMVGGVVIA

【extracted m/z】

・His6 in F1 (774.4+13.98=788.4)

・Met35 in F3 ((1869.04+16.00)/3+1.01=629.4)

・His13or14 in F1-2 ((2660.24+13.98)/4+1.01=669.6)

・Crosslink(F1, F2) or Y[2O] in F1-2 ((2692.3+4.04)/4=674.1)

Oxygenated Aβ

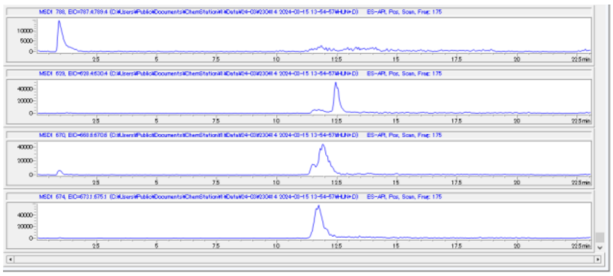

Control Aβ

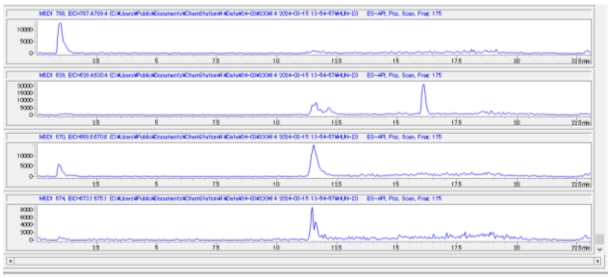

**Glu-C**

F1: D

F2: AE

F3: FRHD

F4: SGYD

F5: VHHQKLVFFAE

F6: D

F7: VGSNKGAIIGLMVGGVVIA

【extracted m/z】

・His6 in F3-4 ((1009.4+13.98)/2+1.01=512.7)

・His13or14 in F5 ((1353.72+13.98)/2+1.01=684.9)

・Met35 in F6-7 ((1869.03+16.00)/3+1.01=629.4)

・His6-Lys28Crosslink(F3, F7) ((2341.3+4.04)/4=586.3)

Oxygenated Aβ

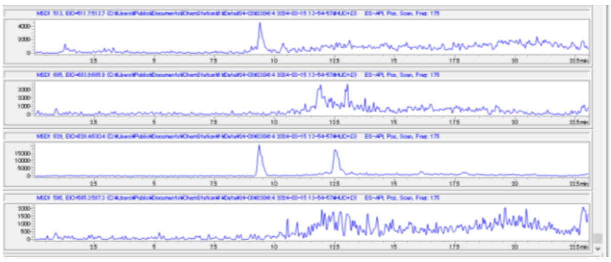

Control Aβ

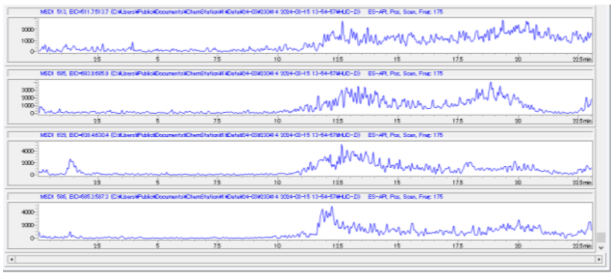

# MALDI-TOF MS

**Trypsin**

F1: DAEFR

F2: HDSGYEVHHQK

F3: LVFFAEDVGSNK

F4: GAIIGLMVGGVVIA

・Met35 in F4 (1268.76+16.00+1.01=1285.77)

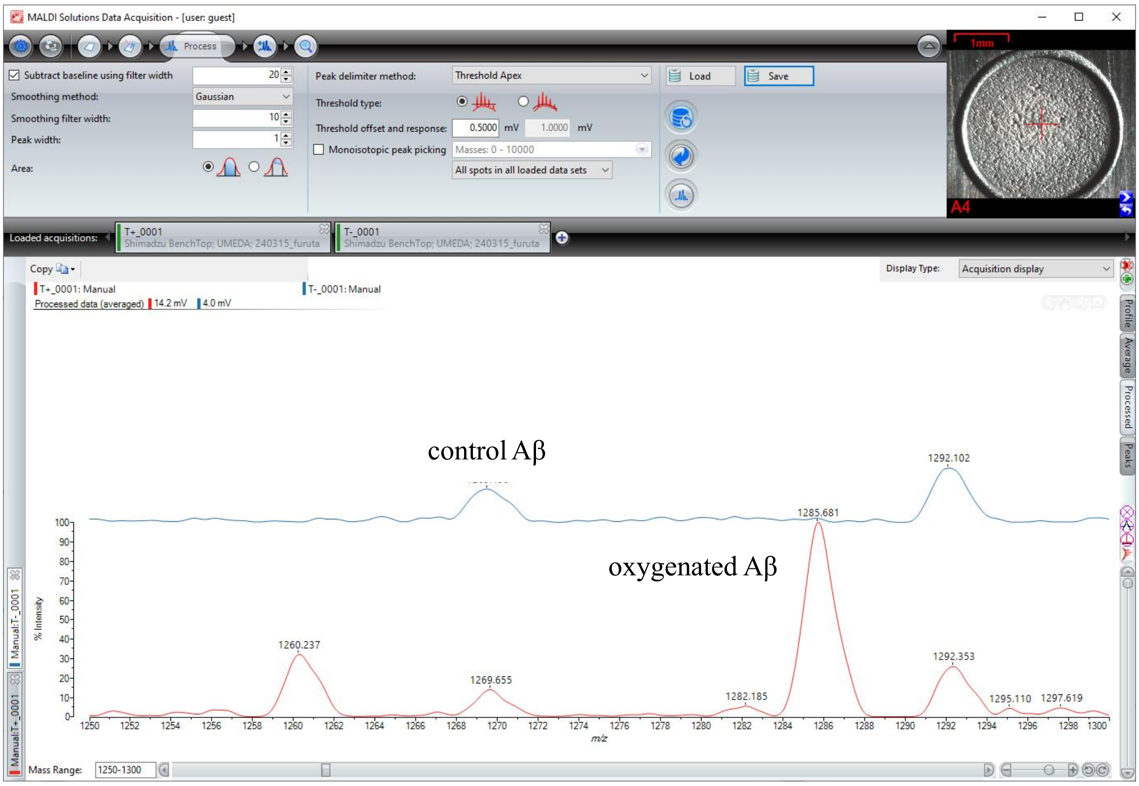


(upper: control Aβ, lower: oxygenated Aβ)

**Asp-N**

F1: DAEFRH

F2: DSGYEVHHQKLVFFAE

F3: DVGSNKGAIIGLMVGGVVIA

・His6 in F1 (774.4+13.98=788.4)

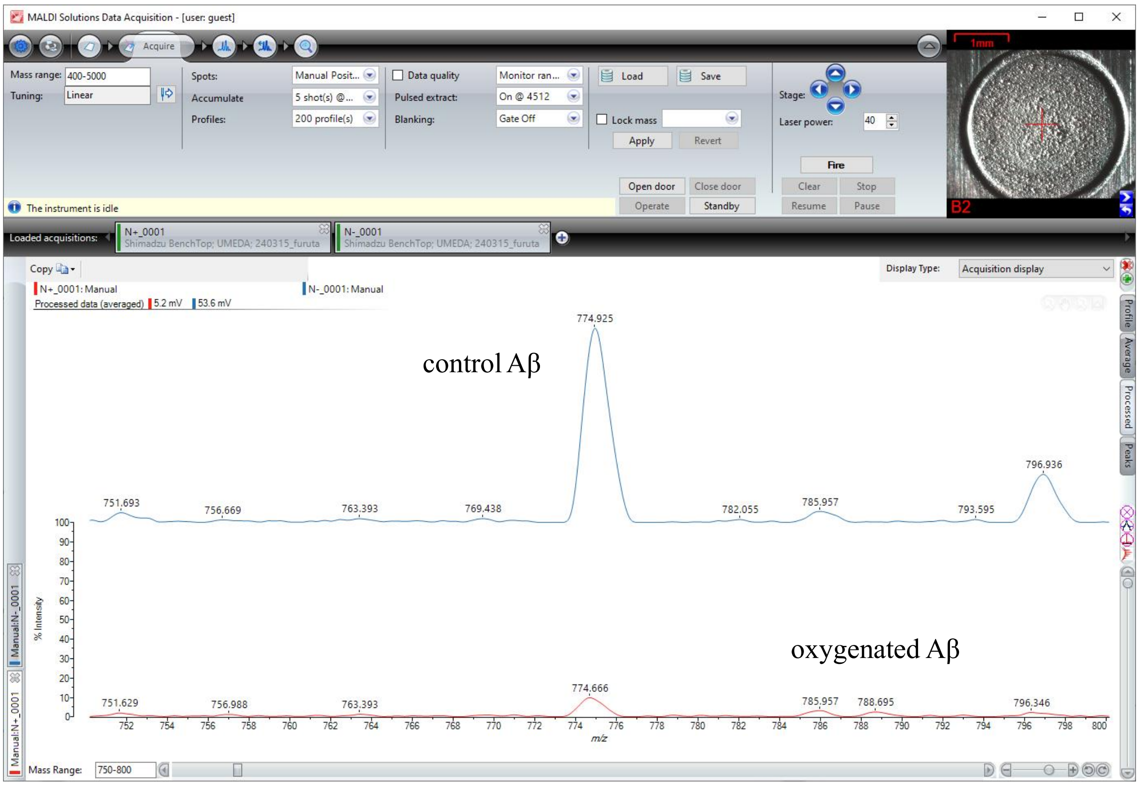


(upper: control Aβ, lower: oxygenated Aβ)

・Met35 in F3 (1869.04+16.00+1.01=1886.05)

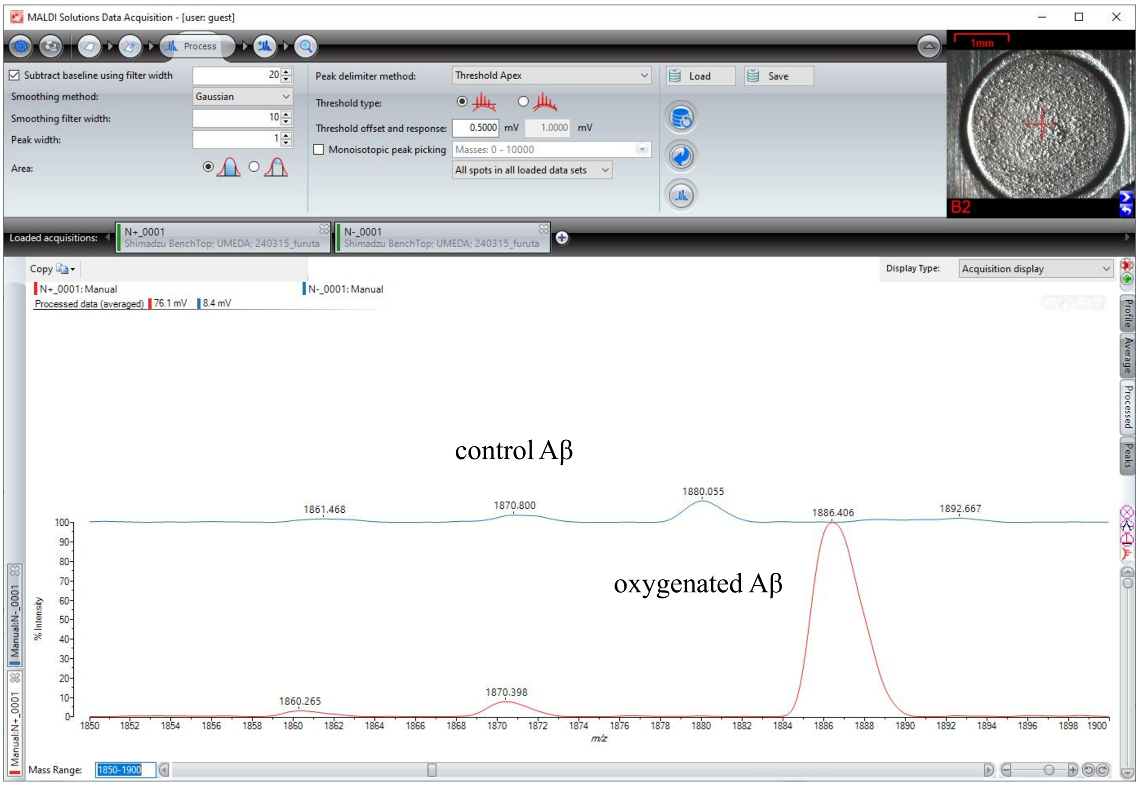


(upper: control Aβ, lower: oxygenated Aβ)

・His13or14 in F1-2 (2660.24+13.98+1.01=2675.23)

・Crosslink(F1, F2) or Y[2O] in F1-2 (2692.3+4.04=2696.34)

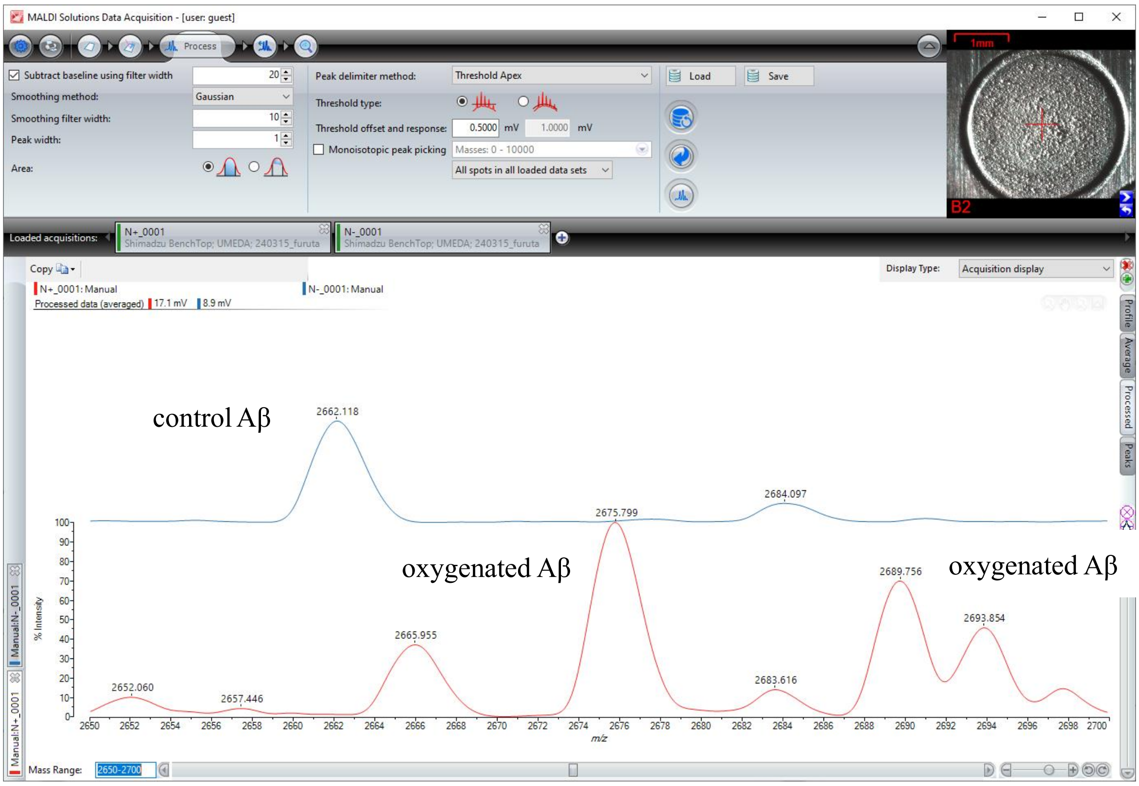


(upper: control Aβ, lower: oxygenated Aβ)

**Glu-C**

F1: D

F2: AE

F3: FRHD

F4: SGYD

F5: VHHQKLVFFAE

F6: D

F7: VGSNKGAIIGLMVGGVVIA

・His6 in F3-4 (1009.4+13.98+1.01=1024.39)

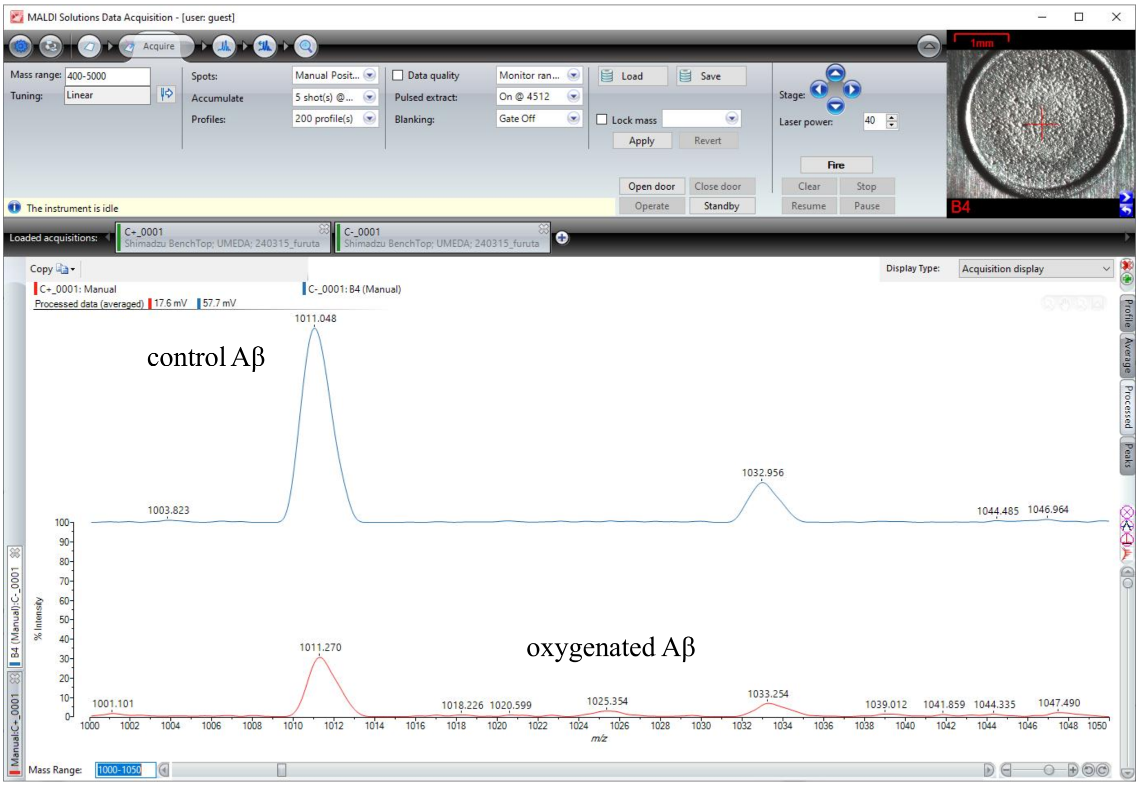


(upper: control Aβ, lower: oxygenated Aβ)

・His13or14 in F5 (1353.72+13.98+1.01=1368.71)

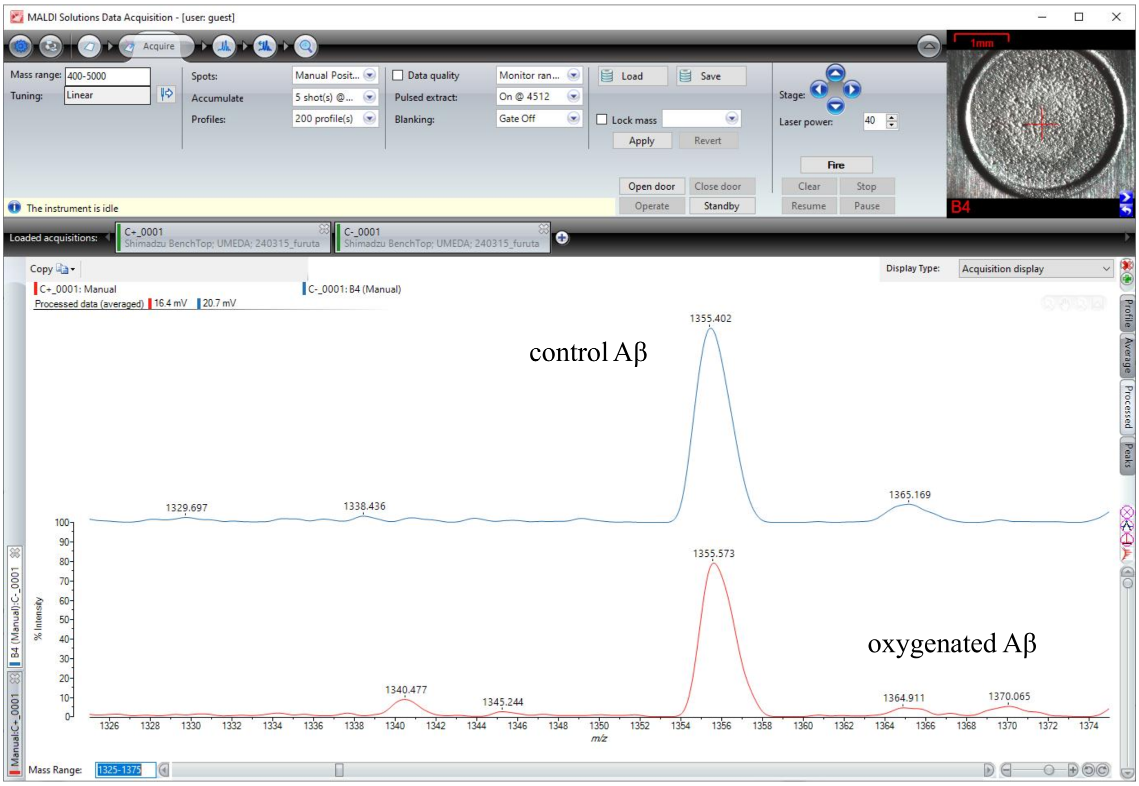


(upper: control Aβ, lower: oxygenated Aβ)

・Met35 in F6-7 (1869.03+16.00+1.01=1886.04)

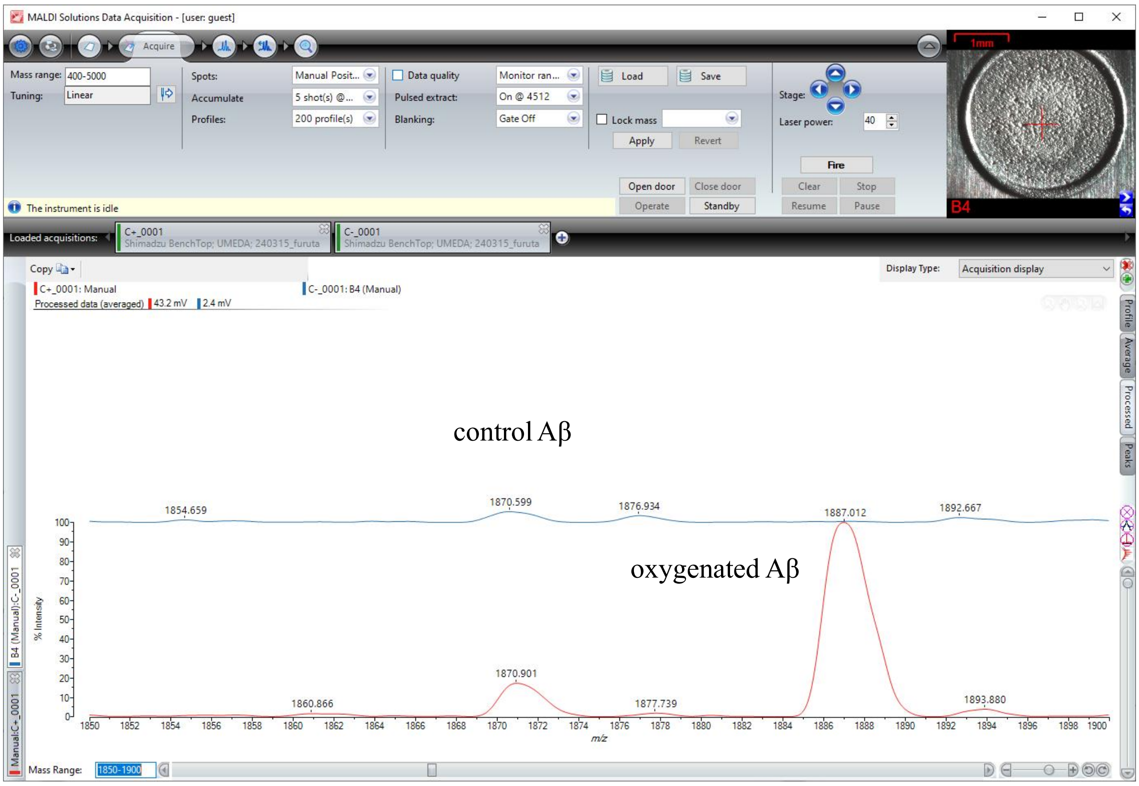


(upper: control Aβ, lower: oxygenated Aβ)

**Figure S6.** Determination of the oxygenated residues of Aβ. (a) Protocol. Oxygenated Aβ was digested with trypsin, Asp-N, or Glu-C. The resulting mixture was analyzed by MALDI-TOF MS. (b) Oxygenated residues. (c) LC-MS data/spectra and MALDI-TOF MS spectra for fragments after trypsinization or enzymatic digestion.

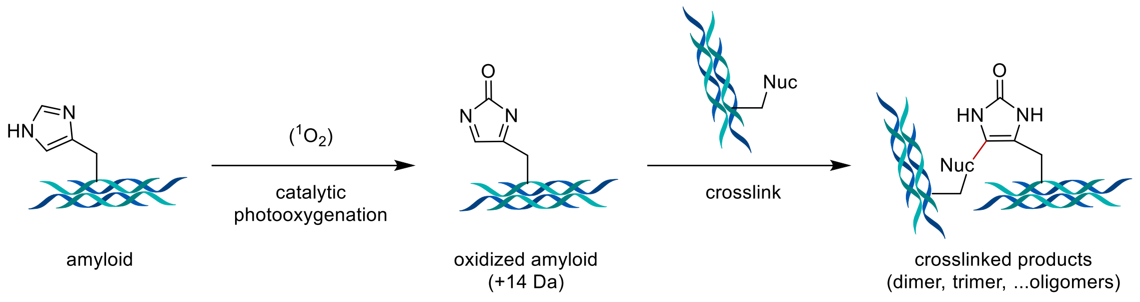


**Figure S7.** Relationship between His oxygenation and crosslink. Singlet oxygen ($^1O_2$) reacts with imidazole of histidine, leading to an electrophilic imidazolone intermediate (oxidized amyloid). The intermolecular nucleophilic addition of amino acid residues (such as His and Lys) to imidazolone produces crosslinked products.

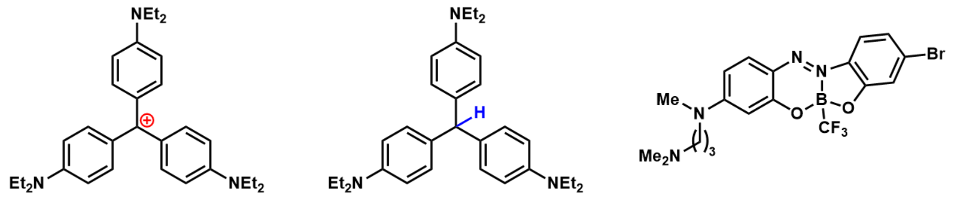


| Catalyst | EV | LEV | ABB |
|---|---|---|---|
| $K_d$ value (μM) | 1.48 ± 0.28 | 2.30 ± 0.29 | 3.40 ± 1.64 |

**Figure S8.** Affinities between aggregated Aβ and catalysts. A quartz crystal microbalance (QCM) study ($n$ = 3 experiments, mean ± SEM) to measure the dissociation constant values of catalysts with aggregated Aβ. $K_d$: dissociation constant.

(A)

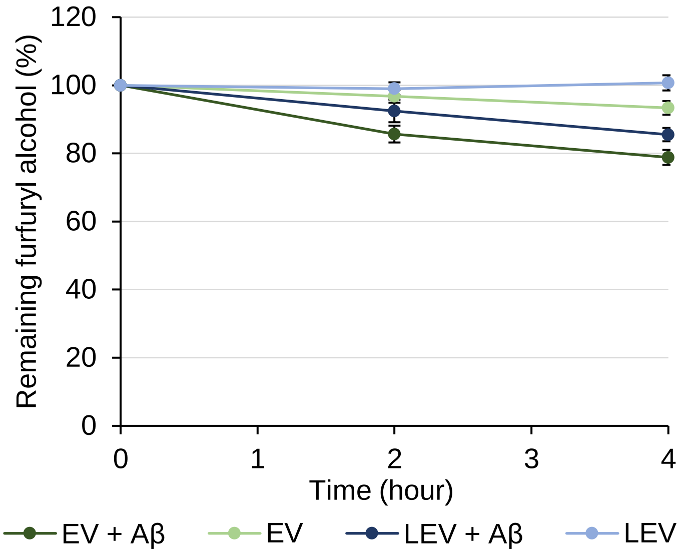


(B)

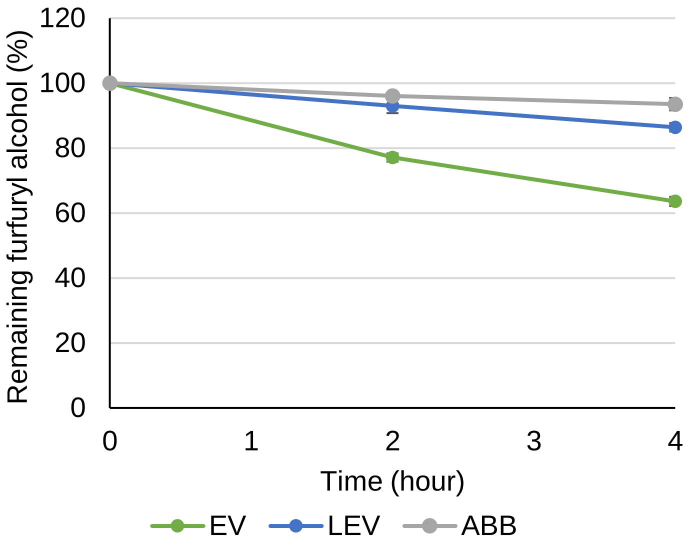


**Figure S9.** Oxygenation of furfuryl alcohol by $^1O_2$. (A) A PB solution (pH 7.4) containing aggregated A$\beta_{1-42}$ (20 µM), furfuryl alcohol (2 mM), and a catalyst **EV** or **LEV** (0.2 mM) was photoirradiated (λ = 595 nm, 10 mW) at 37 °C for arbitrary time periods (0 h, 2 h, and 4 h), and the concentrations of remaining furfuryl alcohol were quantified by UV absorbance using HPLC ($n$ = 3 experiments, mean ± SEM). (B) A mixed solution of furfuryl alcohol (2 mM) and catalyst (200 µM) in a mixed solvent of glycerol/MeOH (1/1) was photoirradiated with 595 nm light at 37 °C for certain time. The concentration of remaining furfuryl alcohol was measured with UV absorption.

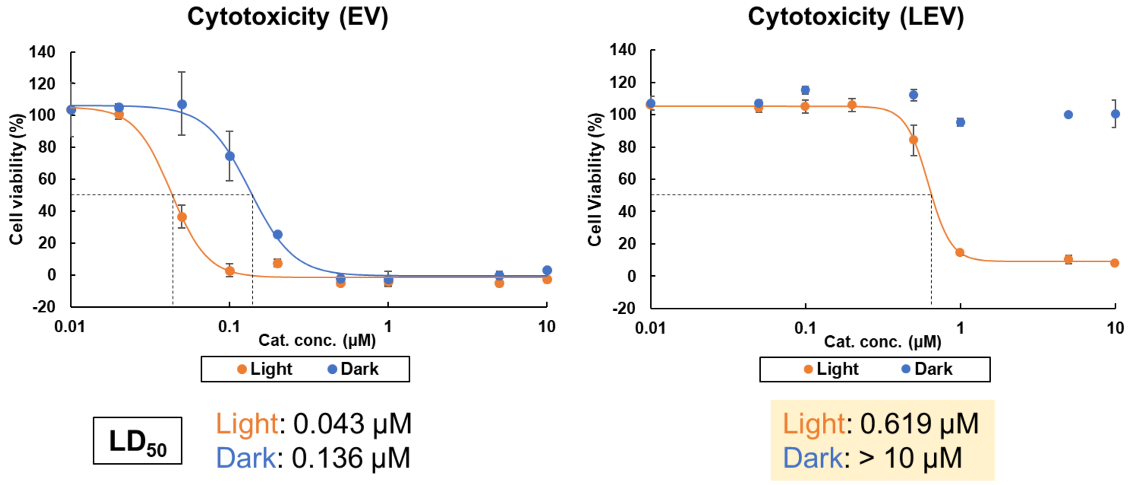


**Figure S10.** Cell viability assay. To PC12 cells in DMEM, a catalyst in PBS was added. Under the "Light" conditions, the cells were photoirradiated with 595 nm light at 37 °C for 10 min. After 2-day incubation at 37 °C, cell viability was determined using the Cell Count Reagent SF with WST-8 ($n$ = 3 experiments, mean ± SEM).

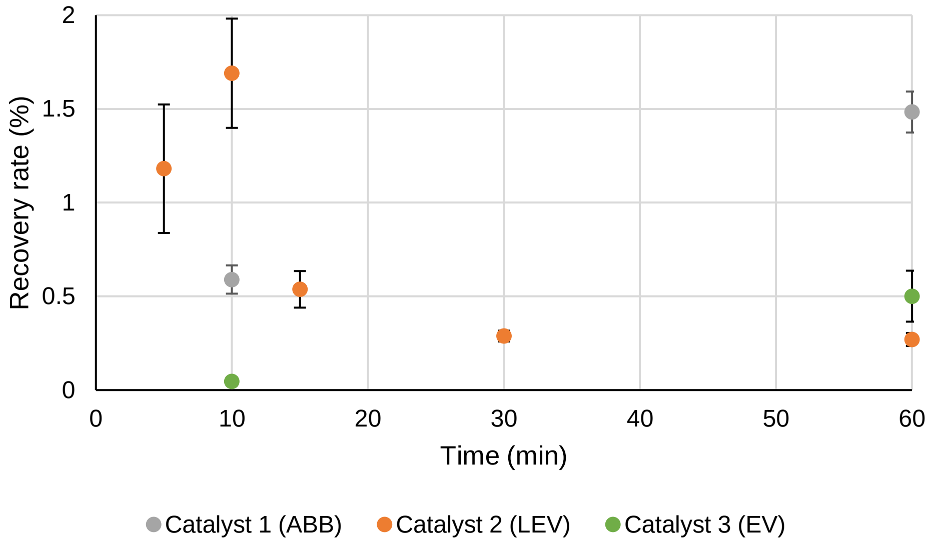


**Figure S11.** BBB permeability of the catalysts. A solution containing the catalyst (1 mM in 10% DMSO/15% Kolliphor EL/75% PBS, 200 µL) was intravenously injected into C5BL6J mice. The mice were perfused with saline, and the brains were excised at arbitrary time points after injection. The brain samples were homogenized with 1.0 mL MeCN, and the homogenate was centrifuged at 14,000 $g$ for 5 min at 4 °C. The supernatant was taken up to another vessel and evaporated. The resulting solid was redissolved in 100 µL MeCN, and the solution was analyzed by HPLC ($n$ = 3 experiments, mean ± SEM).

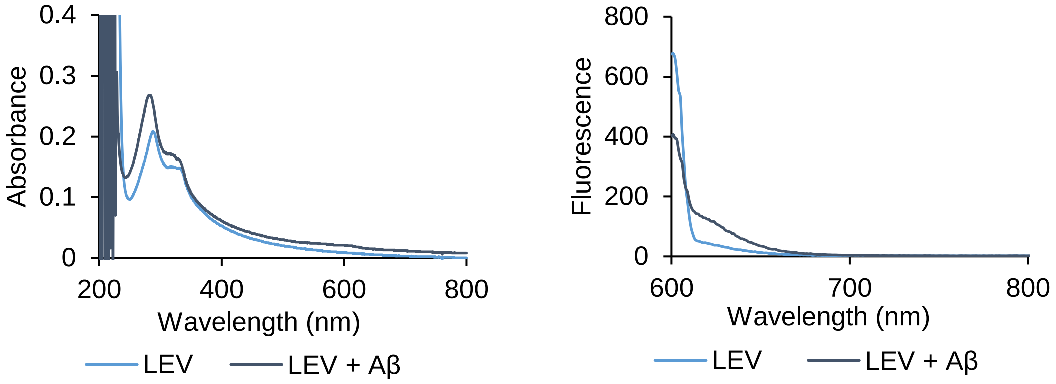


**Figure S12.** Photochemical properties of **LEV**. (Left) Absorption spectra of **LEV** with or without aggregated Aβ. A phosphate buffer solution (pH 7.4) containing **LEV** (20 μM) with aggregated Aβ (20 μM) was incubated at 37 °C for 1 hour, and the absorption spectrum was measured. Under the conditions without Aβ, **LEV** (2 mM in DMSO) was added to a phosphate buffer solution (pH 7.4, the final concentration of **LEV**: 20 μM), and the absorption wavelength was measured. (Right) Fluorescence spectra of **LEV** with or without aggregated Aβ. The solution for the absorption spectrum measurement was diluted twice with a phosphate buffer solution (pH 7.4), and the fluorescence spectrum was measured. The excitation wavelength was 600 nm.

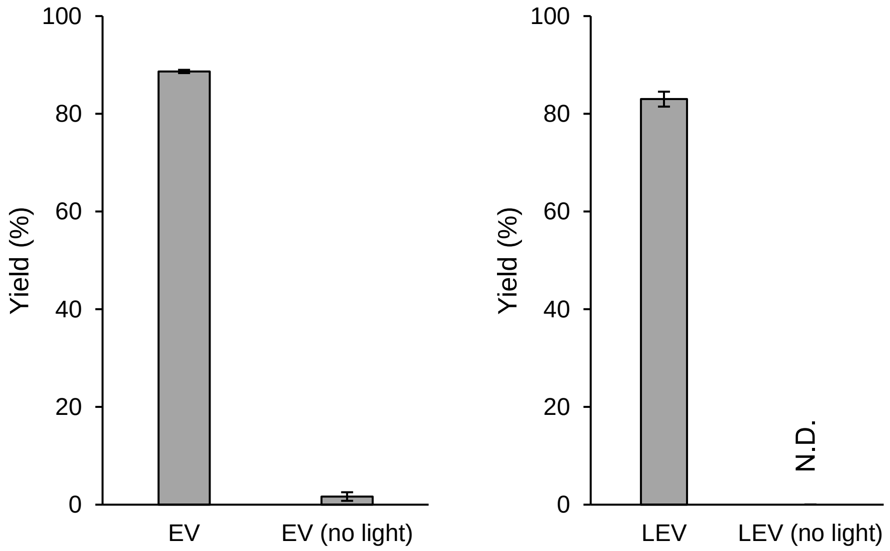


**Figure S13.** Comparison between irradiated and non-irradiated conditions. A PB solution (pH 7.4) containing $A\beta_{1-42}$ (20 μM) and catalyst (1 μM, 5 mol%) was photoirradiated (λ = 595 nm, 10 mW) at 37 °C for 120 min, and the reaction progress was analyzed using MALDI-TOF MS (average of n = 3 experiments).

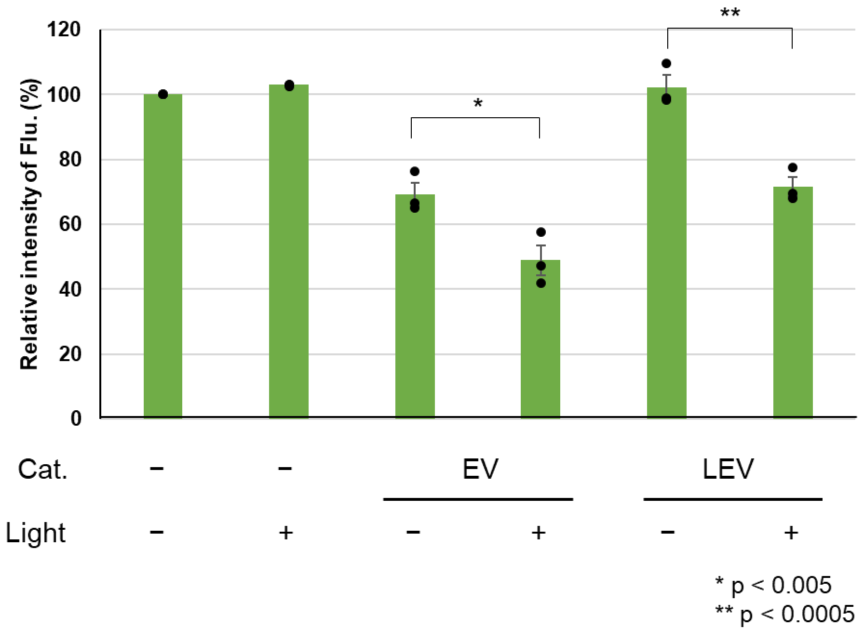


**Figure S14.** Aggregation assay using **EV** or **LEV**. Catalyst (5 mol%) was added to monomer Aβ, and photooxygenation was performed for 3 hours. After the reaction, the aggregation level was evaluated with Thioflavin T (ThT) fluorescence.

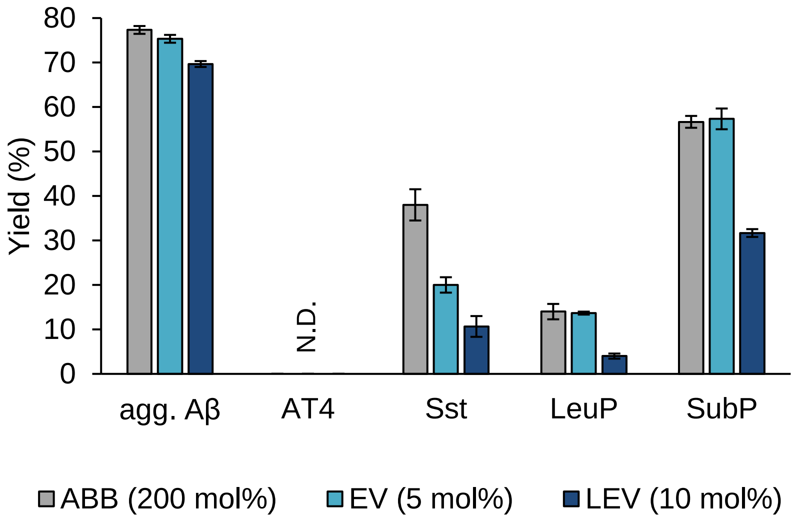


**Figure S15.** Photooxygenation reaction of a mixture of aggregated Aβ and off-target peptides. To a mixed solution of AT4, Sst, LeuP, SubP, and aggregated Aβ (20 μM each), a catalyst [ABB (40 μM, 200 mol%), **EV** (1 μM, 5 mol%), or **LEV** (2 μM, 10 mol%)] was added. The mixture was photoirradiated (λ = 595 nm, 10 mW) at 37 °C for 60 min. After photoirradiation, the reaction progress was analyzed using MALDI-TOF MS ($n$ = 3 experiments, mean ± SEM).

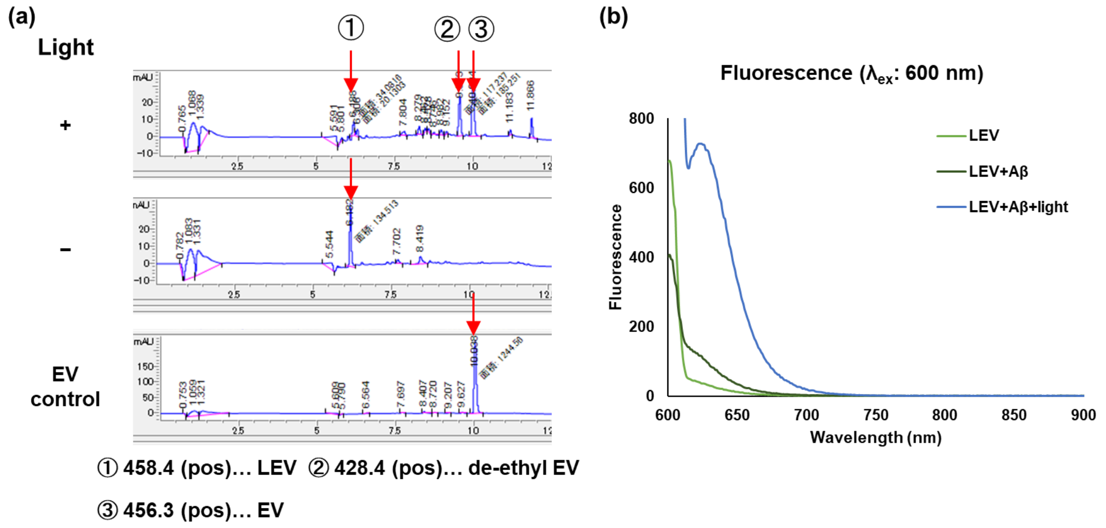


**Figure S16.** LC-MS analysis and fluorescence spectroscopy tracing the conversion of **LEV** to **EV**. (a) LC-MS analysis. A 20 µM **LEV** solution in 0.1 M PB (pH 7.4) was photoirradiated for 60 min and the resulting mixture was subjected to LC-MS analysis. (b) Fluorescence spectroscopy. To a 20 µM **LEV** solution in 0.1 M PB (pH 7.4), aggregated Aβ (20 µM) was added and photoirradiation was conducted for 60 min. After irradiation, fluorescence spectra were measured.

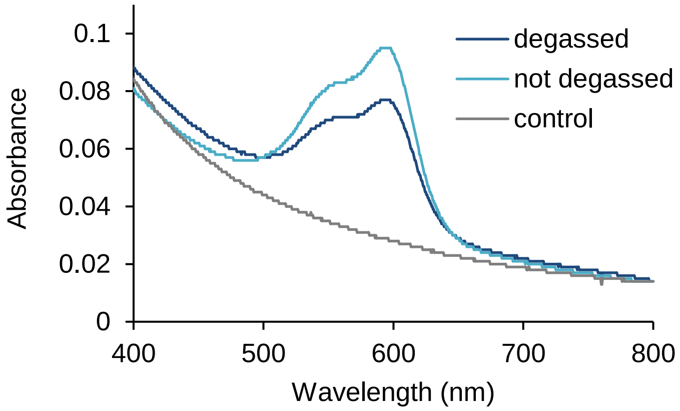


**Figure S17.** Involvement of $^3O_2$ in activation of **LEV** to **EV**. **LEV** (20 µM) was dissolved in a phosphate buffer solution (pH 7.4, degassed with freeze-pump-thaw method or not) and the solution was photoirradiated (λ = 595 nm, 10 mW) at 37 °C for 30 min. After photoirradiation, absorption spectra of the resulting solutions were measured. The control is the absorption spectrum of **LEV**.

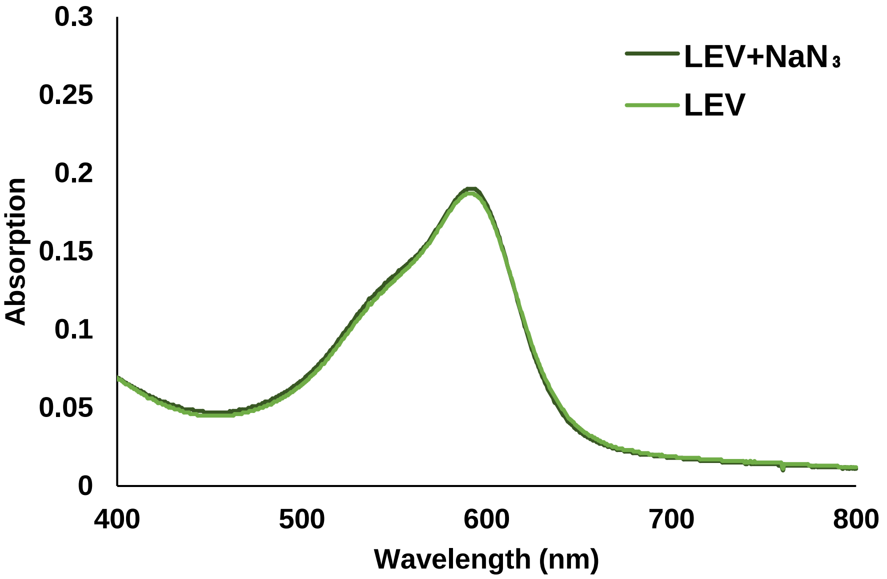


**Figure S18.** Effects of $NaN_3$ in activation of **LEV** to **EV** as a $^1O_2$ quenching reagent.[8] To a 20 μM **LEV** solution in 0.1 M PB (pH 7.4), $NaN_3$ (20 μM) was added, and the mixture was incubated at 37 °C for 60 min with photoirradiation (λ = 595 nm, 10 mW). After incubation, the absorption spectra of the solutions were measured.

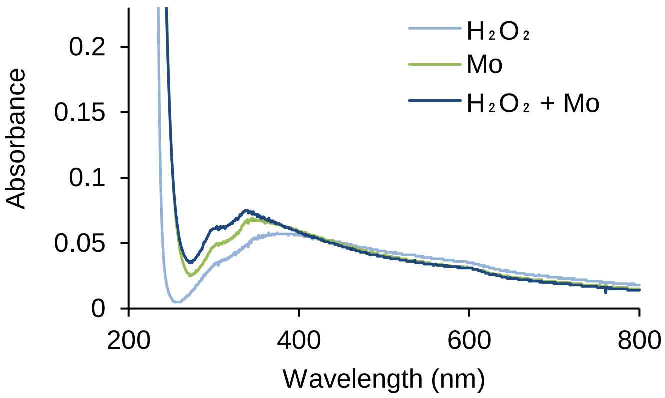


**Figure S19.** Photo-independent generation of $^1O_2$. To a 20 μM **LEV** solution in a PB (pH 7.4), $H_2O_2$ (2 mM) and $Na_2MoO_4$ (Mo; 2 mM)[9] were added and the solution was incubated at 37 °C for 60 min. The absorption spectra of the resulting solution were measured.

[8] C. S. Foote, T. T. Fujimoto, Y. C. Chang, *Tetrahedron Lett.* **1972**, *1*, 45.
[9] a) K. Boehme, H. D. Brauer, *Inorg. Chem.* **1992**, *31*, 3468. b) D. Pham, U. Basu, I. Pohorilets, C. M. St. Croix, S. C. Watkins, K. Koide, *Angew. Chem. Int. Ed.* **2020**, *59*, 17435.

**Calculated Bond Dissociation Energy (BDE)**

BDE = $\Delta_f H^0{}_{Radical} + \Delta_f H^0{}_{H} - \Delta_f H^0{}_{Molecule}$

$\Delta_f H^0$ = EE (Electronic Energy) + Thermal Correction for enthalpy

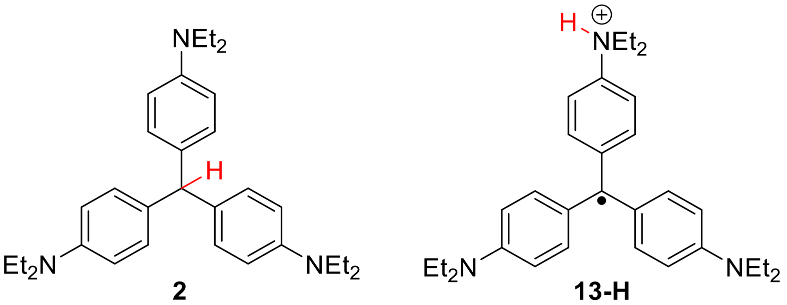


**2**

71.1 kcal/mol

**13-H**

75.2 kcal/mol

Calculation Results

(a) Hydrogen atom (H radical)

EE (Electronic Energy) = -0.500282 Hartree

Zero-point Energy Correction = 0 Hartree

Thermal Correction to Enthalpy (298.15 K) = 0.002360 Hartree

(b) **2**

(molecule)

EE (Electronic Energy) = -1371.493820 Hartree

Zero-point Energy Correction = 0.680161 Hartree

Thermal Correction to Enthalpy (298.15 K) = 0.717100 Hartree

(radical)

EE (Electronic Energy) = -1370.870017 Hartree

Zero-point Energy Correction = 0.667839 Hartree

Thermal Correction to Enthalpy (298.15 K) = 0.704469 Hartree

(c) **13-H**

(molecule)

EE (Electronic Energy) = -1371.316224 Hartree

Zero-point Energy Correction = 0.683069 Hartree

Thermal Correction to Enthalpy (298.15 K) = 0.719805 Hartree

(radical)

EE (Electronic Energy) = -1370.684798 Hartree

Zero-point Energy Correction = 0.669607 Hartree

Thermal Correction to Enthalpy (298.15 K) = 0.706136 Hartree

These results suggested that HAT process from **2** by **3***(T1) is thermodynamically feasible.

**Figure S20.** DFT Calculations for bond dissociation energies. Geometry optimization with the density functional theory (DFT) method was performed by the Gaussian 16 program package[10]. The ground-state geometry was optimized at the B3LYP/6-31G+(d) level with the conductor-like polarizable continuum model (CPCM) for the solvation effects of water. The geometry optimization for open shell species was performed at the UB3LYP/6-31G+(d) level. Those geometries having minimum energies were confirmed by frequency calculation. Parameters for thermal correction were obtained from the frequency calculation. Subsequent single point calculations for open shell species have been performed at the ROB3LYP/6-31G+(d) level with the CPCM for the solvation effects of water.

[10] Frisch, M. J., Trucks, G. W., Schlegel, H. B., Scuseria, G. E., Robb, M. A., Cheeseman, J. R., Scalmani, G., Barone, V., Petersson, G.A., Nakatsuji, H., Li, X., Caricato, M., Marenich, A. V., Bloino, J., Janesko, B. G., Gomperts, R., Mennucci, B., Hratchian, H. P., Ortiz, J. V., Izmaylov, A. F., Sonnemberg, J. L., Williams-Young, D., Ding, F., Lipparini, F., Egidi, F., Goings, J., Peng, B., Petrone, A., Henderson, T., Ranasinghe, D., Zakrzewski, V. G., Gao, J., Rega, N., Zheng, G., Liang, W., Hada, M., Ehara, M., Toyota, K., Fukuda, R., Hasegawa, J., Ishida, M., Nakajima, T., Honda, Y., Kitao, O., Nakai, H., Vreven, T., Throssell, K., Montgomery, Jr., J. A., Peralta, J. E., Ogliaro, F., Bearpark, M., Heyd, J. J., Brothers, E., Kudin, K. N., Staroverov, V. N., Keith, T. A., Kobayashi, R., Normand, J., Raghavachari, K., Rendell, A., Burant, J. C., Iyengar, S. S., Tomasi, J., Cossi, M., Millam, J. M., Klene, M., Adamo, C., Cammi, R., Ochterski, J. W., Martin, R. L., Morokuma, K., Farkas, O., Foresman, J. B., Fox, D. J. (2016) Gaussian 16, Revision A. 03; Gaussian, Inc.: Wallingford, CT.

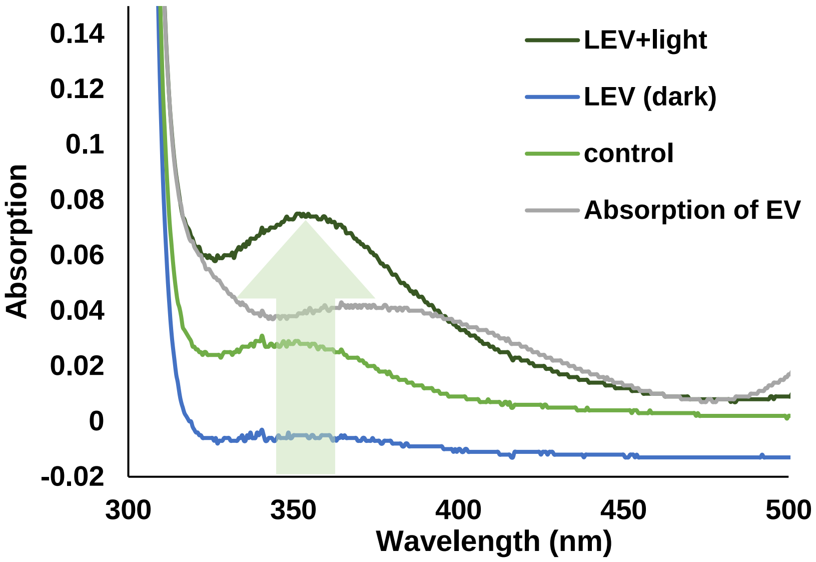


$H_2O_2$ oxidizes iodide anion ($I^-$) under acidic conditions to generate triiodide anion ($I_3^-$), which furnishes an absorption peak at 350 nm. The peak at 350 nm was detected by photoirradiation of **LEV**. This peak did not appear without photoirradiation. The result supports $H_2O_2$ production.

**Figure S21.** Iodometry detection of $H_2O_2$ generation in photoactivation of **LEV** to **EV**. A 100 µM **LEV** solution in 0.1 M phosphate buffer (pH 7.4) was photoirradiated (595 nm) at 37 °C for 60 min. After photoirradiation, potassium iodide (4000 eq.) and potassium hydrogen phthalate (1000 eq.) were added, and the mixture was incubated at r.t. for 30 min. After incubation, the absorption spectrum of the solution was measured. Control means no addition of **LEV**.

(a)

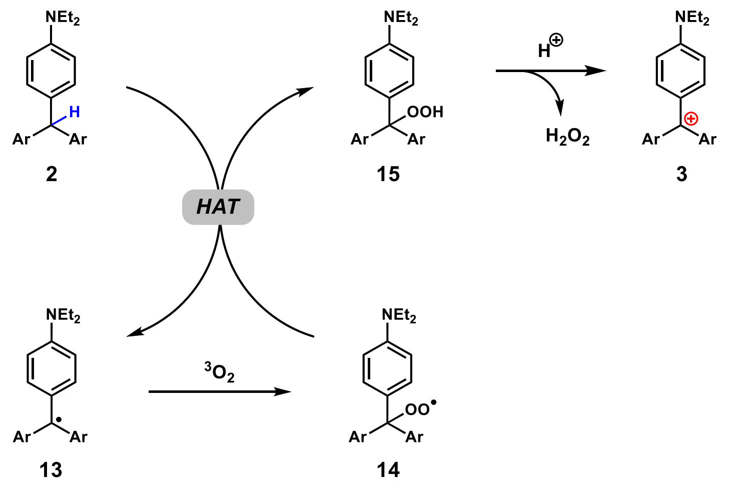


(b)

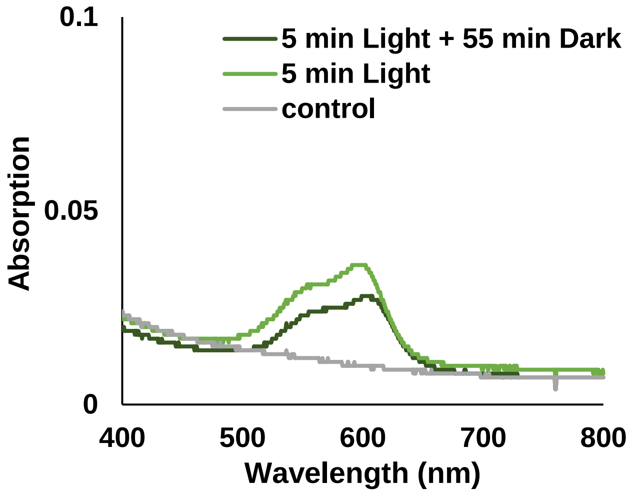


A possibility of radical chain mechanism was investigated. If peroxy radical species **14** directly abstracts the hydrogen atom from the methine C–H bond of **LEV**, photoirradiation would not be necessary for generating **EV** from **LEV** (Figure S22a). To assess if such a radical chain mechanism is operating, a **LEV** solution was photoirradiated for 5 min and then incubated for 55 min in the dark. As a result, increase in the absorption intensity at 600 nm corresponding to **EV** was not observed compared to the condition with photoirradiation for 5 min (Figure S21b). This result indicate that the radical chain mechanism is not a main pathway in the conversion from **LEV** to **EV**.

**Figure S22.** Possibility of radical chain mechanism. (a) Proposed reaction scheme. (b) ON/OFF experiment. A 20 μM solution of **LEV** in 0.1 M PB (pH 7.4) was photoirradiated (λ = 600 nm) for 5 min and then incubated for 55 min in the dark at 37 °C. The absorption spectrum of the solution was obtained (black line). The spectrum was compared to that of a solution of **LEV** photoirradiated for 5 min (green line) and **LEV** without photoirradiation (control, grey line).

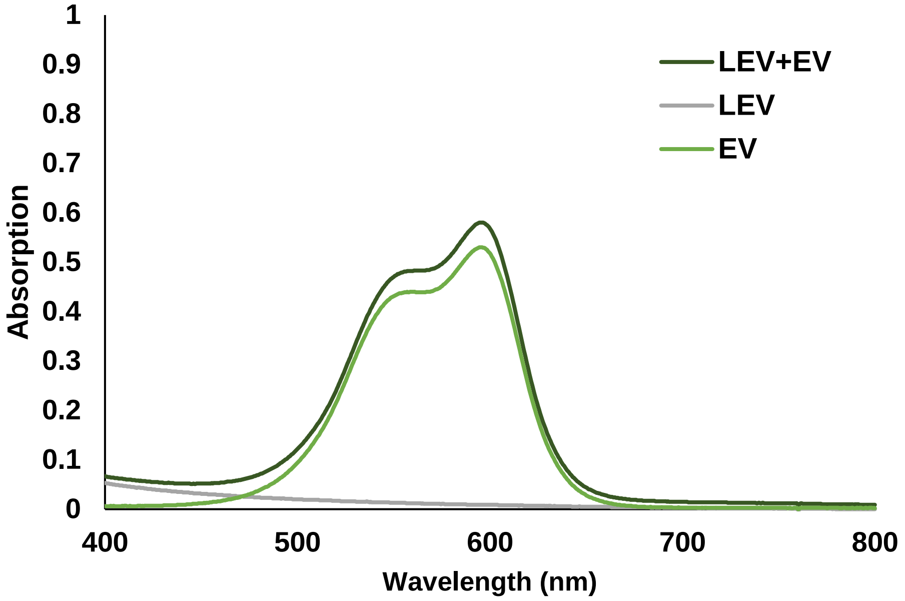


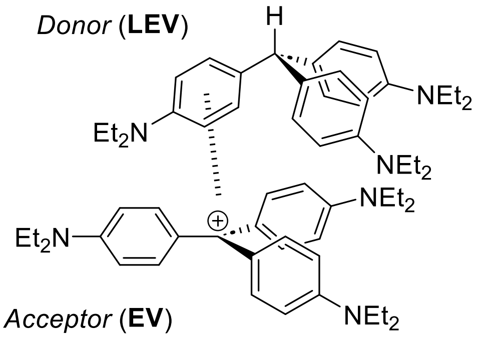


***EDA complex***

A possibility of EDA (electron donor-acceptor) complex formation between **EV** and **LEV** was investigated. A mixed solution of **LEV** and **EV** was prepared, and its absorption spectrum was measured. As a result, the mixed solution afforded almost the same spectrum as that of only **EV**. This result indicate that EDA complexation did not occur between **EV** and **LEV**.

**Figure S23.** Absorption spectrum of a mixed solution of **EV** and **LEV**. A mixture of **LEV** and **EV** (20 µM each) was dissolved in 0.1 M phosphate buffer (pH 7.4) and the absorption spectra of the solutions were measured.

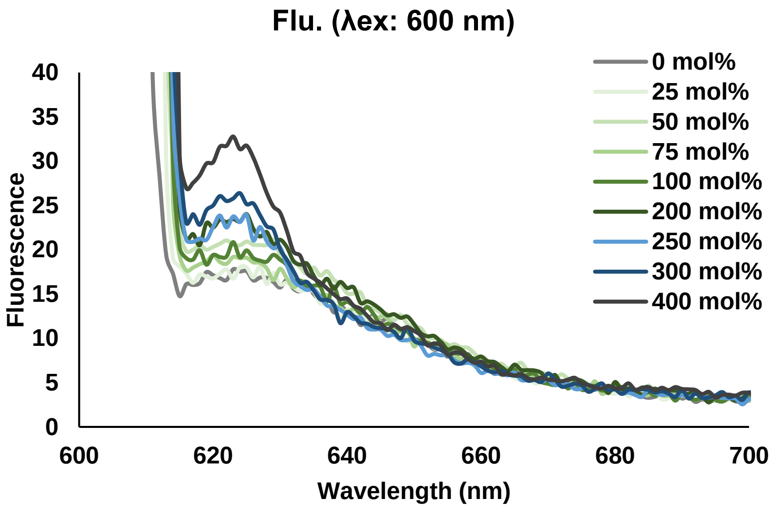


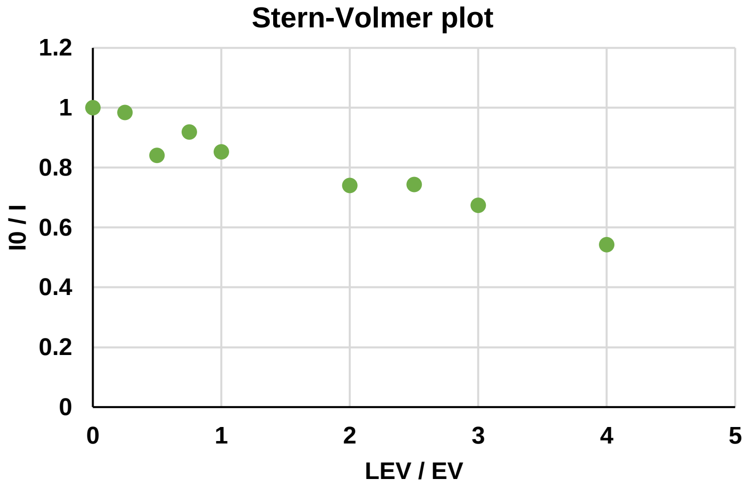


A possibility of exciplex formation between **EV** and **LEV** was investigated. The fluorescence quenching (Stern-Volmer) experiment of **EV** by the presence of variable concentrations of **LEV** was conducted. However, it did not afford a linear relationship with a positive slope. Therefore, exciplex formation or energy transfer did not occur between **EV** and **LEV**. Rather, a slight increase in fluorescence of **EV** was observed according to the addition of **LEV**. This is likely due to hydrophobic interaction between **EV** and **LEV**.

**Figure S24.** Fluorescence spectra of mixed solutions of **EV** and **LEV** and Stern-Volmer plot. Fluorescence spectra of mixed solutions of **EV** (20 μM) and **LEV** with variable concentrations in 0.1 M phosphate buffer (pH 7.4). "mol%" means the molar ratio of **LEV** to **EV**.

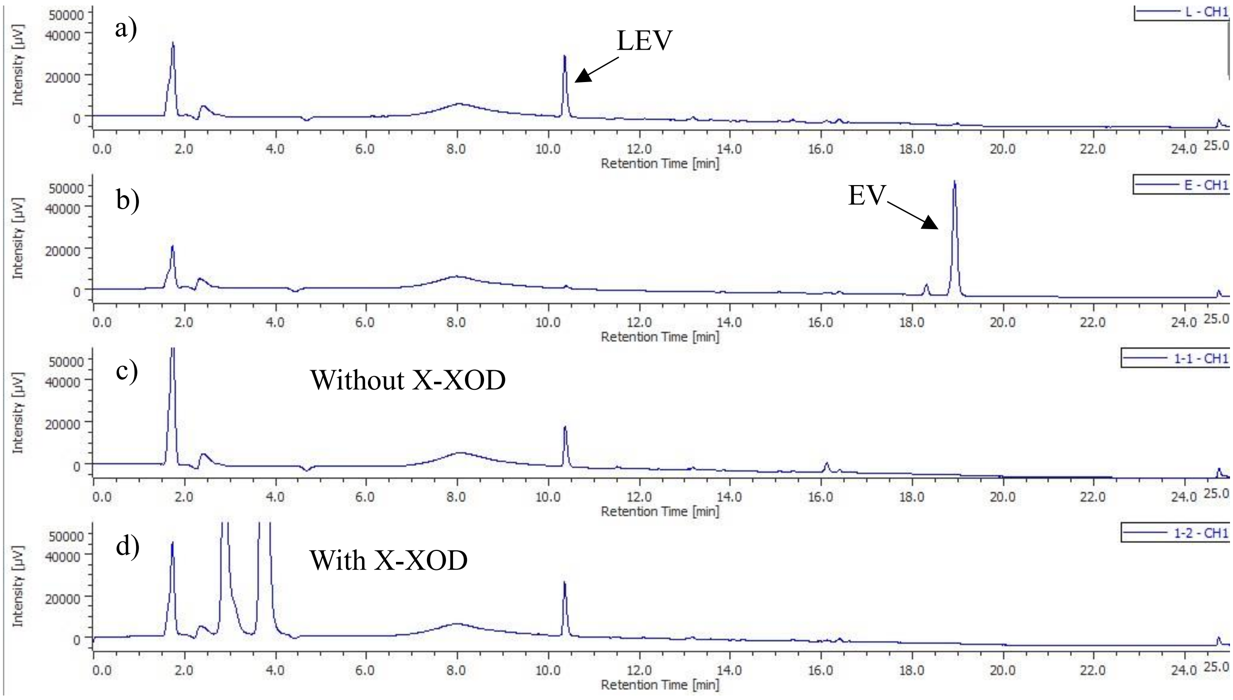


We utilized the xanthine (X)-xanthine oxidase (XOD) system (X-XOD), known for generating superoxide anion,[11] to verify the involvement of superoxide anion in the conversion from **LEV** to **EV**. We compared the amount of EV produced after 24 h with and without X-XOD. In the HPLC charts above, chart c represents the results without X-XOD, and chart d with X-XOD. As can be discerned when compared to control data, in both charts, there is almost complete retention of **LEV** with no production of **EV** after 24 h. This result suggests that superoxide anion does not play a role in the conversion of **LEV** to **EV**.

**Figure S25.** HPLC analysis assessing the involvement of superoxide anion in activation of **LEV** to **EV**. a) Authentic **LEV** sample. b) Authentic **EV** sample. c) A 0.18 mM **LEV** solution in 0.1 M PB (pH 7.4) was incubated for 24 h and the resulting mixture was subjected to HPLC analysis. d) A 0.18 mM **LEV**, 360 µM xanthine, and 45 mU/ml XOD solution in 0.1 M PB (pH 7.4) was incubated for 24 h and the resulting mixture was subjected to HPLC analysis.

[11] I. Fridovich, *J. Biol. Chem.* **1970**, *245*, 4053.

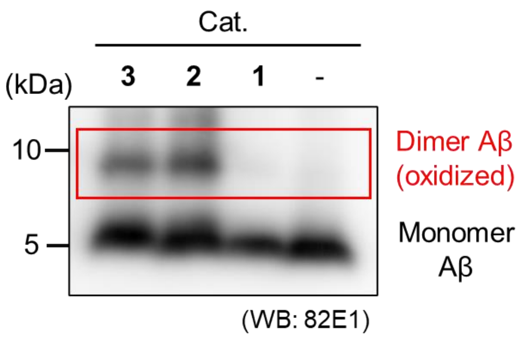


**Figure S26.** Photooxygenation reaction in the brain lysate (Western blotting). The brain excised from a 10-month-old *App* knock-in (*App*$^{NL\text{-}G\text{-}F/NL\text{-}G\text{-}F}$) mouse expressing human Arctic Aβs was homogenized in PBS (containing cOmplete EDTA+ (Roche) and PhosSTOP (Sigma)), and the suspensions were stored at −80 °C until use. The catalyst (final 50 μM) was added to the PBS-lysate suspension and the mixture was photoirradiated with LED (λ = 595 nm for **EV 3**, **LEV 2**, or **ABB 1**) or kept in the dark (–), at 37 °C for 3 h. After the reaction, the mixture was centrifuged (260,000 g, 20 min, 4 °C), and the supernatant was removed. The resulting precipitate was dissolved in DMSO. The samples were separated by SDS-PAGE using a 15% Tris-Tricine gel. Western blotting analysis was performed using an anti-Aβ antibody (82E1; IBL).

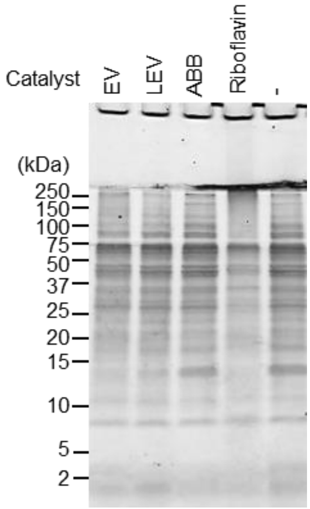


To evaluate Aβ amyloid selectivity, effects of the catalytic photooxygenation on off-target proteins in lysate were studied. The band pattern markedly changed from the control (without catalyst and photoiradiation, lane 5) after photooxygenation using a non-selective catalyst, Riboflavin (lane 4). The band pattern with **EV** (lane 1) and **LEV** (lane 2) was generally similar to the control, except that some bands were faint. Using **ABB**, the band pattern was almost the same as the control. Using **ABB**, however, photooxygenation of Aβ amyloid proceeded only to a very small extent (see Figure S26).

**Figure S27.** Off-target photooxygenation of proteins in lysate. The supernatant fraction of the lysate after the reaction of Figure S26 was analyzed with SDS-PAGE and CBB staining.

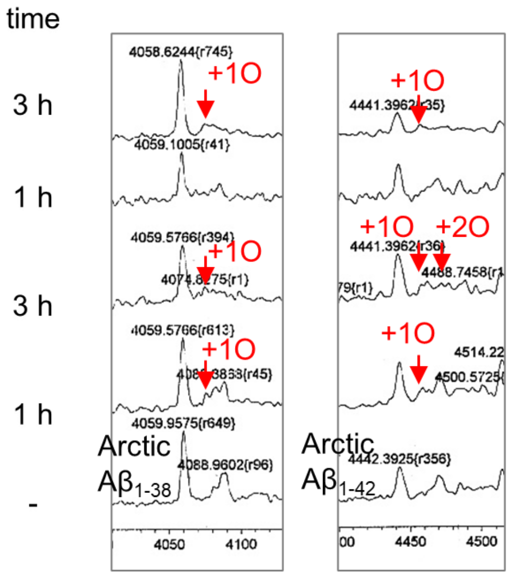


**Figure S28.** MALDI-TOF MS charts for photooxygenation of the brain lysate. To a brain lysate of *App*$^{NL\text{-}G\text{-}F/NL\text{-}G\text{-}F}$ mouse (10-month-old), 50 µM **EV** was added. The mixture was photoirradiated with 595 nm light at 37 °C for 3 h. The resulting samples were analyzed by MALDI-TOF MS. The left and right figures show the time course of oxygenation of A$\beta_{1\text{-}38}$ and A$\beta_{1\text{-}42}$, respectively.

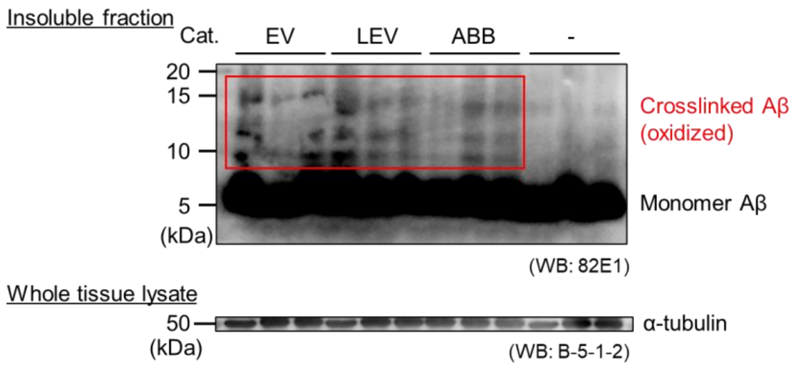


**Figure S29.** *In vivo* photooxygenation reaction for 2 months (detection of crosslinked bands). To *App*$^{NL\text{-}G\text{-}F/NL\text{-}G\text{-}F}$ mice ($n$ = 3 experiments, each group), a catalyst (1 mM, 200 µL) was intravenously injected. After certain time (**EV** and **ABB**: 60 min, **LEV**: 5 min), heads of the mice were irradiated with 595 nm light for 10 min. The treatment was repeated 3 times per week over 2 months. After the treatments, the brain was extracted. The lysate of the brain was fractionated and analyzed with Western blotting by anti-Aβ antibodies (Aβ: 82E1, α-tubulin: B-5-1-2). "—" means no treatments.

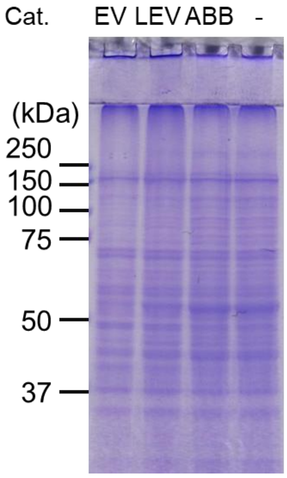


**Figure S30.** Human brain lysate SDS-PAGE. The photooxygenated samples of Figure 6C and 6D were analyzed with SDS-PAGE and CBB staining. Although there was some loss of proteins in some bands compared to the control, the overall protein level was generally not so much changed.

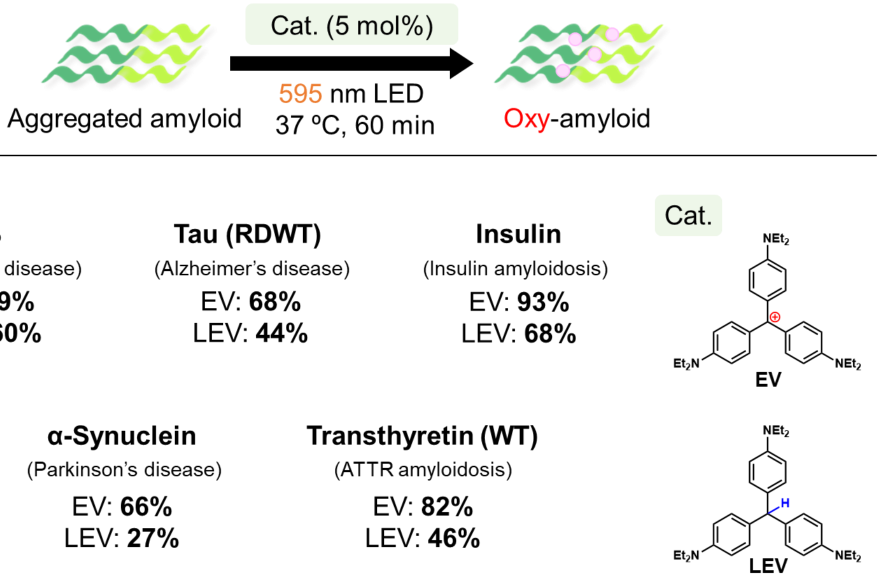


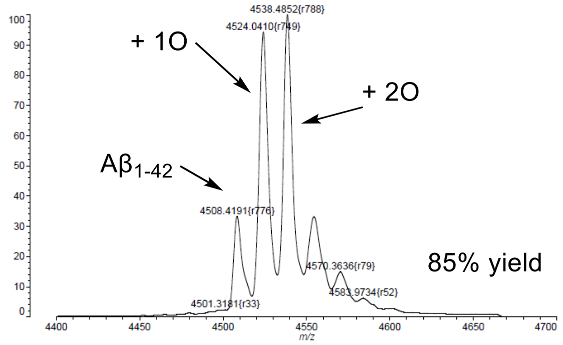


MALDI-TOF MS chart for photooxygenation of Aβ using EV

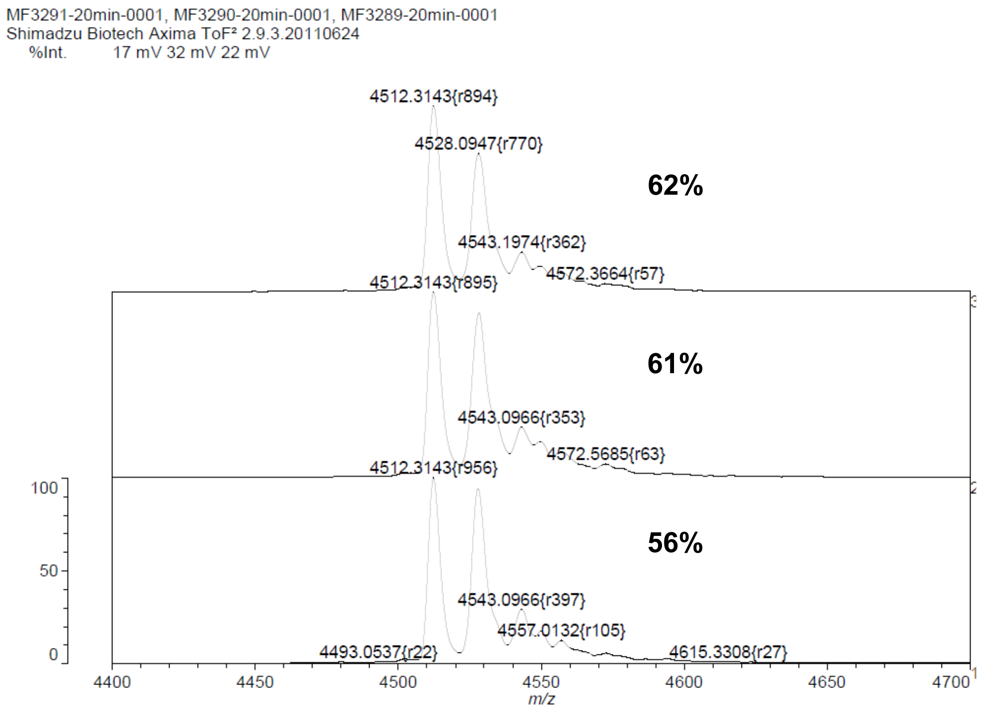


MALDI-TOF MS chart for photooxygenation of Aβ using LEV

**Figure S31.** Scope of amyloids in catalytic photooxygenation. To a solution of amyloid, 5 mol% **EV** or **LEV** was added, and the solution was photoirradiated at 595 nm for 60 min. After photoirradiation, oxygenation yield was measured using MALDI-TOF MS. Tau (RDWT) indicates a model tau (recombinant repeat domain of wild type tau). α-Synuclein and transthyretin were analyzed after digestion with trypsin.

a)

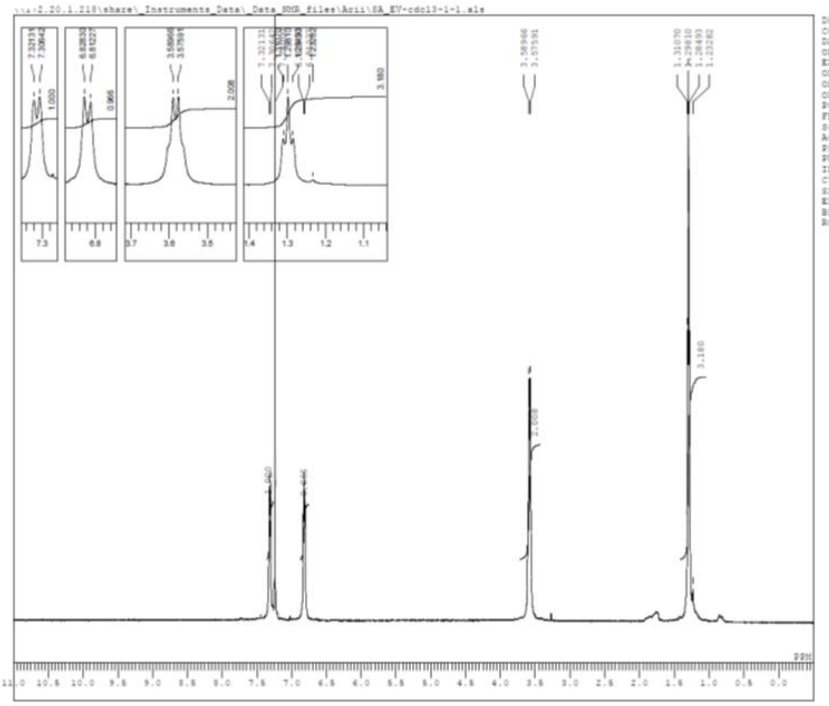

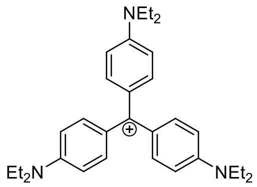


**3**: EV
($CDCl_3$)

b)

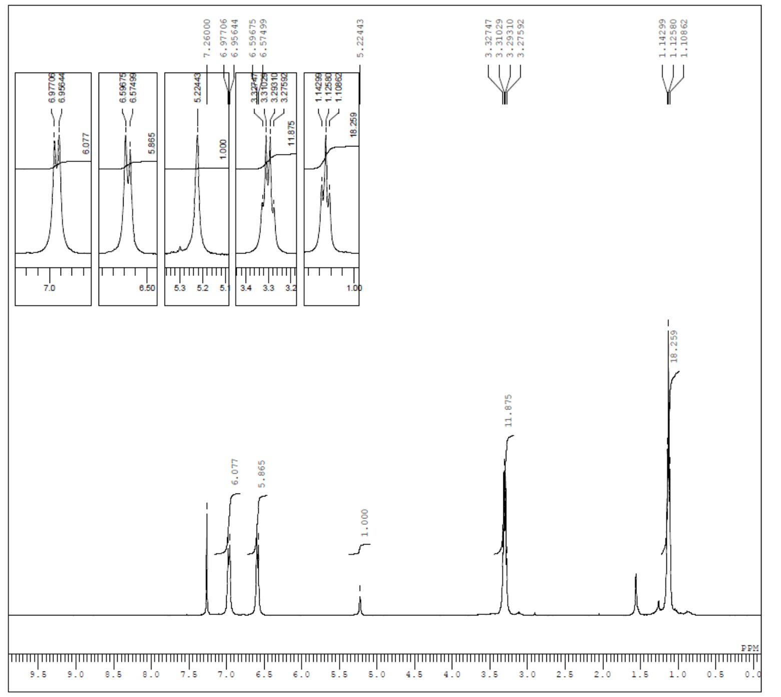

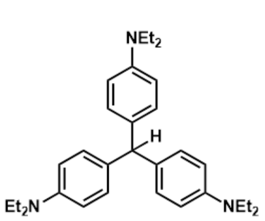


**LEV (2)**

c)

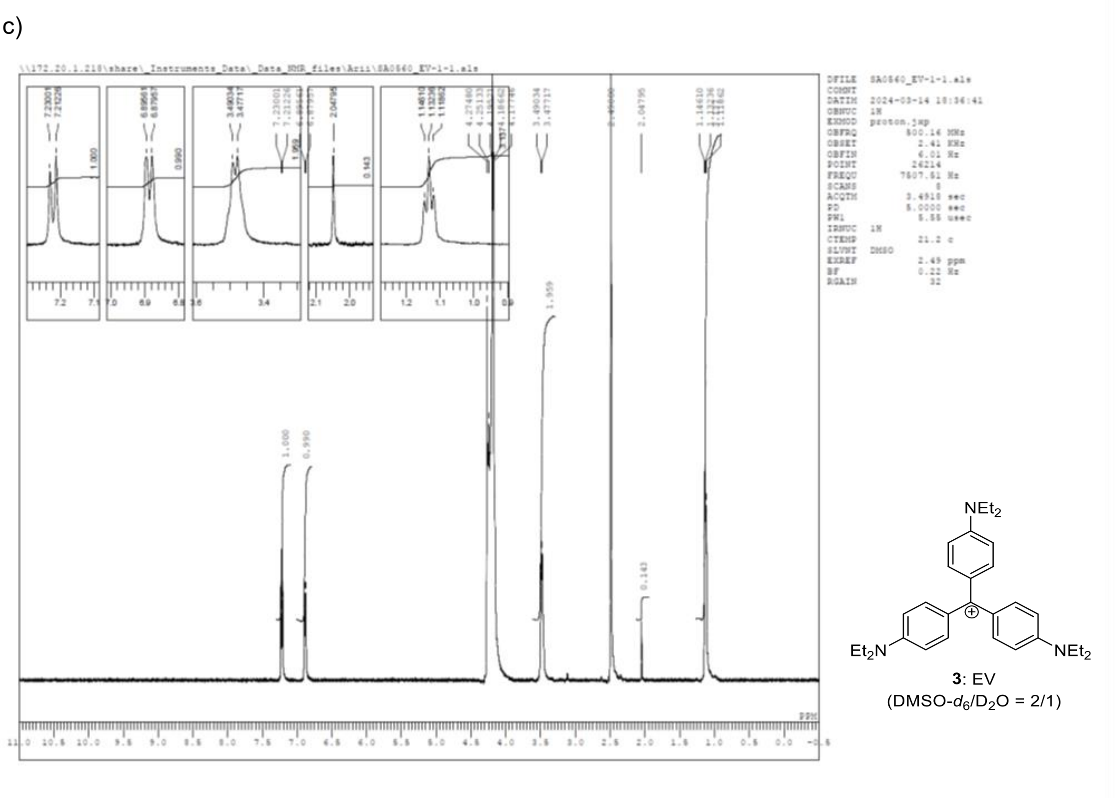


d)

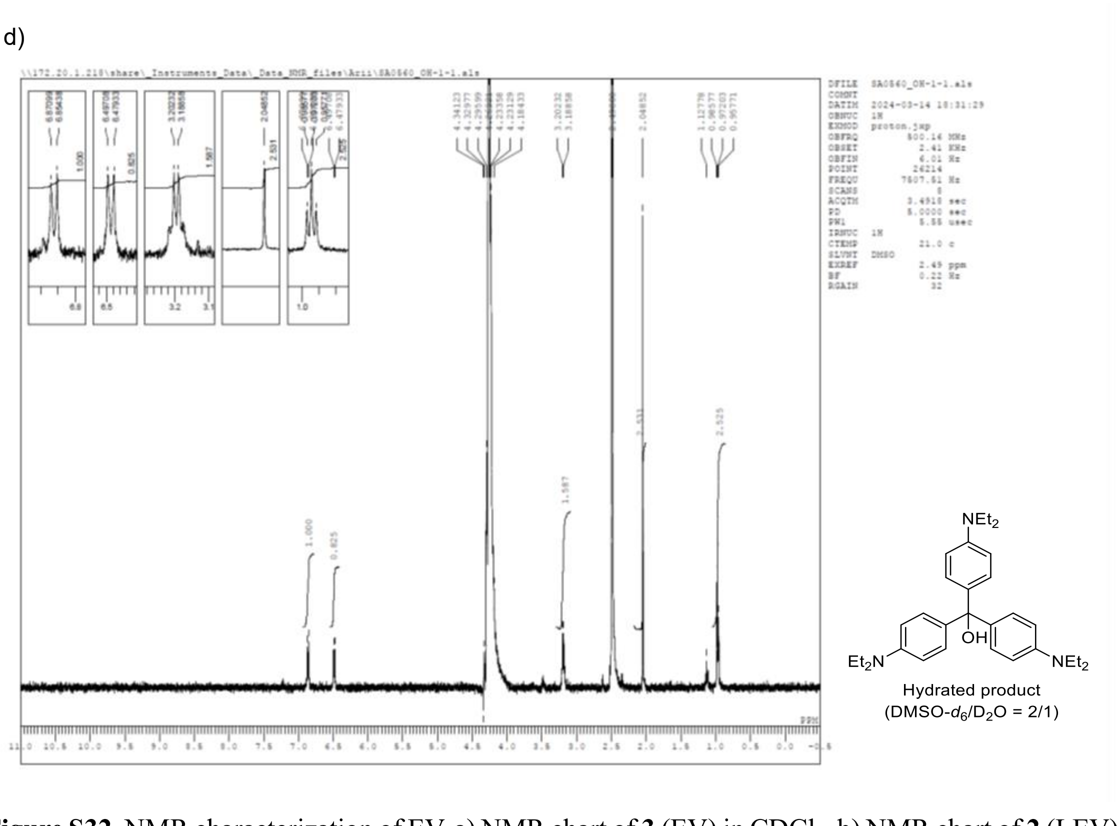


**Figure S32.** NMR characterization of EV. a) NMR chart of **3** (EV) in $CDCl_3$. b) NMR chart of **2** (LEV) in $CDCl_3$. c) NMR chart of **3** (EV) in DMSO-$d_6$/$D_2O$. d) NMR chart of hydrated product in DMSO-

$d_6$/$D_2O$. LEV and EV can be differentiated in $CDCl_3$ (NMR charts a and b). No evidence of the trityl alcohol form resulting from hydration was detected, assuring the stability of EV in aqueous environments (NMR charts c and d).

**Table S1.** Standard nuclear orientation of the optimized $S_0$ geometry of **3** with Aβ calculated at the M06-2X/6-31G(d) level of theory.

| Atom | Element symbol | *x* (Å) | *y* (Å) | *z* (Å) |
|---|---|---|---|---|
| 1 | C | 0.000000 | 0.000000 | 0.000000 |
| 2 | C | 0.000000 | 1.439066 | 0.000000 |
| 3 | C | -1.029621 | 2.183940 | 0.627228 |
| 4 | C | -1.030105 | 3.556875 | 0.644638 |
| 5 | C | 0.000000 | 4.301093 | 0.000000 |
| 6 | C | 1.029621 | 2.183940 | -0.627228 |
| 7 | C | 1.030105 | 3.556875 | -0.644638 |
| 8 | N | 0.000000 | 5.654122 | 0.000000 |
| 9 | C | -1.115577 | 6.428658 | 0.540569 |
| 10 | C | 1.115577 | 6.428658 | -0.540569 |
| 11 | C | -1.246268 | -0.719533 | 0.000000 |
| 12 | C | -1.376537 | -1.983648 | 0.627228 |
| 13 | C | -2.565292 | -2.670535 | 0.644638 |
| 14 | C | -3.724856 | -2.150547 | 0.000000 |
| 15 | C | -2.406158 | -0.200292 | -0.627228 |
| 16 | C | -3.595397 | -0.886340 | -0.644638 |
| 17 | N | -4.896613 | -2.827061 | 0.000000 |
| 18 | C | -5.009593 | -4.180447 | 0.540569 |
| 19 | C | -6.125169 | -2.248211 | -0.540569 |
| 20 | C | 1.246268 | -0.719533 | 0.000000 |
| 21 | C | 2.406158 | -0.200292 | 0.627228 |
| 22 | C | 3.595397 | -0.886340 | 0.644638 |
| 23 | C | 3.724856 | -2.150547 | 0.000000 |
| 24 | C | 1.376537 | -1.983648 | -0.627228 |
| 25 | C | 2.565292 | -2.670535 | -0.644638 |
| 26 | N | 4.896613 | -2.827061 | 0.000000 |
| 27 | C | 6.125169 | -2.248211 | 0.540569 |
| 28 | C | 5.009593 | -4.180447 | -0.540569 |
| 29 | H | -1.811060 | 1.652656 | 1.161974 |
| 30 | H | -1.811289 | 4.069231 | 1.191528 |
| 31 | H | 1.811060 | 1.652656 | -1.161974 |
| 32 | H | 1.811289 | 4.069231 | -1.191528 |
| 33 | H | -1.140307 | 7.376665 | -0.005462 |
| 34 | H | -2.053752 | 5.918761 | 0.306555 |
| 35 | C | -0.979258 | 6.686286 | 2.038982 |
| 36 | H | 1.140307 | 7.376665 | 0.005462 |
| 37 | H | 2.053752 | 5.918761 | -0.306555 |
| 38 | C | 0.979258 | 6.686286 | -2.038982 |
| 39 | H | -1.821246 | 7.280864 | 2.403071 |
| 40 | H | -0.056903 | 7.234427 | 2.252201 |
| 41 | H | -0.948953 | 5.747223 | 2.598762 |
| 42 | H | 1.821246 | 7.280864 | -2.403071 |

| | | | | |
|---|---|---|---|---|
| 43 | H | 0.056903 | 7.234427 | -2.252201 |
| 44 | H | 0.948953 | 5.747223 | -2.598762 |
| 45 | H | -0.525712 | -2.394752 | 1.161974 |
| 46 | H | -2.618413 | -3.603238 | 1.191528 |
| 47 | H | -2.336772 | 0.742096 | -1.161974 |
| 48 | H | -4.429702 | -0.465993 | -1.191528 |
| 49 | H | -5.818226 | -4.675867 | -0.005462 |
| 50 | H | -4.098922 | -4.737982 | 0.306555 |
| 51 | C | -5.300865 | -4.191206 | 2.038982 |
| 52 | H | -6.958533 | -2.700798 | 0.005462 |
| 53 | H | -6.152674 | -1.180779 | -0.306555 |
| 54 | C | -6.280123 | -2.495080 | -2.038982 |
| 55 | H | -5.394790 | -5.217677 | 2.403071 |
| 56 | H | -6.236746 | -3.666493 | 2.252201 |
| 57 | H | -4.502764 | -3.695429 | 2.598762 |
| 58 | H | -7.216036 | -2.063187 | -2.403071 |
| 59 | H | -6.293649 | -3.567935 | -2.252201 |
| 60 | H | -5.451717 | -2.051794 | -2.598762 |
| 61 | H | 2.336772 | 0.742096 | 1.161974 |
| 62 | H | 4.429702 | -0.465993 | 1.191528 |
| 63 | H | 0.525712 | -2.394752 | -1.161974 |
| 64 | H | 2.618413 | -3.603238 | -1.191528 |
| 65 | H | 6.958533 | -2.700798 | -0.005462 |
| 66 | H | 6.152674 | -1.180779 | 0.306555 |
| 67 | C | 6.280123 | -2.495080 | 2.038982 |
| 68 | H | 5.818226 | -4.675867 | 0.005462 |
| 69 | H | 4.098922 | -4.737982 | -0.306555 |
| 70 | C | 5.300865 | -4.191206 | -2.038982 |
| 71 | H | 7.216036 | -2.063187 | 2.403071 |
| 72 | H | 6.293649 | -3.567935 | 2.252201 |
| 73 | H | 5.451717 | -2.051794 | 2.598762 |
| 74 | H | 5.394790 | -5.217677 | -2.403071 |
| 75 | H | 6.236746 | -3.666493 | -2.252201 |
| 76 | H | 4.502764 | -3.695429 | -2.598762 |

**Table S2.** Standard nuclear orientation of the optimized $S_1$ geometry of **3** with Aβ calculated at the TDA-M06-2X/6-31G(d) level of theory.

| Atom | Element symbol | *x* (Å) | *y* (Å) | *z* (Å) |
|---|---|---|---|---|
| 1 | C | 0.000000 | 0.000000 | 0.000000 |
| 2 | C | 0.000000 | 0.000000 | 1.454713 |
| 3 | C | 1.199777 | 0.081664 | 2.205473 |
| 4 | C | 1.210625 | 0.083262 | 3.580488 |
| 5 | C | 0.000000 | 0.000000 | 4.324765 |
| 6 | C | -1.199777 | -0.081664 | 2.205473 |

| | | | | |
|---|---|---|---|---|
| 7 | C | -1.210625 | -0.083262 | 3.580488 |
| 8 | N | 0.000000 | 0.000000 | 5.683475 |
| 9 | C | 1.237995 | -0.041826 | 6.456787 |
| 10 | C | -1.237995 | 0.041826 | 6.456787 |
| 11 | C | 0.000000 | -1.259819 | -0.727357 |
| 12 | C | 0.559103 | -1.377657 | -2.024774 |
| 13 | C | 0.564945 | -2.563815 | -2.720317 |
| 14 | C | 0.000000 | -3.745357 | -2.162383 |
| 15 | C | -0.559103 | -2.442335 | -0.180699 |
| 16 | C | -0.564945 | -3.637771 | -0.860170 |
| 17 | N | 0.000000 | -4.922034 | -2.841738 |
| 18 | C | 0.461555 | -5.016992 | -4.223889 |
| 19 | C | -0.461555 | -6.166491 | -2.232898 |
| 20 | C | 0.000000 | 1.259819 | -0.727357 |
| 21 | C | 0.559103 | 2.442335 | -0.180699 |
| 22 | C | 0.564945 | 3.637771 | -0.860170 |
| 23 | C | 0.000000 | 3.745357 | -2.162383 |
| 24 | C | -0.559103 | 1.377657 | -2.024774 |
| 25 | C | -0.564945 | 2.563815 | -2.720317 |
| 26 | N | 0.000000 | 4.922034 | -2.841738 |
| 27 | C | 0.461555 | 6.166491 | -2.232898 |
| 28 | C | -0.461555 | 5.016992 | -4.223889 |
| 29 | H | 2.144982 | 0.168793 | 1.679413 |
| 30 | H | 2.160068 | 0.178053 | 4.092639 |
| 31 | H | -2.144982 | -0.168793 | 1.679413 |
| 32 | H | -2.160068 | -0.178053 | 4.092639 |
| 33 | H | 1.004354 | -0.530337 | 7.407642 |
| 34 | H | 1.957589 | -0.688516 | 5.948482 |
| 35 | C | 1.818062 | 1.348872 | 6.707047 |
| 36 | H | -1.004354 | 0.530337 | 7.407642 |
| 37 | H | -1.957589 | 0.688516 | 5.948482 |
| 38 | C | -1.818062 | -1.348872 | 6.707047 |
| 39 | H | 2.733082 | 1.278245 | 7.300795 |
| 40 | H | 1.103132 | 1.971269 | 7.252873 |
| 41 | H | 2.051582 | 1.851297 | 5.764297 |
| 42 | H | -2.733082 | -1.278245 | 7.300795 |
| 43 | H | -1.103132 | -1.971269 | 7.252873 |
| 44 | H | -2.051582 | -1.851297 | 5.764297 |
| 45 | H | 1.020426 | -0.507292 | -2.480172 |
| 46 | H | 1.034989 | -2.592174 | -3.695500 |
| 47 | H | -1.020426 | -2.401538 | 0.800759 |
| 48 | H | -1.034989 | -4.496484 | -0.397139 |
| 49 | H | -0.079702 | -5.848718 | -4.685006 |
| 50 | H | 0.160438 | -4.117072 | -4.765986 |
| 51 | C | 1.968065 | -5.249114 | -4.322361 |
| 52 | H | 0.079702 | -6.981693 | -2.722636 |

| | | | | |
|---|---|---|---|---|
| 53 | H | -0.160438 | -6.186001 | -1.182496 |
| 54 | C | -1.968065 | -6.367832 | -2.384686 |
| 55 | H | 2.272874 | -5.330493 | -5.368905 |
| 56 | H | 2.248839 | -6.174491 | -3.811213 |
| 57 | H | 2.522001 | -4.427272 | -3.860335 |
| 58 | H | -2.272874 | -7.314854 | -1.931890 |
| 59 | H | -2.248839 | -6.387853 | -3.441660 |
| 60 | H | -2.522001 | -5.556784 | -1.903962 |
| 61 | H | 1.020426 | 2.401538 | 0.800759 |
| 62 | H | 1.034989 | 4.496484 | -0.397139 |
| 63 | H | -1.020426 | 0.507292 | -2.480172 |
| 64 | H | -1.034989 | 2.592174 | -3.695500 |
| 65 | H | -0.079702 | 6.981693 | -2.722636 |
| 66 | H | 0.160438 | 6.186001 | -1.182496 |
| 67 | C | 1.968065 | 6.367832 | -2.384686 |
| 68 | H | 0.079702 | 5.848718 | -4.685006 |
| 69 | H | -0.160438 | 4.117072 | -4.765986 |
| 70 | C | -1.968065 | 5.249114 | -4.322361 |
| 71 | H | 2.272874 | 7.314854 | -1.931890 |
| 72 | H | 2.248839 | 6.387853 | -3.441660 |
| 73 | H | 2.522001 | 5.556784 | -1.903962 |
| 74 | H | -2.272874 | 5.330493 | -5.368905 |
| 75 | H | -2.248839 | 6.174491 | -3.811213 |
| 76 | H | -2.522001 | 4.427272 | -3.860335 |

**Table S3.** Standard nuclear orientation of the optimized $S_0$ geometry of **3** without Aβ calculated at the M06-2X/6-31G(d) level of theory.

| Atom | Element symbol | *x* (Å) | *y* (Å) | *z* (Å) |
|---|---|---|---|---|
| 1 | C | 0.000000 | 0.000000 | 0.000000 |
| 2 | C | 0.000000 | 1.444756 | 0.000000 |
| 3 | C | -1.032287 | 2.195847 | 0.627414 |
| 4 | C | -1.032165 | 3.574825 | 0.644312 |
| 5 | C | 0.000000 | 4.322537 | 0.000000 |
| 6 | C | 1.032287 | 2.195847 | -0.627414 |
| 7 | C | 1.032165 | 3.574825 | -0.644312 |
| 8 | N | 0.000000 | 5.684837 | 0.000000 |
| 9 | C | -1.117012 | 6.467454 | 0.549919 |
| 10 | C | 1.117012 | 6.467454 | -0.549919 |
| 11 | C | -1.251195 | -0.722378 | 0.000000 |
| 12 | C | -1.385516 | -1.991910 | 0.627414 |
| 13 | C | -2.579806 | -2.681294 | 0.644312 |
| 14 | C | -3.743427 | -2.161268 | 0.000000 |
| 15 | C | -2.417803 | -0.203937 | -0.627414 |
| 16 | C | -3.611972 | -0.893531 | -0.644312 |

| | | | | |
|---|---|---|---|---|
| 17 | N | -4.923214 | -2.842419 | 0.000000 |
| 18 | C | -5.042474 | -4.201087 | 0.549919 |
| 19 | C | -6.159485 | -2.266367 | -0.549919 |
| 20 | C | 1.251195 | -0.722378 | 0.000000 |
| 21 | C | 2.417803 | -0.203937 | 0.627414 |
| 22 | C | 3.611972 | -0.893531 | 0.644312 |
| 23 | C | 3.743427 | -2.161268 | 0.000000 |
| 24 | C | 1.385516 | -1.991910 | -0.627414 |
| 25 | C | 2.579806 | -2.681294 | -0.644312 |
| 26 | N | 4.923214 | -2.842419 | 0.000000 |
| 27 | C | 6.159485 | -2.266367 | 0.549919 |
| 28 | C | 5.042474 | -4.201087 | -0.549919 |
| 29 | H | -1.816645 | 1.669287 | 1.162533 |
| 30 | H | -1.817528 | 4.084901 | 1.188945 |
| 31 | H | 1.816645 | 1.669287 | -1.162533 |
| 32 | H | 1.817528 | 4.084901 | -1.188945 |
| 33 | H | -1.151519 | 7.405473 | -0.012485 |
| 34 | H | -2.055245 | 5.945866 | 0.342983 |
| 35 | C | -0.965016 | 6.758223 | 2.047107 |
| 36 | H | 1.151519 | 7.405473 | 0.012485 |
| 37 | H | 2.055245 | 5.945866 | -0.342983 |
| 38 | C | 0.965016 | 6.758223 | -2.047107 |
| 39 | H | -1.806573 | 7.367016 | 2.394435 |
| 40 | H | -0.039111 | 7.309046 | 2.243676 |
| 41 | H | -0.943664 | 5.832223 | 2.630510 |
| 42 | H | 1.806573 | 7.367016 | -2.394435 |
| 43 | H | 0.039111 | 7.309046 | -2.243676 |
| 44 | H | 0.943664 | 5.832223 | -2.630510 |
| 45 | H | -0.537322 | -2.407904 | 1.162533 |
| 46 | H | -2.628864 | -3.616476 | 1.188945 |
| 47 | H | -2.353967 | 0.738618 | -1.162533 |
| 48 | H | -4.446392 | -0.468425 | -1.188945 |
| 49 | H | -5.837568 | -4.699981 | -0.012485 |
| 50 | H | -4.121649 | -4.752827 | 0.342983 |
| 51 | C | -5.370285 | -4.214840 | 2.047107 |
| 52 | H | -6.989087 | -2.705491 | 0.012485 |
| 53 | H | -6.176894 | -1.193039 | -0.342983 |
| 54 | C | -6.335301 | -2.543383 | -2.047107 |
| 55 | H | -5.476736 | -5.248046 | 2.394435 |
| 56 | H | -6.310264 | -3.688394 | 2.243676 |
| 57 | H | -4.579022 | -3.733348 | 2.630510 |
| 58 | H | -7.283309 | -2.118970 | -2.394435 |
| 59 | H | -6.349375 | -3.620652 | -2.243676 |
| 60 | H | -5.522685 | -2.098875 | -2.630510 |
| 61 | H | 2.353967 | 0.738618 | 1.162533 |
| 62 | H | 4.446392 | -0.468425 | 1.188945 |

| | | | | |
|---|---|---|---|---|
| 63 | H | 0.537322 | -2.407904 | -1.162533 |
| 64 | H | 2.628864 | -3.616476 | -1.188945 |
| 65 | H | 6.989087 | -2.705491 | -0.012485 |
| 66 | H | 6.176894 | -1.193039 | 0.342983 |
| 67 | C | 6.335301 | -2.543383 | 2.047107 |
| 68 | H | 5.837568 | -4.699981 | 0.012485 |
| 69 | H | 4.121649 | -4.752827 | -0.342983 |
| 70 | C | 5.370285 | -4.214840 | -2.047107 |
| 71 | H | 7.283309 | -2.118970 | 2.394435 |
| 72 | H | 6.349375 | -3.620652 | 2.243676 |
| 73 | H | 5.522685 | -2.098875 | 2.630510 |
| 74 | H | 5.476736 | -5.248046 | -2.394435 |
| 75 | H | 6.310264 | -3.688394 | -2.243676 |
| 76 | H | 4.579022 | -3.733348 | -2.630510 |

**Table S4.** Standard nuclear orientation of the optimized $S_1$ geometry of **3** without Aβ calculated at the TDA-M06-2X/6-31G(d) level of theory.

| Atom | Element symbol | *x* (Å) | *y* (Å) | *z* (Å) |
|---|---|---|---|---|
| 1 | C | 0.000000 | 0.000000 | 0.000000 |
| 2 | C | 0.000000 | 0.000000 | 1.461100 |
| 3 | C | 1.200840 | 0.082588 | 2.215230 |
| 4 | C | 1.210281 | 0.085502 | 3.599204 |
| 5 | C | 0.000000 | 0.000000 | 4.343396 |
| 6 | C | -1.200840 | -0.082588 | 2.215230 |
| 7 | C | -1.210281 | -0.085502 | 3.599204 |
| 8 | N | 0.000000 | 0.000000 | 5.717518 |
| 9 | C | 1.244094 | -0.033494 | 6.496741 |
| 10 | C | -1.244094 | 0.033494 | 6.496741 |
| 11 | C | 0.000000 | -1.265349 | -0.730550 |
| 12 | C | 0.542674 | -1.381244 | -2.038076 |
| 13 | C | 0.549027 | -2.576017 | -2.736616 |
| 14 | C | 0.000000 | -3.761491 | -2.171698 |
| 15 | C | -0.542674 | -2.455647 | -0.177154 |
| 16 | C | -0.549027 | -3.657987 | -0.862589 |
| 17 | N | 0.000000 | -4.951516 | -2.858759 |
| 18 | C | 0.452518 | -5.046662 | -4.252407 |
| 19 | C | -0.452518 | -6.206024 | -2.244334 |
| 20 | C | 0.000000 | 1.265349 | -0.730550 |
| 21 | C | 0.542674 | 2.455647 | -0.177154 |
| 22 | C | 0.549027 | 3.657987 | -0.862589 |
| 23 | C | 0.000000 | 3.761491 | -2.171698 |
| 24 | C | -0.542674 | 1.381244 | -2.038076 |
| 25 | C | -0.549027 | 2.576017 | -2.736616 |
| 26 | N | 0.000000 | 4.951516 | -2.858759 |

| | | | | |
|---|---|---|---|---|
| 27 | C | 0.452518 | 6.206024 | -2.244334 |
| 28 | C | -0.452518 | 5.046662 | -4.252407 |
| 29 | H | 2.149387 | 0.170794 | 1.694706 |
| 30 | H | 2.162302 | 0.179263 | 4.108267 |
| 31 | H | -2.149387 | -0.170794 | 1.694706 |
| 32 | H | -2.162302 | -0.179263 | 4.108267 |
| 33 | H | 1.013122 | -0.539341 | 7.438874 |
| 34 | H | 1.975607 | -0.656284 | 5.975742 |
| 35 | C | 1.809226 | 1.364821 | 6.777221 |
| 36 | H | -1.013122 | 0.539341 | 7.438874 |
| 37 | H | -1.975607 | 0.656284 | 5.975742 |
| 38 | C | -1.809226 | -1.364821 | 6.777221 |
| 39 | H | 2.716842 | 1.279965 | 7.383793 |
| 40 | H | 1.083820 | 1.973767 | 7.326586 |
| 41 | H | 2.059989 | 1.883333 | 5.846645 |
| 42 | H | -2.716842 | -1.279965 | 7.383793 |
| 43 | H | -1.083820 | -1.973767 | 7.326586 |
| 44 | H | -2.059989 | -1.883333 | 5.846645 |
| 45 | H | 0.992498 | -0.510582 | -2.505058 |
| 46 | H | 1.005320 | -2.596482 | -3.719295 |
| 47 | H | -0.992498 | -2.424735 | 0.810352 |
| 48 | H | -1.005320 | -4.519245 | -0.388972 |
| 49 | H | -0.103327 | -5.870715 | -4.709372 |
| 50 | H | 0.162882 | -4.137455 | -4.785202 |
| 51 | C | 1.960546 | -5.300850 | -4.373100 |
| 52 | H | 0.103327 | -7.013793 | -2.729502 |
| 53 | H | -0.162882 | -6.212834 | -1.190540 |
| 54 | C | -1.960546 | -6.437641 | -2.404121 |
| 55 | H | 2.235002 | -5.391519 | -5.429202 |
| 56 | H | 2.239799 | -6.229120 | -3.864019 |
| 57 | H | 2.535751 | -4.480149 | -3.933444 |
| 58 | H | -2.235002 | -7.397586 | -1.954592 |
| 59 | H | -2.239799 | -6.460899 | -3.462567 |
| 60 | H | -2.535751 | -5.646537 | -1.913200 |
| 61 | H | 0.992498 | 2.424735 | 0.810352 |
| 62 | H | 1.005320 | 4.519245 | -0.388972 |
| 63 | H | -0.992498 | 0.510582 | -2.505058 |
| 64 | H | -1.005320 | 2.596482 | -3.719295 |
| 65 | H | -0.103327 | 7.013793 | -2.729502 |
| 66 | H | 0.162882 | 6.212834 | -1.190540 |
| 67 | C | 1.960546 | 6.437641 | -2.404121 |
| 68 | H | 0.103327 | 5.870715 | -4.709372 |
| 69 | H | -0.162882 | 4.137455 | -4.785202 |
| 70 | C | -1.960546 | 5.300850 | -4.373100 |
| 71 | H | 2.235002 | 7.397586 | -1.954592 |
| 72 | H | 2.239799 | 6.460899 | -3.462567 |

| | | | | |
|---|---|---|---|---|
| 73 | H | 2.535751 | 5.646537 | -1.913200 |
| 74 | H | -2.235002 | 5.391519 | -5.429202 |
| 75 | H | -2.239799 | 6.229120 | -3.864019 |
| 76 | H | -2.535751 | 4.480149 | -3.933444 |

<u>**Synthesis**</u>

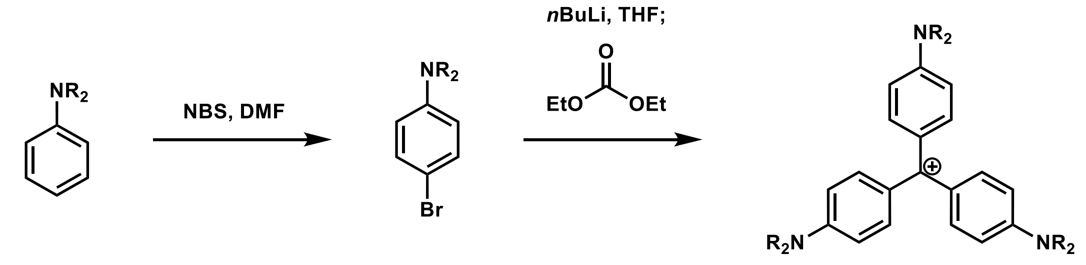


**4-Bromo-*N*,*N*-dipropylaniline (S1):**

To a stirred solution of *N*,*N*-dipropylaniline (100 mg, 0.564 mmol) in DMF (2.80 mL), NBS (105 mg, 0.592 mmol) was added at 0 °C, and the mixture was stirred at 0 °C for 1 h. Brine was added, and the aqueous layer was extracted with EtOAc. The combined organic layers were washed with brine, dried over $Na_2SO_4$, filtered, and concentrated to afford crude **S1** (131.4 mg), which was used for the next reaction without further purification.

**Tris(4-(dipropylamino)phenyl)methylium (5):**

To a stirred solution of crude **S1** (131.4 mg) in THF (3.42 mL), 1.6 M *n*BuLi in hexane (385 μL, 0.616 mmol) was added at -78 °C, and the mixture was stirred at -78 °C for 30 min. After that, diethylcarbonate (19.9 μL, 0.164 mmol) was added at -78 °C, and the mixture was stirred at r.t. for 1 day. TFA was added to quench the reaction, and 1 M NaOH solution was added to neutralize (pH 7). The aqueous layer was extracted with EtOAc. The combined organic layers were dried over $Na_2SO_4$, filtered, and concentrated to afford crude **5**, which was purified with silica gel column chromatography (2 to 18% MeOH/$CH_2Cl_2$) to afford **5** (as a TFA salt, 46.5 mg, 0.0711 mmol, y. 43% (2 steps)) as a purple solid.

$^1$H NMR ($CDCl_3$, 500 MHz) δ 7.27 (d, $J$ = 9.2 Hz, 6H), 6.73 (d, $J$ = 9.2 Hz, 6H), 3.40 (t, $J$ = 7.4 Hz, 12H), 1.72–1.65 (m, 12H), 0.96 (t, $J$ = 7.4 Hz, 18H); $^{13}$C NMR ($CDCl_3$, 126 MHz) δ 176.9, 154.0, 139.7, 126.1, 112.1, 53.1, 20.6, 11.2; $^{19}$F NMR ($CDCl_3$, 369 MHz) δ -74.2; ESI-LRMS *m/z* calcd for $[M]^+$ 540.4, Found: 540.2; ESI-HRMS: *m/z* calcd for $C_{37}H_{54}N_3$ $[M]^+$: 540.4312. Found: 540.4311.

**4-Bromo-*N*,*N*-diisopropylaniline (S2):**

To a stirred solution of *N*,*N*-diisopropylaniline (109.9 μL, 0.429 mmol) in DMF (2.8 mL), NBS (105 mg, 0.590 mmol) was added at 0 °C, and the mixture was stirred at 0 °C for 1 h. Brine was added, and the aqueous layer was extracted with EtOAc. The combined organic layers were washed with brine, dried over $Na_2SO_4$, filtered, and concentrated to afford crude **S2** (121.4 mg), which was used for the

next reaction without further purification.

**Tris(4-(diisopropylamino)phenyl)methylium (6):**

To a stirred solution of crude **S2** (121.4 mg) in THF (3.16 mL), 1.6 M *n*BuLi in hexane (356 μL, 0.570 mmol) was added at -78 °C, and the mixture was stirred at -78 °C for 30 min. After that, diethylcarbonate (18.4 μL, 0.152 mmol) was added at -78 °C, and the mixture was stirred at r.t. for 1 day. TFA was added to quench the reaction, and 1 M NaOH solution was added to neutralize (pH = 7). The aqueous layer was extracted with EtOAc. The combined organic layers were dried over $Na_2SO_4$, filtered, and concentrated to afford crude **6**, which was purified with silica gel column chromatography (2 to 18% MeOH/$CH_2Cl_2$) to afford **6** (as a TFA salt, 15.4 mg, 0.0247 mmol, y. 16% (2 steps)) as a purple solid.

$^1H$ NMR ($CDCl_3$, 500 MHz) δ 7.29 (d, *J* = 9.2 Hz, 6H), 6.94 (d, *J* = 9.2 Hz, 6H), 4.14 (sep, *J* = 6.9 Hz, 6H), 1.40 (d, J = 6.9 Hz, 36H); $^{13}C$ NMR ($CDCl_3$, 126 MHz) δ 176.5, 154.2, 139.2, 126.3, 114.8, 48.7, 20.8; $^{19}F$ NMR ($CDCl_3$, 369 MHz) δ -74.5; ESI-LRMS *m/z* calcd for $[M]^+$ 540.4, Found: 540.2; ESI-HRMS: *m/z* calcd for $C_{37}H_{54}N_3$ $[M]^+$: 540.4312. Found: 540.4314.

**4-Bromo-*N*,*N*-dibutylaniline (S3):**

To a stirred solution of *N*,*N*-dibutylaniline (100 mg, 0.487 mmol) in DMF (2.6 mL), NBS (91 mg, 0.511 mmol) was added at 0 °C, and the mixture was stirred at 0 °C for 1 h. Brine was added, and the aqueous layer was extracted with EtOAc. The combined organic layers were washed with brine, dried over $Na_2SO_4$, filtered, and concentrated to afford crude **S3** (128.6 mg), which was used for the next reaction without further purification.

**Tris(4-(dibutylamino)phenyl)methylium (7):**

To a stirred solution of crude **S3** (128.6 mg) in THF (3.01 mL), 1.6 M *n*BuLi in hexane (339 μL, 0.542 mmol) was added at -78 °C, and the mixture was stirred at -78 °C for 30 min. After that, diethylcarbonate (17.5 μL, 0.144 mmol) was added at -78 °C, and the mixture was stirred at r.t. for 1 day. TFA was added to quench the reaction, and 1 M NaOH solution was added to neutralize (pH = 7). The aqueous layer was extracted with EtOAc. The combined organic layers were dried over $Na_2SO_4$, filtered, and concentrated to afford crude **7**, which was purified with silica gel column chromatography (2 to 18% MeOH/$CH_2Cl_2$) to afford **7** (as a TFA salt, 14.4 mg, 0.0195 mmol, y. 14% (2 steps)) as a purple solid.

$^1H$ NMR ($CDCl_3$, 500 MHz) δ 7.30 (d, *J* = 8.0 Hz, 6H), 6.75 (d, *J* = 8.0 Hz, 6H), 3.44 (t, *J* = 7.4 Hz, 12H), 1.65 (m, 12H), 1.39 (m, 12H), 0.97 (t, *J* = 7.4 Hz, 18H); $^{13}C$ NMR ($CDCl_3$, 126 MHz) δ 176.9, 154.0, 139.8, 126.2, 112.1, 51.3, 29.5, 20.2, 13.9; $^{19}F$ NMR ($CDCl_3$, 369 MHz) δ -74.6; ESI-LRMS *m/z* calcd for $[M]^+$ 624.5, Found: 624.4; ESI-HRMS: *m/z* calcd for $C_{43}H_{66}N_3$ $[M]^+$: 624.5251. Found:

624.5249.

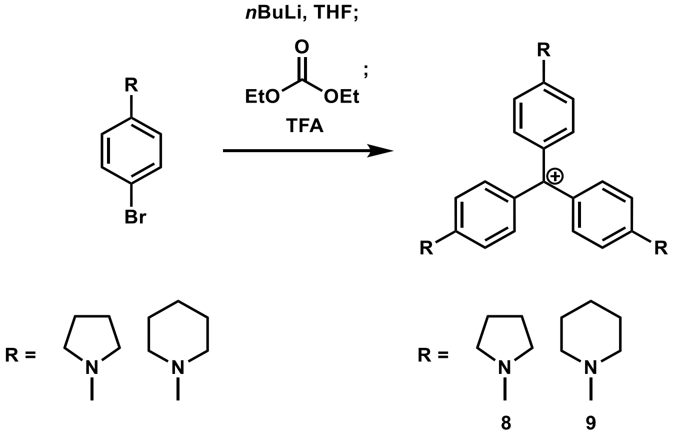


**Tris(4-(pyrrolidin-1-yl)phenyl)methylium (8):**

To a stirred solution of 1-(4-bromophenyl)pyrrolidine (113 mg, 0.500 mmol) in THF (3.33 mL), 1.6 M *n*BuLi in hexane (375 μL, 0.600 mmol) was added at -78 °C, and the mixture was stirred at -78 °C for 30 min. After that, diethylcarbonate (17.5 μL, 0.160 mmol) was added at -78 °C, and the mixture was stirred at r.t. for 1 day. TFA was added to quench the reaction, and 1 M NaOH solution was added to neutralize (pH = 7). The aqueous layer was extracted with EtOAc. The combined organic layers were dried over $Na_2SO_4$, filtered, and concentrated to afford crude **8**, which was purified with silica gel column chromatography (2 to 18% MeOH/$CH_2Cl_2$) to afford **8** (as a TFA salt, 23.0 mg, 0.0408 mmol, y. 26%) as a purple solid.

$^1$H NMR ($CDCl_3$, 500 MHz) δ 7.27 (d, $J$ = 8.6 Hz, 6H), 6.68 (d, $J$ = 8.6 Hz, 6H), 3.51 (t, $J$ = 6.9 Hz, 12H), 2.09 (t, $J$ = 6.9 Hz, 12H); $^{13}$C NMR ($CDCl_3$, 126 MHz) δ 177.9, 153.0, 139.7, 126.5, 112.8, 48.3, 25.3; $^{19}$F NMR ($CDCl_3$, 369 MHz) δ -74.5; ESI-LRMS *m/z* calcd for $[M]^+$450.3, Found: 450.8; ESI-HRMS: *m/z* calcd for $C_{31}H_{36}N_3$ $[M]^+$: 450.2904. Found: 450.2900.

**Tris(4-(piperidin-1-yl)phenyl)methylium (9):**

To a stirred solution of 1-(4-bromophenyl)piperidine (120 mg, 0.500 mmol) in THF (3.33 mL), 1.6 M *n*BuLi in hexane (375 μL, 0.600 mmol) was added at -78 °C, and the mixture was stirred at -78 °C for 30 min. After that, diethylcarbonate (17.5 μL, 0.160 mmol) was added at -78 °C, and the mixture was stirred at r.t. for 1 day. TFA was added to quench the reaction, and 1 M NaOH solution was added to neutralize (pH = 7). The aqueous layer was extracted with EtOAc. The combined organic layers were dried over $Na_2SO_4$, filtered, and concentrated to afford crude **9**, which was purified with silica gel column chromatography (2 to 18% MeOH/$CH_2Cl_2$) to afford **9** (as a TFA salt, 26.3 mg, 0.0434 mmol, y. 27%) as a purple solid.

$^{1}$H NMR ($CDCl_3$, 500 MHz) δ 7.28 (d, $J$ = 9.2 Hz, 6H), 6.92 (d, $J$ = 9.2 Hz, 6H), 3.59 (br, 12H), 1.72 (br, 12H); $^{13}$C NMR ($CDCl_3$, 126 MHz) δ 176.4, 155.3, 139.9, 126.9, 113.2, 48.4, 25.7, 24.2; $^{19}$F NMR ($CDCl_3$, 369 MHz) δ -74.6; ESI-LRMS *m/z* calcd for $[M]^+$ 492.3, Found: 492.2; ESI-HRMS: *m/z* calcd for $C_{34}H_{42}N_3$ $[M]^+$: 492.3373. Found: 492.3371.

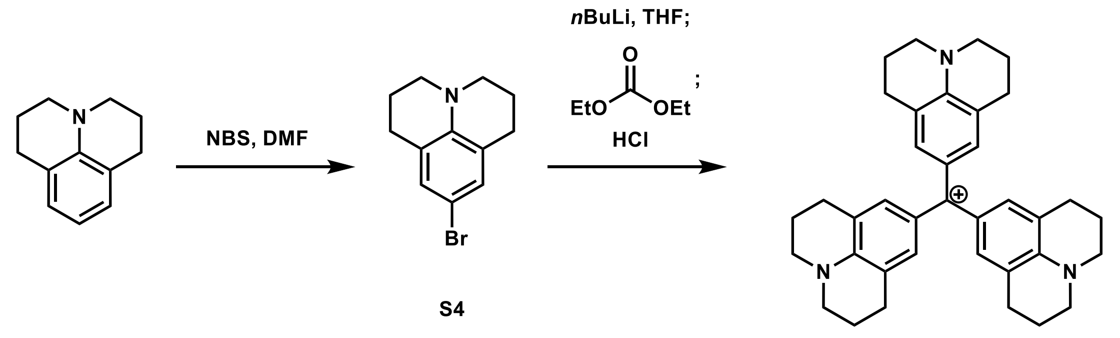


S4

10

**9-Bromo-julolidine (S4):**

To a stirred solution of julolidine (100 mg, 0.577 mmol) in DMF (2.9 mL), NBS (108 mg, 0.606 mmol) was added at 0 °C, and the mixture was stirred at 0 °C for 2 h. Brine was added, and the aqueous layer was extracted with EtOAc. The combined organic layers were washed with brine, dried over $Na_2SO_4$, filtered, and concentrated to afford crude **S4** (131.2 mg), which was used for the next reaction without further purification.

**Tris(2,3,6,7-tetrahydro-1*H*,5*H*-pyrido[3,2,1-*ij*]quinolin-9-yl)methylium (10):**

To a stirred solution of crude **S4** (131.2 mg) in THF (3.47 mL), 1.6 M *n*BuLi in hexane (390 µL, 0.624 mmol) was added at -78 °C, and the mixture was stirred at -78 °C for 30 min. After that, diethylcarbonate (20.2 µL, 0.167 mmol) was added at -78 °C, and the mixture was stirred at r.t. for 1 day. 1 M HCl was added to quench the reaction, and 1 M NaOH solution was added to neutralize (pH = 7). The aqueous layer was extracted with EtOAc. The combined organic layers were dried over $Na_2SO_4$, filtered, and concentrated to afford crude **10**, which was purified with preparative HPLC (eluent: 0.1% TFA aqueous solution/acetonitrile system) to afford **10** (as a TFA salt, 58.6 mg, 0.0913 mmol, y. 55% (2 steps)) as a purple solid.

$^{1}$H NMR ($CDCl_3$, 400 MHz) δ 6.80 (s, 6H), 3.41 (t, $J$ = 4.9 Hz, 12H), 2.70 (t, $J$ = 5.4 Hz, 12H), 1.98 (m, 12H); $^{13}$C NMR ($CDCl_3$, 100 MHz) δ 175.2, 149.3, 136.3, 126.2, 121.3, 50.5, 27.4, 20.8; $^{19}$F NMR ($CDCl_3$, 369 MHz) δ -74.2; ESI-LRMS *m/z* calcd for $[M]^+$ 528.3, Found: 528.2; ESI-HRMS: *m/z* calcd for $C_{37}H_{42}N_3$ $[M]^+$: 528.3373. Found: 528.3373.

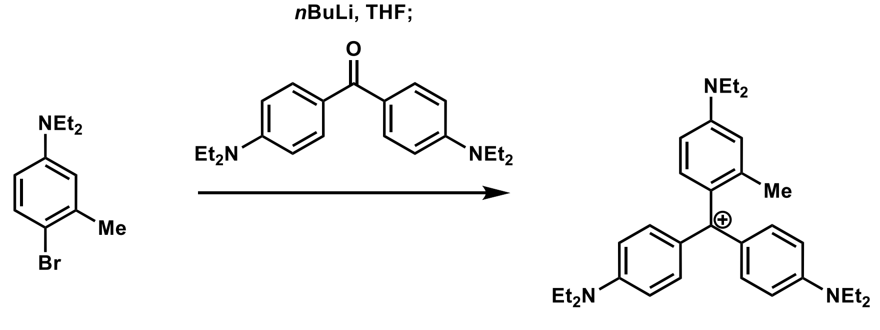


**11**

**(4-(Diethylamino)-2-methylphenyl)bis(4-(diethylamino)phenyl)methylium (11):**

To a stirred solution of 4-bromo-*N*,*N*-diethyl-3-methylaniline[12] (55 mg, 0.227 mmol) in THF (1.51 mL), 1.6 M *n*BuLi in hexane (156 µL, 0.250 mmol) was added at -78 °C, and the mixture was stirred at -78 °C for 30 min. After that, 4,4'-bis(diethylamino)benzophenone (73.6 mg, 0.227 mmol) was added at -78 °C, and the mixture was stirred at r.t. for 2 days. TFA (52 µL, 0.681 mmol) was added to quench the reaction and the mixture was evaporated. The residue was purified with silica gel column chromatography (2 to 19% MeOH/$CH_2Cl_2$) to afford **11** (as a TFA salt, 80.1 mg, 0.0913 mmol, y. 60%) as a purple solid.

$^1$H NMR ($CDCl_3$, 400 MHz) δ 7.24 (br, 4H), 6.95 (d, *J* = 8.5 Hz, 1H), 6.76 (d, *J* = 8.5 Hz, 4H), 6.55–6.52 (m, 2H), 3.54 (q, *J* = 7.2 Hz, 8H), 3.47 (q, *J* = 7.2 Hz, 4H), 1.84 (s, 3H), 1.28–1.23 (m, 18H); $^{13}$C NMR ($CDCl_3$, 100 MHz) δ 177.5, 154.1, 151.9, 144.8, 139.7, 139.6, 127.5, 127.2, 114.5, 112.3, 108.9, 45.4, 44.8, 22.3, 12.6, 12.5; $^{19}$F NMR ($CDCl_3$, 369 MHz) δ -74.8; ESI-LRMS *m/z* calcd for $[M]^+$ 470.4, Found: 470.2; ESI-HRMS: *m*/*z* calcd for $C_{32}H_{44}N_3$ $[M]^+$: 470.3530. Found: 470.3530.

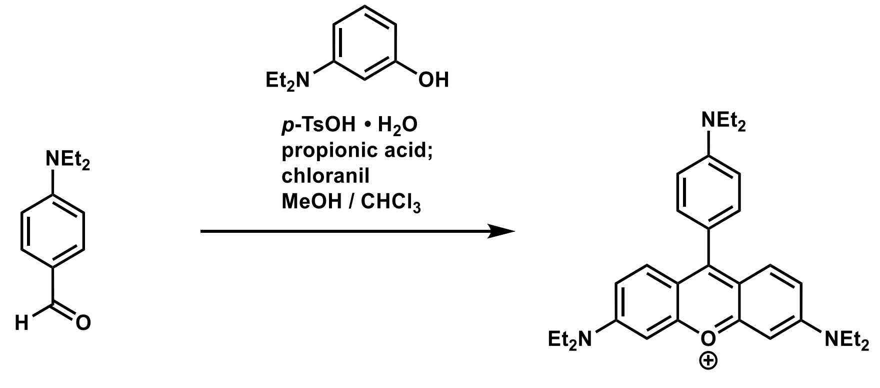


**12**

---

[12] Novakova, V., Miletin, M., Kopecky, K., Zimcik, P. (2011). Red-Emitting Dyes with Photophysical and Photochemical Properties Controlled by pH. Chem. Eur. J. *17*, 14273-14282. 10.1002/chem.201101123.

**3,6-Bis(diethylamino)-9-(4-(diethylamino)phenyl)xanthylium (12):**

To a stirred solution of 4-(diethylamino)benzaldehyde (100 mg) in propionic acid (5.6 mL), 3-diethylaminophenol (186 mg, 1.13 mmol), and *p*-TsOH·$H_2O$ (10.7 mg, 0.0564 mmol) were added, and the mixture was stirred at 65 °C for 27 h. After cooling to r.t., 3 M NaOAc was added to basify. The aqueous layer was extracted with $CHCl_3$. The combined organic layers were dried over $Na_2SO_4$, filtered, and concentrated. To the residue, MeOH (11 mL), $CHCl_3$ (11 mL), and chloranil (69 mg, 0.282 mmol) were added, and the mixture was stirred at r.t. for 1 h. After that, the mixture was evaporated. The residue was purified with silica gel column chromatography (9% MeOH/$CH_2Cl_2$) to afford crude **12** (115.9 mg). To this crude **12**, 0.1% TFA aqueous solution was added, and it was neutralized with 1 M NaOH solution (pH = 7). The aqueous layer was extracted with $CH_2Cl_2$. The combined organic layers were dried over $Na_2SO_4$, filtered, and concentrated. The residue was purified with silica gel column chromatography (amino silica, 2 to 10% MeOH/$CH_2Cl_2$) to afford **12** (as a TFA salt, 89.2 mg, 0.153 mmol, y. 27%) as a red solid.

$^1$H NMR ($CDCl_3$, 400 MHz) δ 7.83 (d, $J$ = 9.4 Hz, 2H), 7.21 (d, $J$ = 9.0 Hz, 2H), 6.80 (dd, $J$ = 2.2 Hz, 9.4 Hz, 2H), 6.74 (d, $J$ = 9.0 Hz, 2H), 6.65 (d, $J$ = 2.2 Hz, 2H), 3.51 (q, $J$ = 7.2 Hz, 8H), 3.39 (q, $J$ = 7.2 Hz, 4H), 1.22 (t, $J$ = 7.2 Hz, 12H), 1.17 (t, $J$ = 7.2 Hz, 6H); $^{13}$C NMR ($CDCl_3$, 100 MHz) δ 158.3, 157.7, 154.6, 149.5, 132.5, 132.2, 117.2, 113.1, 112.5, 110.8, 96.0, 45.6, 44.3, 12.31, 12.27; $^{19}$F NMR ($CDCl_3$, 369 MHz) δ -74.3; ESI-LRMS *m/z* calcd for $[M]^+$ 470.3, Found: 470.1; ESI-HRMS: *m/z* calcd for $C_{31}H_{40}N_3O$ $[M]^+$: 470.3166. Found: 470.3166.

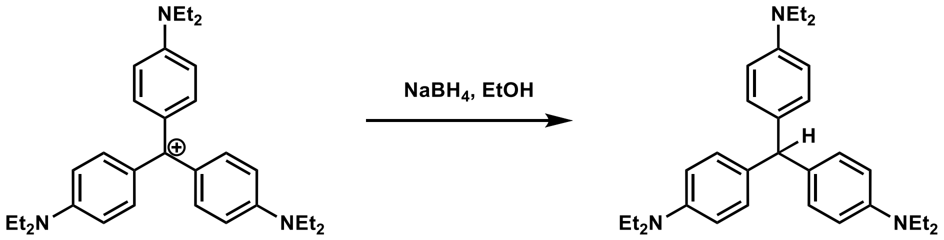


EV (3) LEV (2)

**4,4',4''-Methanetriyltris(*N*,*N*-diethylaniline) (LEV, 2):**

To a stirred solution of **EV** (**3**) (as a hemi(zinc chloride) salt (commercially available), 200 mg, 0.357 mmol) in EtOH (1 mL), $NaBH_4$ (121 mg, 3.21 mmol) was added at 0 °C, and the mixture was stirred at 0 °C for 2 h. Water was added at 0 °C, and the aqueous layer was extracted with $CH_2Cl_2$. The combined organic layers were dried over $Na_2SO_4$, purified with short-pad silica gel, filtered, and concentrated to afford **LEV** (**2**) (164 mg, quant.) as a yellowish-white solid or oil.

$^1$H NMR ($CDCl_3$, 400 MHz) δ 7.00 (d, $J$ = 8.5 Hz, 6H), 6.61 (d, $J$ = 8.5 Hz, 6H), 5.25 (s, 1H), 3.32 (q,

*J* = 6.7 Hz, 12H), 1.15 (t, *J* = 6.7 Hz, 18H); $^{13}C$ NMR ($CDCl_3$, 100 MHz) δ 145.9, 132.6, 130.0, 111.6, 53.9, 44.3, 12.6; ESI-LRMS *m/z* calcd for $[M+H]^+$ 458.4, Found: 458.3; ESI-HRMS: *m*/*z* calcd for $C_{31}H_{43}N_3$ $[M+H]^+$: 458.3530. Found: 458.3519.

NMR charts

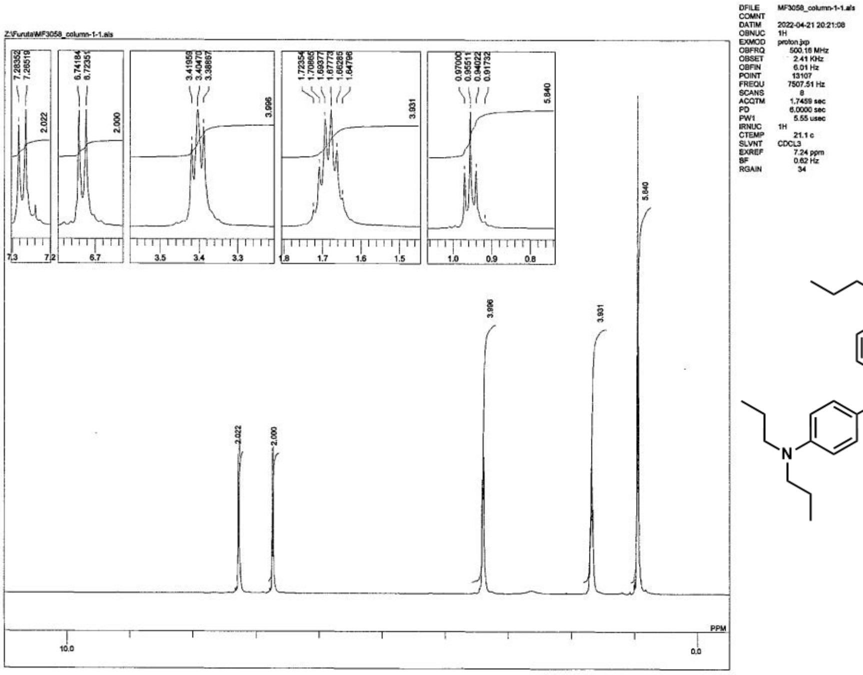


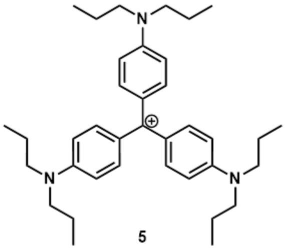

5

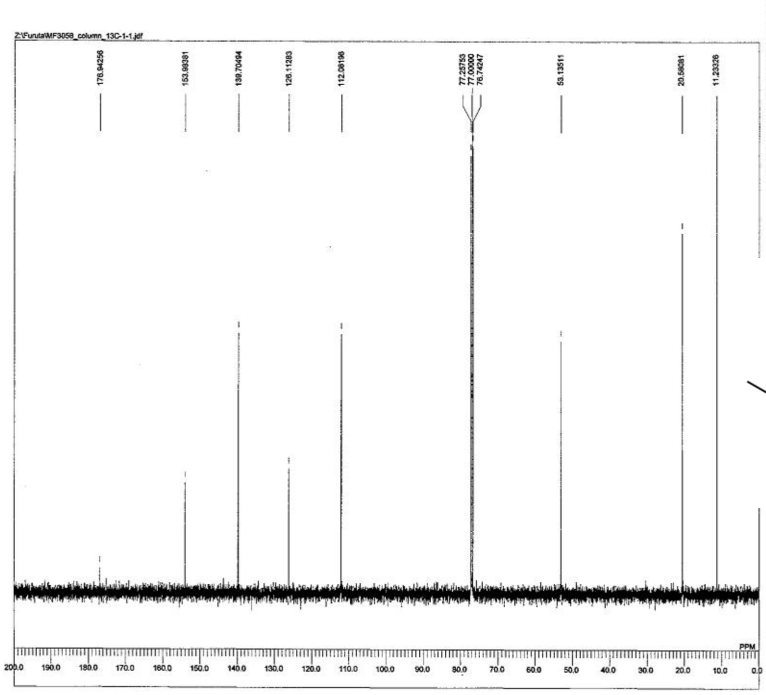


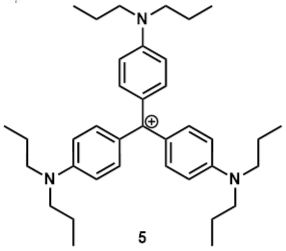

5

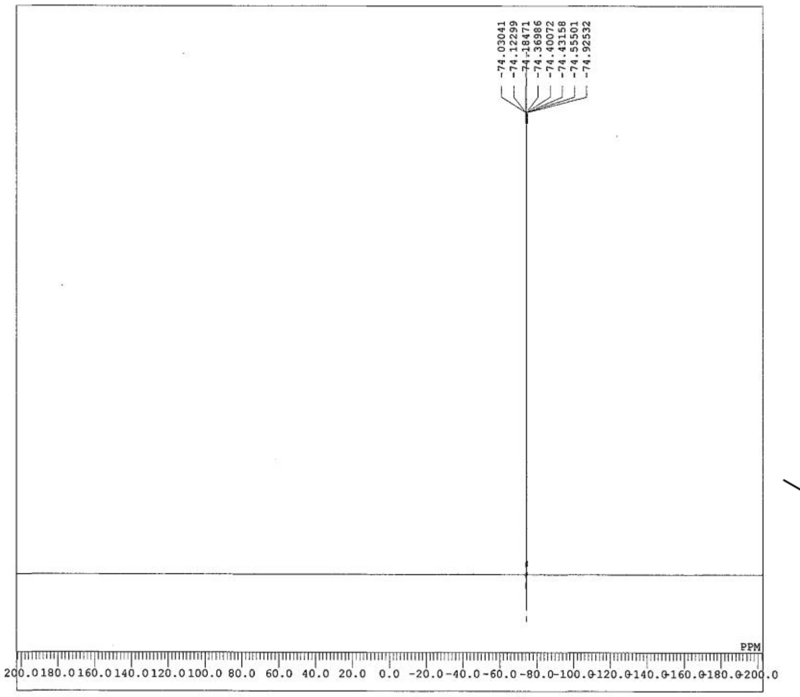


```
DFILE   MF3058_column_19F-1-4.jdf
COMNT
DATIM   2022-04-21 22:46:12
OBNUC   19F
EXMOD   proton.jxp
OBFRQ        368.64 MHz
OBSET          7.63 KHz
OBFIN          2.85 Hz
POINT         16400
FREQU     186567.17 Hz
SCANS             8
ACQTM        0.0878 sec
PD           6.0000 sec
PW1            4.10 usec
IRNUC   19F
CTEMP          20.6 c
SLVNT   CDCL3
EXREF          0.00 ppm
BF             1.62 Hz
RGAIN            50
```

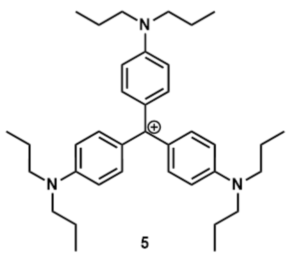

**5**

```
DFILE   MF3059_column-1-1.jdf
COMNT
DATIM   2022-04-21 20:30:44
OBNUC   1H
EXMOD   proton.jxp
OBFRQ      500.16 MHz
OBSET        2.41 KHz
OBFIN        6.01 Hz
POINT       16384
FREQU     9384.38 Hz
SCANS           8
ACQTM      1.7459 sec
PD         6.0000 sec
PW1          5.55 usec
IRNUC   1H
CTEMP        21.2 c
SLVNT   CDCL3
EXREF        7.24 ppm
BF           0.62 Hz
RGAIN          36
```

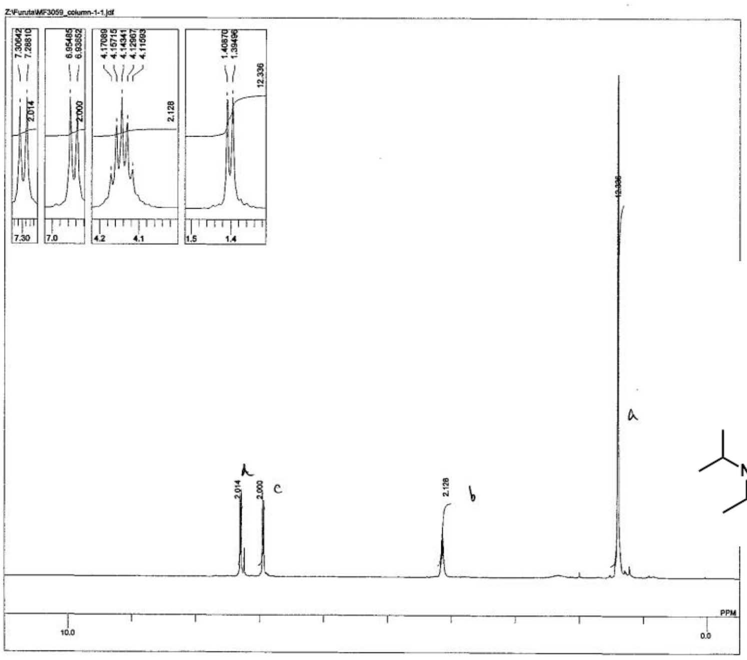


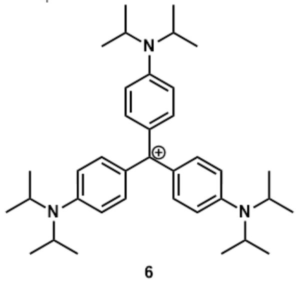

**6**

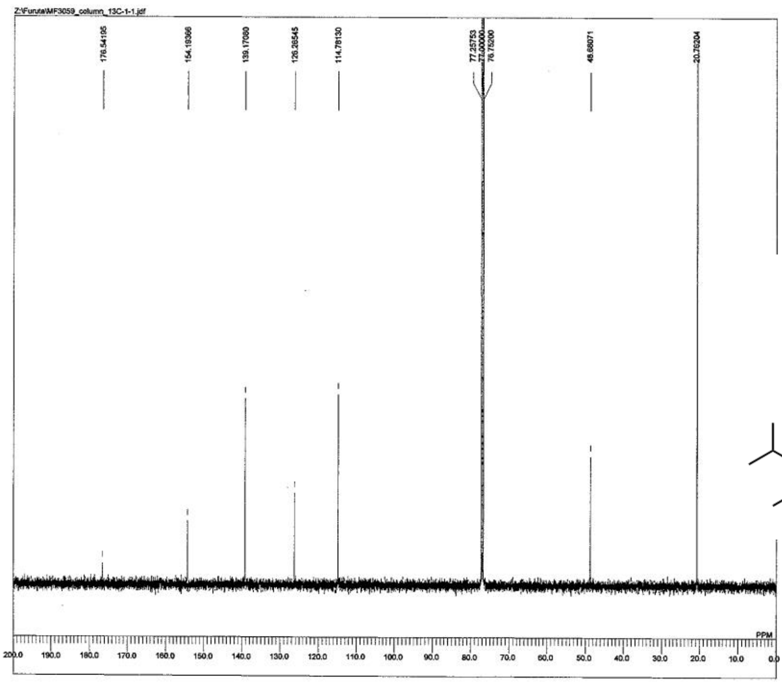
Z:\Furuta\MF3059_column_13C-1-1.jdf
176.54195
154.19366
139.17080
126.26545
114.78130
77.25753
77.00000
76.75200
48.68071
20.76204
DFILE MF3059_column_13C-1-1.jdf
COMNT
DATIM 2022-04-21 22:16:19
OBNUC 13C
EXMOD carbon.jxp
OBFRQ 125.77 MHz
OBSET 7.87 KHz
OBFIN 4.21 Hz
POINT 32767
FREQU 39308.18 Hz
SCANS 288
ACQTM 0.8336 sec
PD 2.0000 sec
PW1 3.40 usec
IRNUC 1H
CTEMP 21.3 c
SLVNT CDCL3
EXREF 77.00 ppm
BF 0.62 Hz
RGAIN 60
PPM
200.0 190.0 180.0 170.0 160.0 150.0 140.0 130.0 120.0 110.0 100.0 90.0 80.0 70.0 60.0 50.0 40.0 30.0 20.0 10.0 0.0

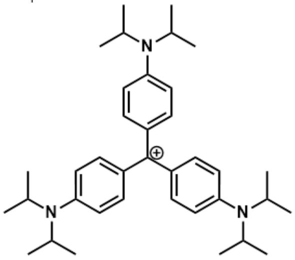
N
N
N
⊕

**6**

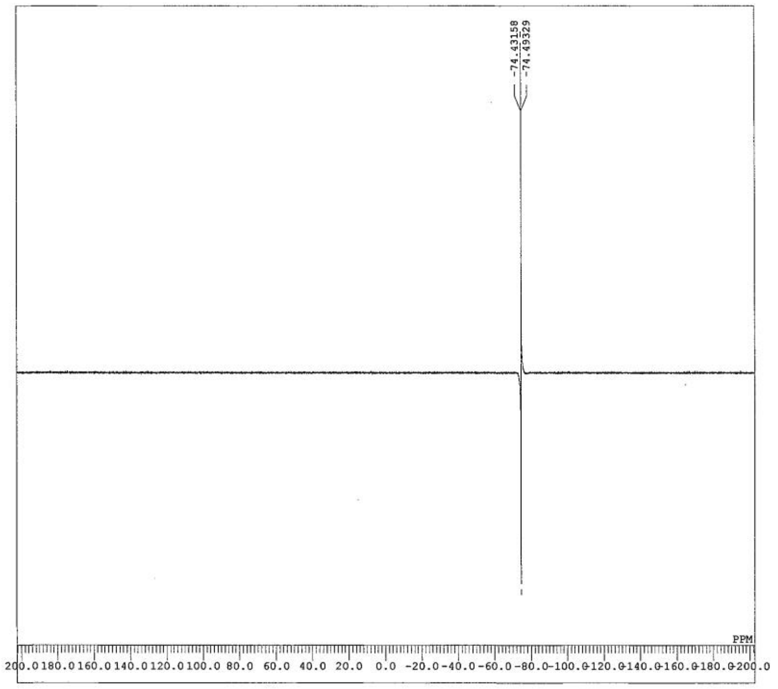
-74.43158
-74.49329
DFILE MF3059_column_19F-1-3.jdf
COMNT
DATIM 2022-04-21 22:50:25
OBNUC 19F
EXMOD proton.jxp
OBFRQ 368.64 MHz
OBSET 7.63 KHz
OBFIN 2.85 Hz
POINT 16400
FREQU 186567.17 Hz
SCANS 8
ACQTM 0.0878 sec
PD 6.0000 sec
PW1 4.10 usec
IRNUC 19F
CTEMP 20.6 c
SLVNT CDCL3
EXREF 0.00 ppm
BF 1.62 Hz
RGAIN 50
PPM
200.0 180.0 160.0 140.0 120.0 100.0 80.0 60.0 40.0 20.0 0.0 -20.0 -40.0 -60.0 -80.0 -100.0 -120.0 -140.0 -160.0 -180.0 -200.0

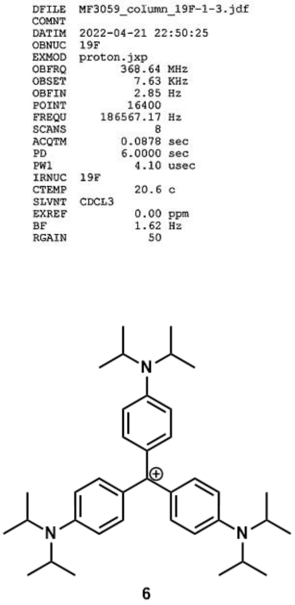
N
N
N
⊕

**6**

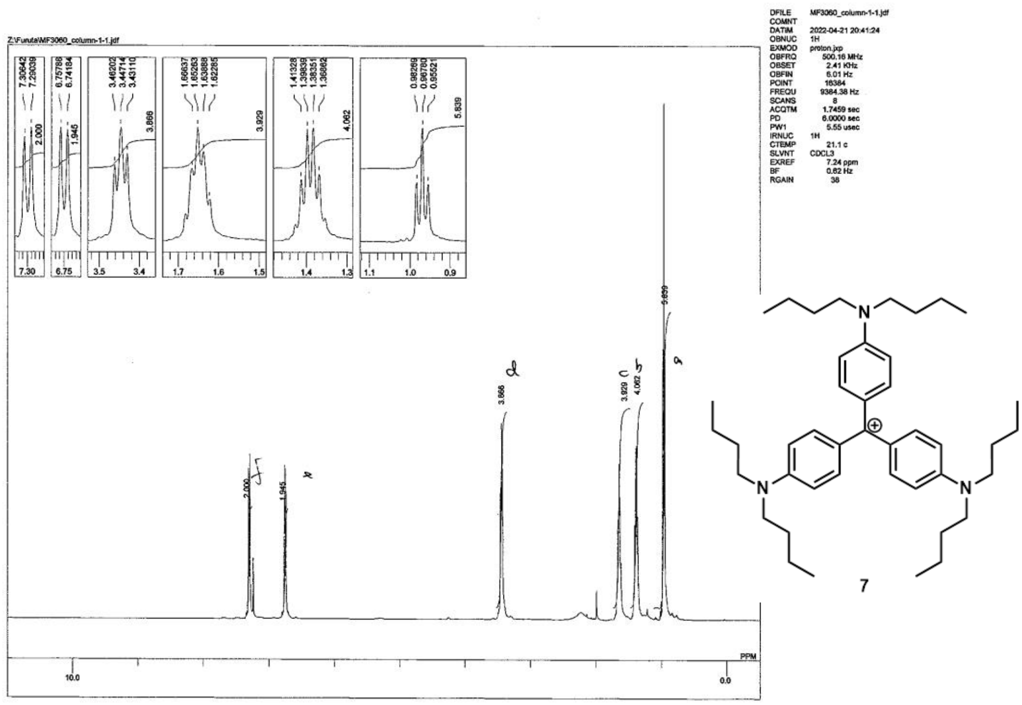
Z:\Furuta\MF3060_column-1-1.jdf
7.30642
7.29039
6.75788
6.74184
3.46202
3.44714
3.43110
1.66637
1.65263
1.63888
1.62285
1.41328
1.39839
1.38351
1.36862
0.98269
0.96780
0.95521
2.000
1.945
3.866
3.929
4.062
5.839
7.30
6.75
3.5
3.4
1.7
1.6
1.5
1.4
1.3
1.1
1.0
0.9
PPM
10.0
0.0
DFILE MF3060_column-1-1.jdf
COMNT
DATIM 2022-04-21 20:41:24
OBNUC 1H
EXMOD proton.jxp
OBFRQ 500.16 MHz
OBSET 2.41 KHz
OBFIN 6.01 Hz
POINT 16384
FREQU 9384.38 Hz
SCANS 8
ACQTM 1.7459 sec
PD 6.0000 sec
PW1 5.55 usec
IRNUC 1H
CTEMP 21.1 c
SLVNT CDCL3
EXREF 7.24 ppm
BF 0.62 Hz
RGAIN 38
N
7

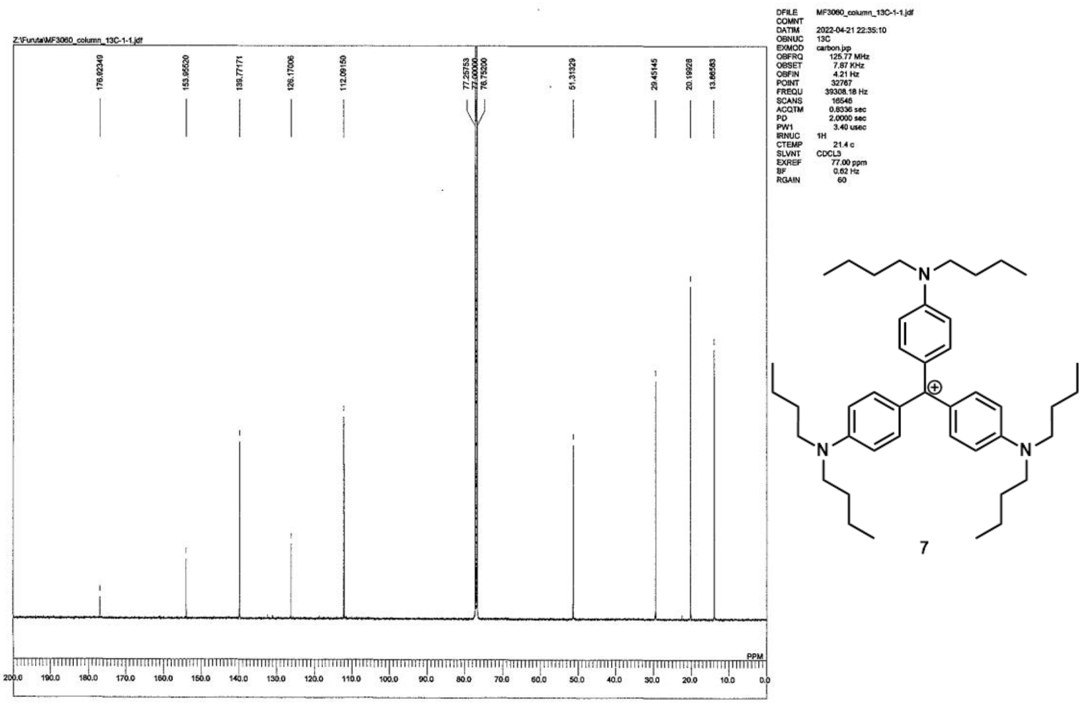
Z:\Furuta\MF3060_column_13C-1-1.jdf
176.92349
153.95520
139.77171
126.17006
112.09150
77.25753
77.00000
76.75200
51.31329
29.45145
20.19928
13.86583
PPM
200.0
190.0
180.0
170.0
160.0
150.0
140.0
130.0
120.0
110.0
100.0
90.0
80.0
70.0
60.0
50.0
40.0
30.0
20.0
10.0
0.0
DFILE MF3060_column_13C-1-1.jdf
COMNT
DATIM 2022-04-21 22:35:10
OBNUC 13C
EXMOD carbon.jxp
OBFRQ 125.77 MHz
OBSET 7.87 KHz
OBFIN 4.21 Hz
POINT 32767
FREQU 39308.18 Hz
SCANS 16546
ACQTM 0.8336 sec
PD 2.0000 sec
PW1 3.40 usec
IRNUC 1H
CTEMP 21.4 c
SLVNT CDCL3
EXREF 77.00 ppm
BF 0.62 Hz
RGAIN 60
N
7

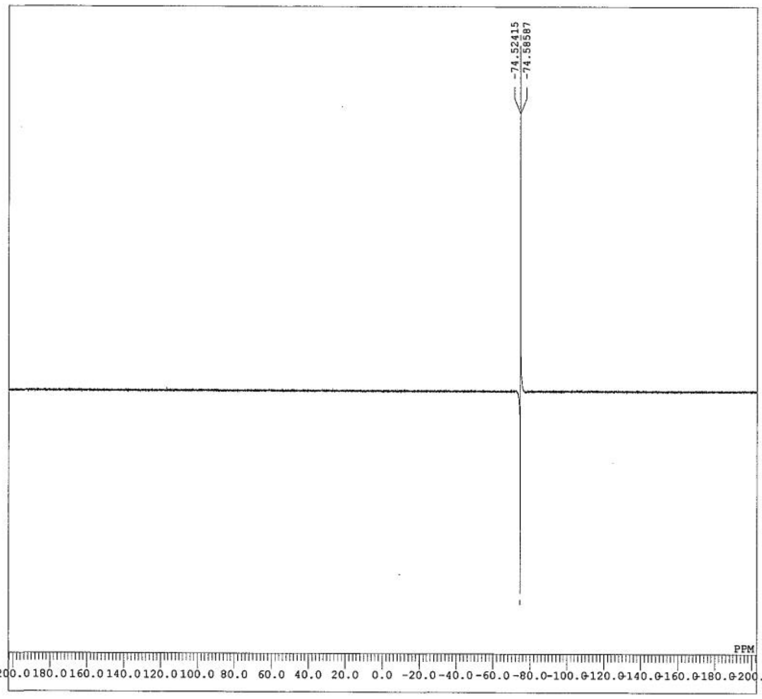
-74.52415
-74.58587
DFILE MF3060_column_19F-1-3.jdf
COMNT
DATIM 2022-04-21 22:42:10
OBNUC 19F
EXMOD proton.jxp
OBFRQ 368.64 MHz
OBSET 7.63 KHz
OBFIN 2.85 Hz
POINT 16400
FREQU 186567.17 Hz
SCANS 8
ACQTM 0.0878 sec
PD 6.0000 sec
PW1 4.10 usec
IRNUC 19F
CTEMP 20.7 c
SLVNT CDCL3
EXREF 0.00 ppm
BF 1.62 Hz
RGAIN 50
PPM
200.0 180.0 160.0 140.0 120.0 100.0 80.0 60.0 40.0 20.0 0.0 -20.0 -40.0 -60.0 -80.0 -100.0 -120.0 -140.0 -160.0 -180.0 -200.0

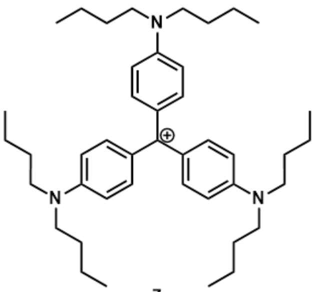

7

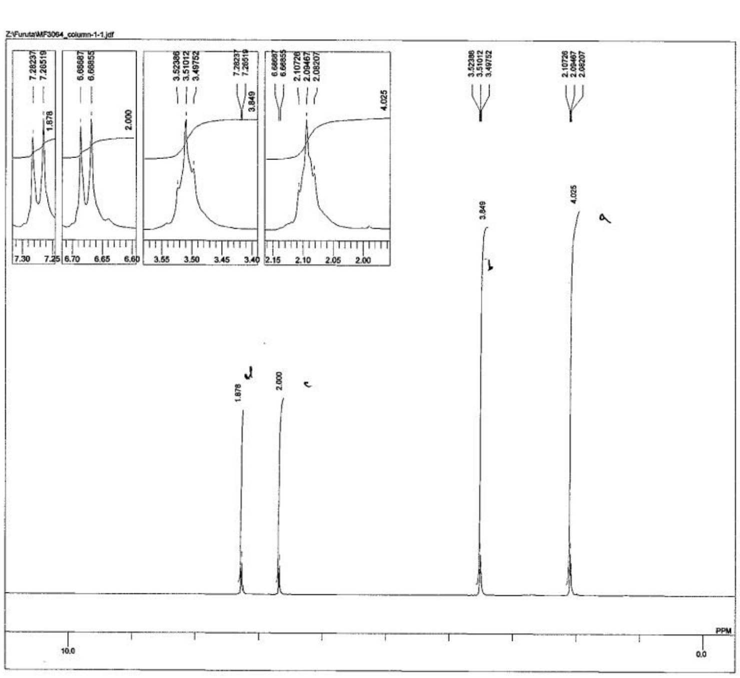
Z:\Furuta\MF3064_column-1-1.jdf
DFILE MF3064_column-1-1.jdf
COMNT
DATIM 2022-04-21 21:29:03
OBNUC 1H
EXMOD proton.jxp
OBFRQ 500.16 MHz
OBSET 2.41 KHz
OBFIN 6.01 Hz
POINT 16384
FREQU 9384.38 Hz
SCANS 8
ACQTM 1.7459 sec
PD 6.0000 sec
PW1 5.55 usec
IRNUC 1H
CTEMP 20.9 c
SLVNT CDCL3
EXREF 7.24 ppm
BF 0.62 Hz
RGAIN 36
7.28237
7.26519
6.66687
6.64955
3.52386
3.51012
3.49752
2.10726
2.09467
2.08207
1.878
2.000
3.849
4.025
PPM
10.0
0.0

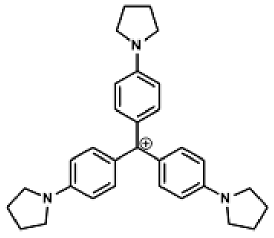

8

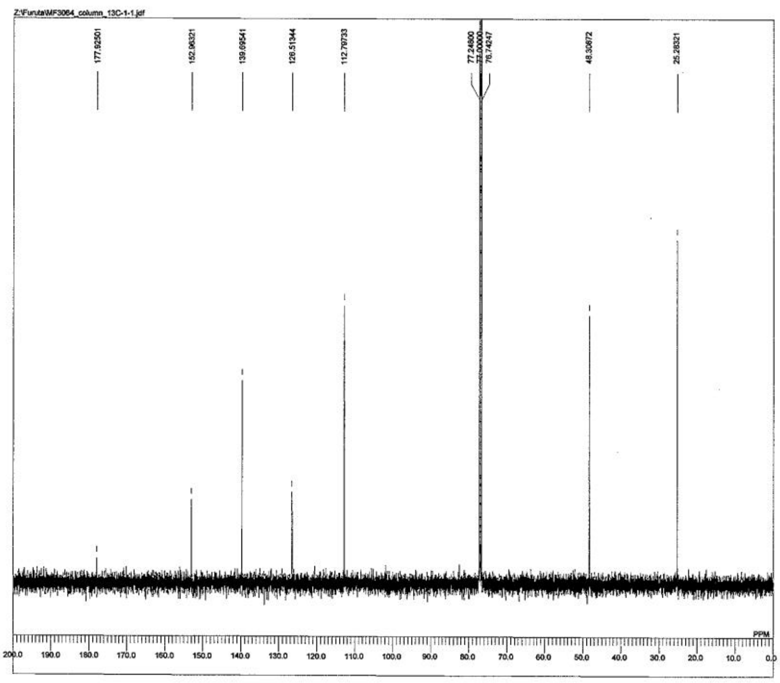


DFILE MF3064_column_13C-1-1.jdf
COMNT
DATIM 2022-04-21 21:30:53
OBNUC 13C
EXMOD carbon.jxp
OBFRQ 125.77 MHz
OBSET 7.87 KHz
OBFIN 4.21 Hz
POINT 32767
FREQU 39308.18 Hz
SCANS 110
ACQTM 0.8336 sec
PD 2.0000 sec
PW1 3.40 usec
IRNUC 1H
CTEMP 21.3 c
SLVNT CDCL3
EXREF 77.00 ppm
BF 0.62 Hz
RGAIN 60

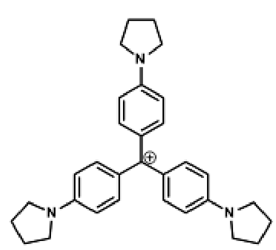

8

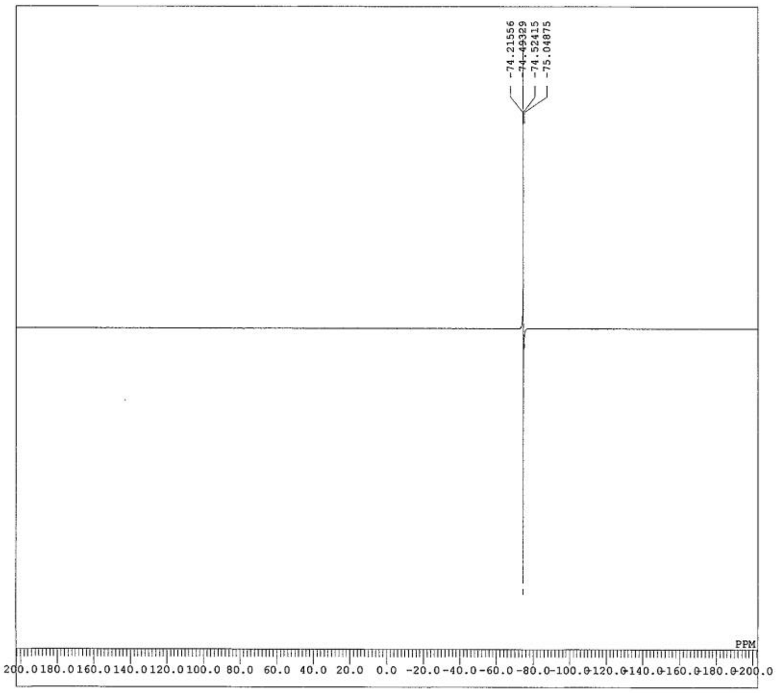


DFILE MF3064_column_19F-1-3.jdf
COMNT
DATIM 2022-04-21 22:57:13
OBNUC 19F
EXMOD proton.jxp
OBFRQ 368.64 MHz
OBSET 7.63 KHz
OBFIN 2.85 Hz
POINT 16400
FREQU 186567.17 Hz
SCANS 8
ACQTM 0.0878 sec
PD 6.0000 sec
PW1 4.10 usec
IRNUC 19F
CTEMP 20.5 c
SLVNT CDCL3
EXREF 0.00 ppm
BF 1.62 Hz
RGAIN 50

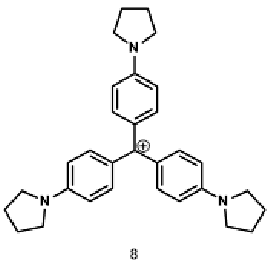

8

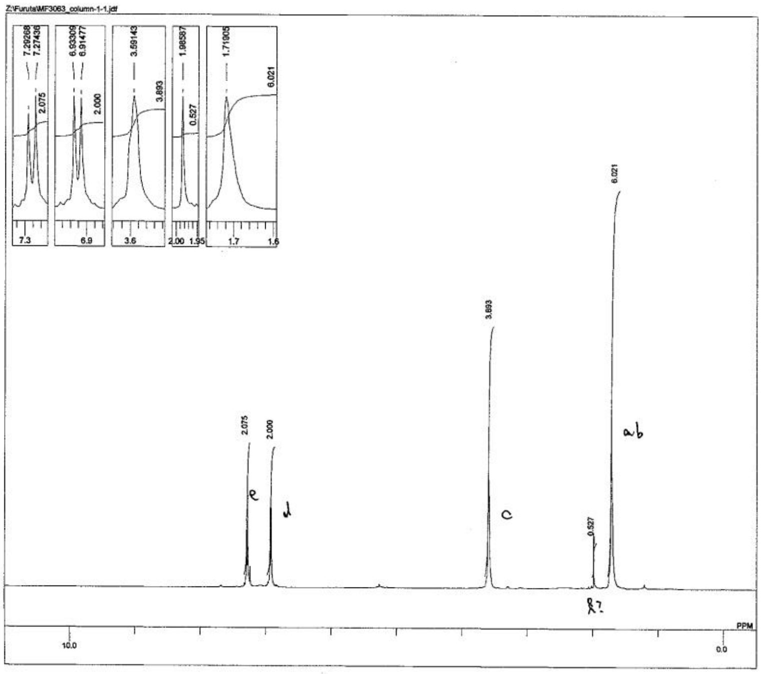
Z:\Furuta\MF3063_column-1-1.jdf
7.29268
7.27436
6.93309
6.91477
3.59143
1.98587
1.71905
2.075
2.000
3.893
0.527
6.021
e
d
c
ab
PPM
10.0
0.0
DFILE MF3063_column-1-1.jdf
COMNT
DATIM 2022-04-21 21:17:21
OBNUC 1H
EXMOD proton.jxp
OBFRQ 500.16 MHz
OBSET 2.41 KHz
OBFIN 6.01 Hz
POINT 16384
FREQU 9384.38 Hz
SCANS 8
ACQTM 1.7459 sec
PD 6.0000 sec
PW1 5.55 usec
IRNUC 1H
CTEMP 20.9 c
SLVNT CDCL3
EXREF 7.24 ppm
BF 0.62 Hz
RGAIN 36

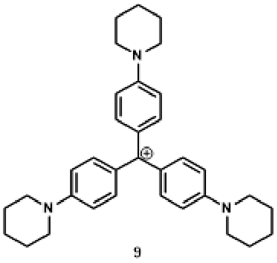

9

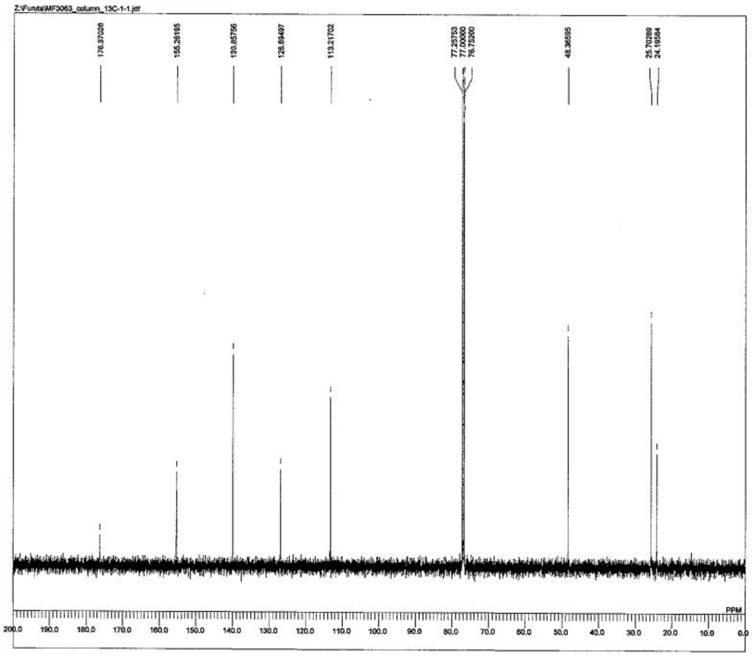
Z:\Furuta\MF3063_column_13C-1-1.jdf
176.37026
155.26195
139.65756
126.69497
113.21702
77.25753
77.00000
76.75200
48.36595
25.70269
24.19584
PPM
200.0
190.0
180.0
170.0
160.0
150.0
140.0
130.0
120.0
110.0
100.0
90.0
80.0
70.0
60.0
50.0
40.0
30.0
20.0
10.0
0.0
DFILE MF3063_column_13C-1-1.jdf
COMNT
DATIM 2022-04-21 21:19:12
OBNUC 13C
EXMOD carbon.jxp
OBFRQ 125.77 MHz
OBSET 7.87 KHz
OBFIN 4.21 Hz
POINT 32767
FREQU 39308.18 Hz
SCANS 107
ACQTM 0.8336 sec
PD 2.0000 sec
PW1 3.40 usec
IRNUC 1H
CTEMP 21.2 c
SLVNT CDCL3
EXREF 77.00 ppm
BF 0.62 Hz
RGAIN 60

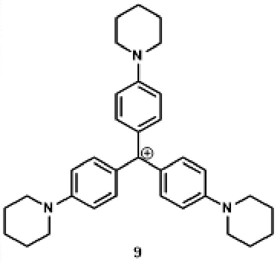

9

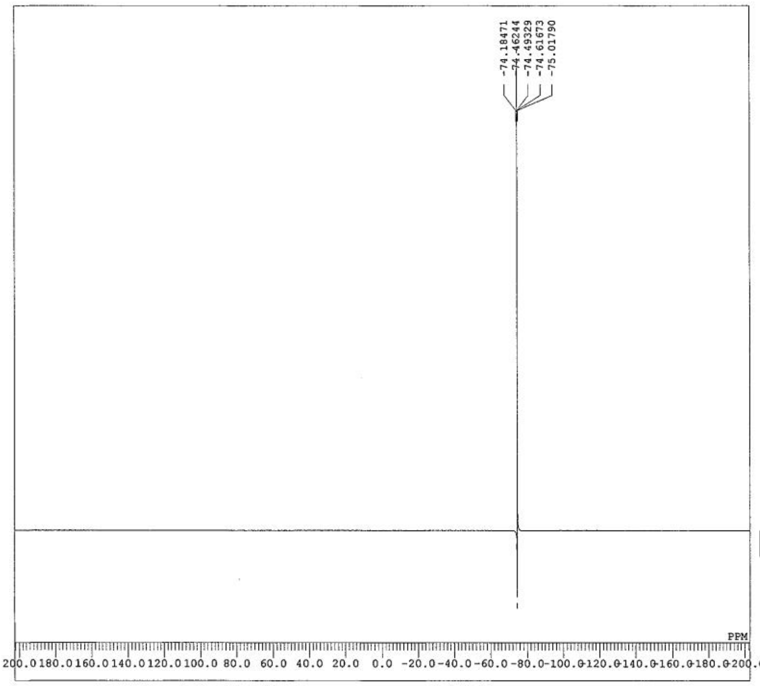


```
DFILE  MF3063_column_19F-1-3.jdf
COMNT
DATIM  2022-04-21 22:53:41
OBNUC  19F
EXMOD  proton.jxp
OBFRQ       368.64 MHz
OBSET         7.63 KHz
OBFIN         2.85 Hz
POINT        16400
FREQU    186567.17 Hz
SCANS            8
ACQTM       0.0878 sec
PD          6.0000 sec
PW1           4.10 usec
IRNUC  19F
CTEMP         20.6 c
SLVNT  CDCL3
EXREF         0.00 ppm
BF            1.62 Hz
RGAIN           50
```

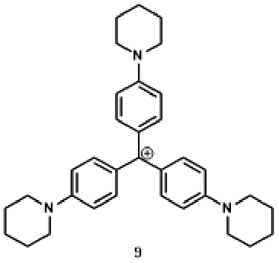

9

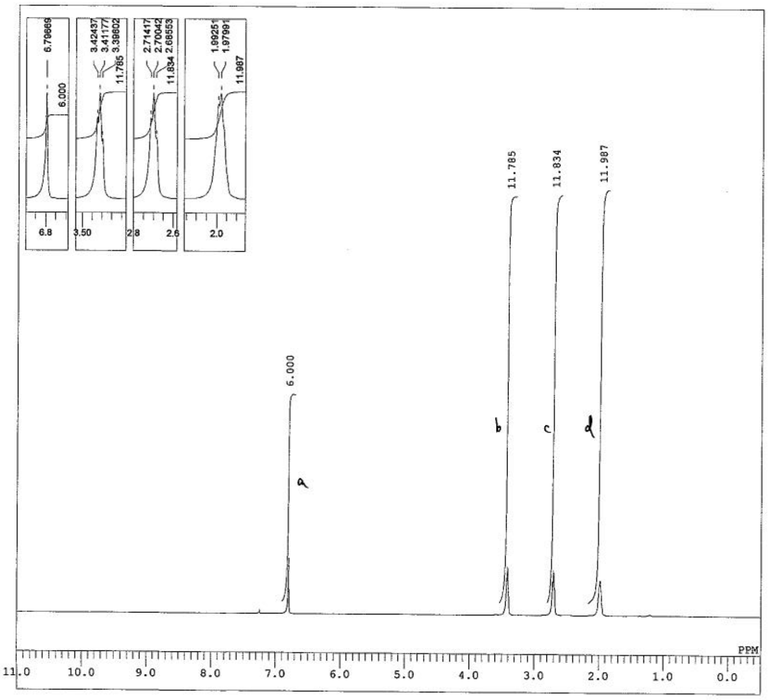


```
DFILE  MF3047_prep-1-1.jdf
COMNT
DATIM  2022-04-06 22:05:31
OBNUC  1H
EXMOD  proton.jxp
OBFRQ       391.78 MHz
OBSET         8.51 KHz
OBFIN         3.34 Hz
POINT        16384
FREQU      7352.94 Hz
SCANS            8
ACQTM       2.2282 sec
PD          5.0000 sec
PW1           5.17 usec
IRNUC  1H
CTEMP         20.6 c
SLVNT  CDCL3
EXREF         7.24 ppm
BF            1.20 Hz
RGAIN           32
```

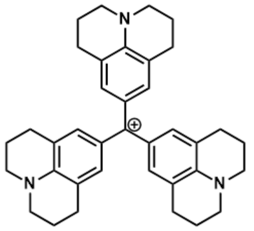

**10**

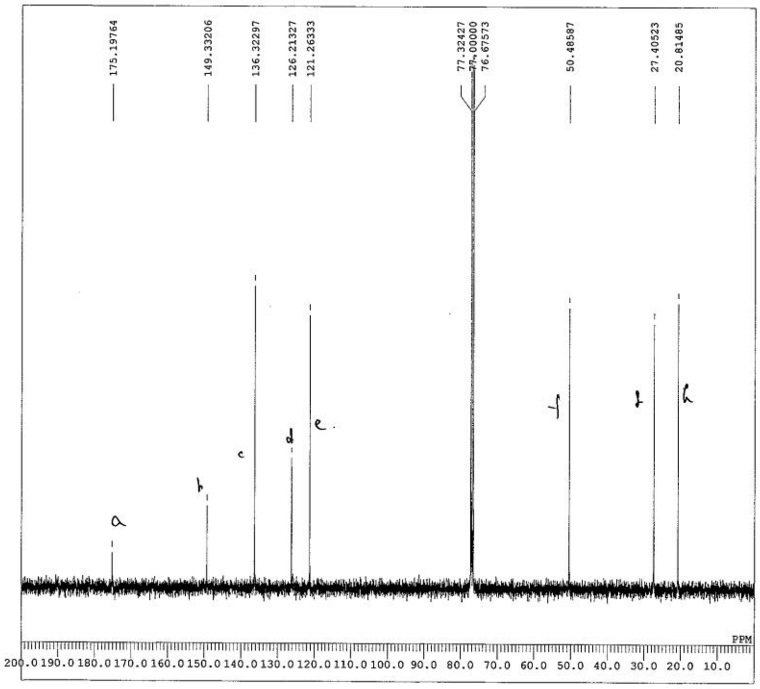


DFILE MF3047_prep_13C-1-1.jdf
COMNT
DATIM 2022-04-06 22:13:30
OBNUC 13C
EXMOD carbon.jxp
OBFRQ 98.52 MHz
OBSET 4.64 KHz
OBFIN 8.74 Hz
POINT 32767
FREQU 30788.18 Hz
SCANS 153
ACQTM 1.0643 sec
PD 2.0000 sec
PW1 3.12 usec
IRNUC 1H
CTEMP 20.7 c
SLVNT CDCL3
EXREF 77.00 ppm
BF 1.20 Hz
RGAIN 60

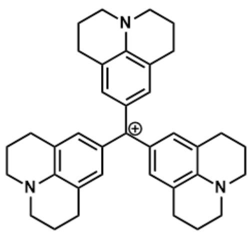

**10**

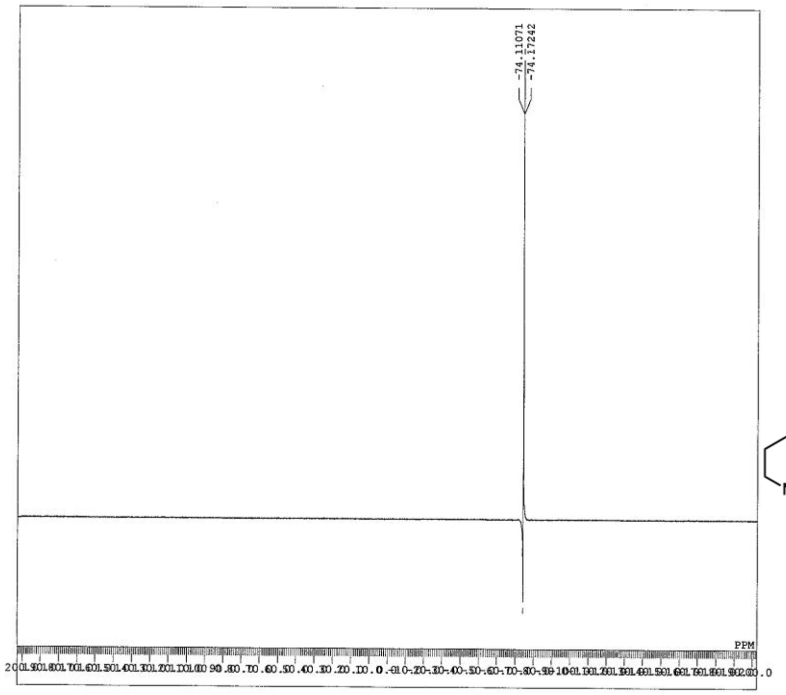


DFILE MF3047_prep_19F-1-3.jdf
COMNT
DATIM 2022-04-06 22:10:27
OBNUC 19F
EXMOD proton.jxp
OBFRQ 368.64 MHz
OBSET 7.63 KHz
OBFIN 2.85 Hz
POINT 16400
FREQU 186567.17 Hz
SCANS 8
ACQTM 0.0878 sec
PD 6.0000 sec
PW1 4.10 usec
IRNUC 19F
CTEMP 20.6 c
SLVNT CDCL3
EXREF 202.45 ppm
BF 1.20 Hz
RGAIN 50

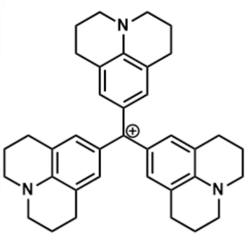

**10**

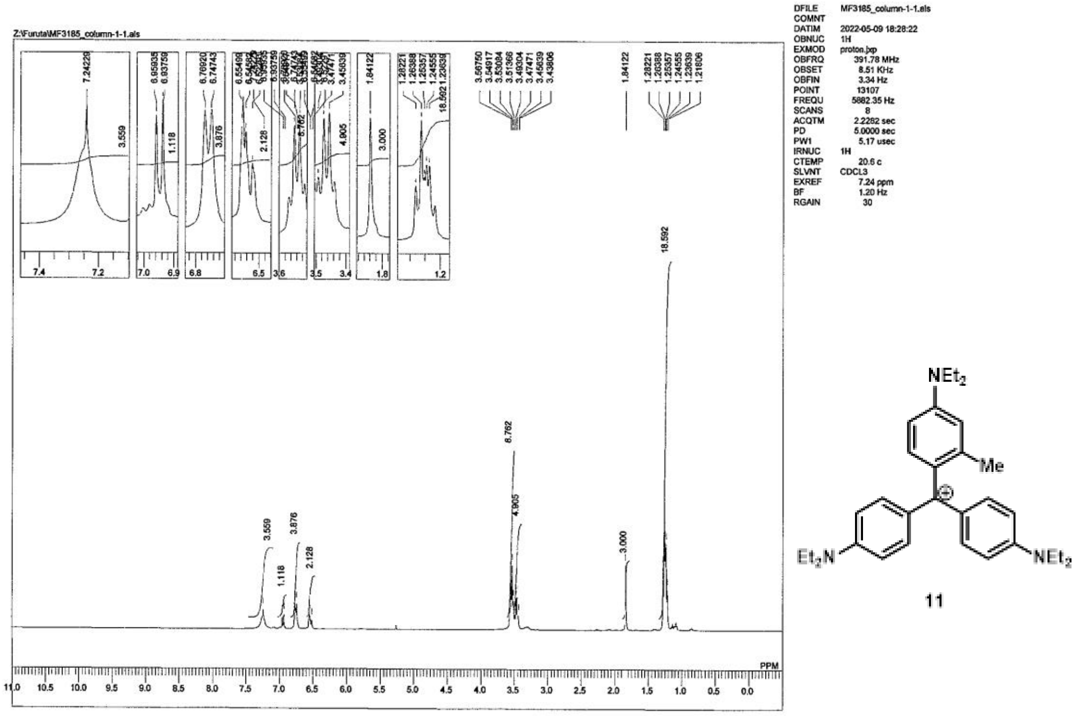

11

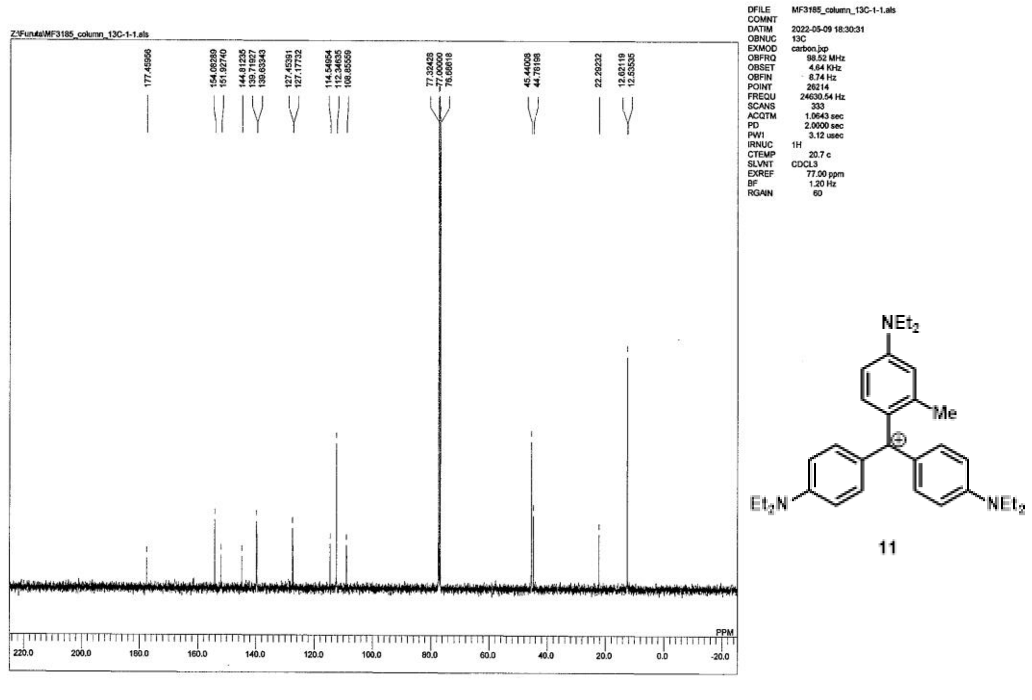

11

Z:\Furuta\MF3185_column_19F-1-3.als

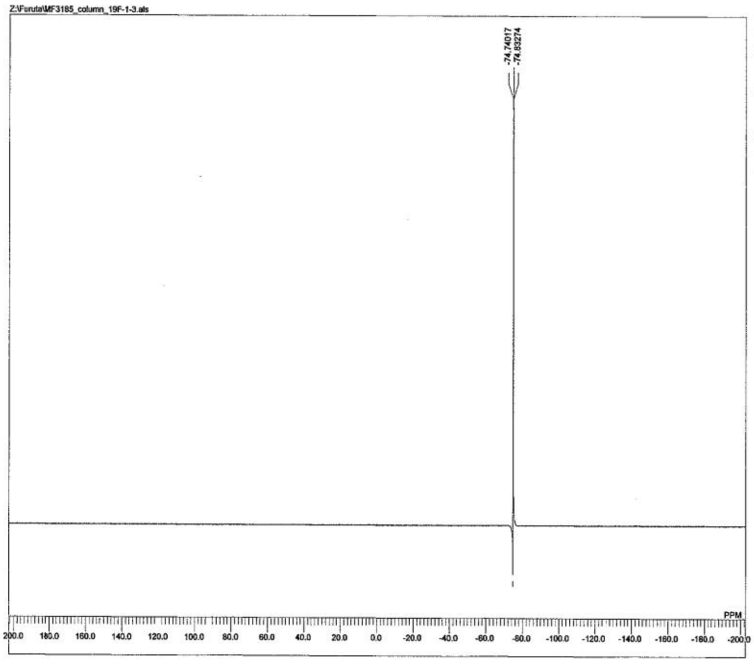


DFILE MF3185_column_19F-1-3.als
COMNT
DATIM 2022-05-09 18:49:37
OBNUC 19F
EXMOD proton.jxp
OBFRQ 368.64 MHz
OBSET 7.63 KHz
OBFIN 2.85 Hz
POINT 13120
FREQU 149253.73 Hz
SCANS 6
ACQTM 0.0878 sec
PD 6.0000 sec
PW1 4.10 usec
IRNUC 19F
CTEMP 20.6 c
SLVNT CDCL3
EXREF 0.00 ppm
BF 1.20 Hz
RGAIN 48

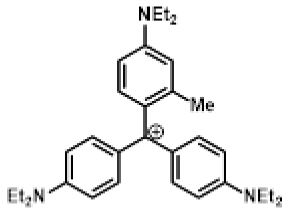


**11**

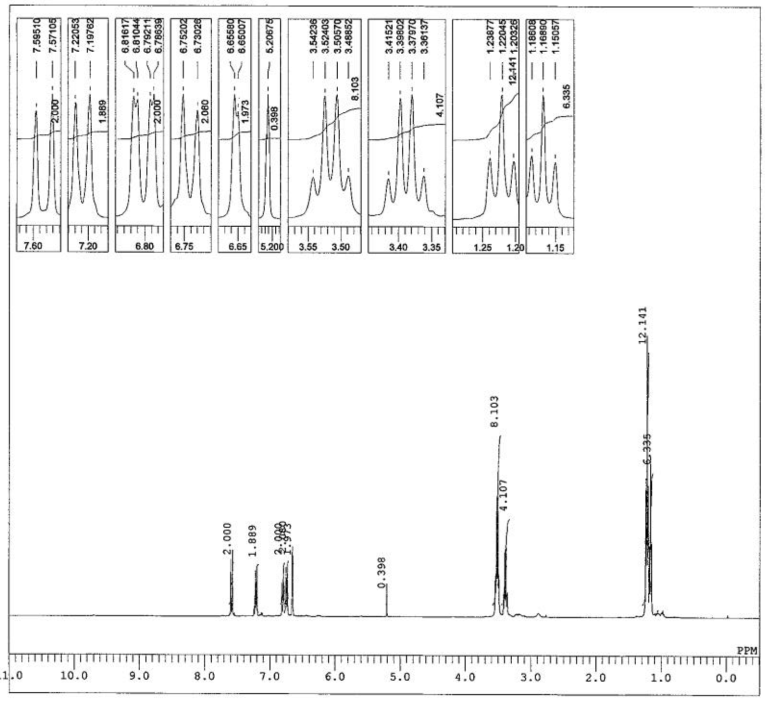


DFILE MF3221_column-1-1.jdf
COMNT
DATIM 2022-05-19 19:59:55
OBNUC 1H
EXMOD proton.jxp
OBFRQ 391.78 MHz
OBSET 8.51 KHz
OBFIN 3.34 Hz
POINT 16384
FREQU 7352.94 Hz
SCANS 8
ACQTM 2.2282 sec
PD 5.0000 sec
PW1 5.17 usec
IRNUC 1H
CTEMP 20.6 c
SLVNT CDCL3
EXREF 7.24 ppm
BF 0.20 Hz
RGAIN 22

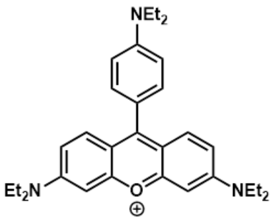


**12**

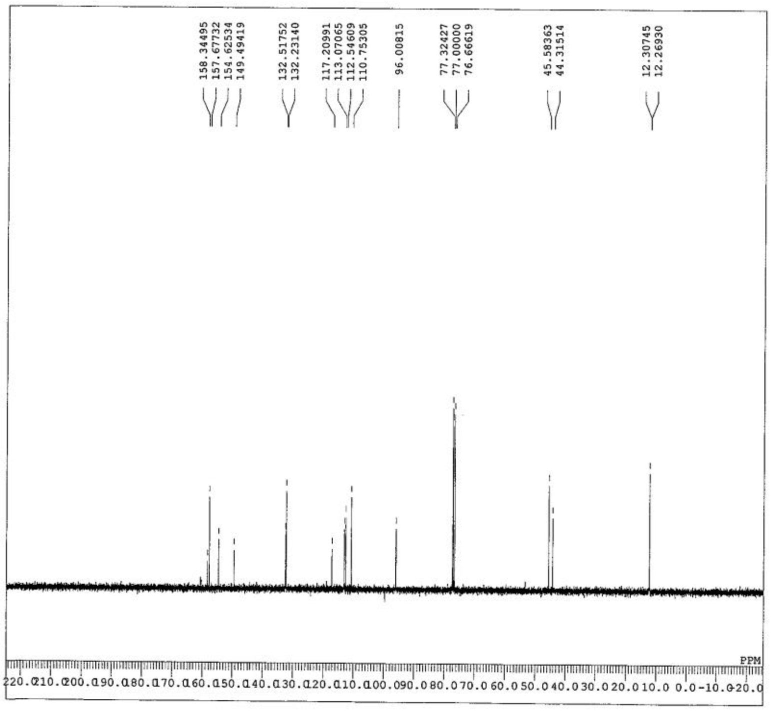


DFILE MF3221_column_13C-1-1.jdf
COMNT
DATIM 2022-05-19 20:01:30
OBNUC 13C
EXMOD carbon.jxp
OBFRQ 98.52 MHz
OBSET 4.64 KHz
OBFIN 8.74 Hz
POINT 32767
FREQU 30788.18 Hz
SCANS 101
ACQTM 1.0643 sec
PD 2.0000 sec
PW1 3.12 usec
IRNUC 1H
CTEMP 20.8 c
SLVNT CDCL3
EXREF 77.00 ppm
BF 0.20 Hz
RGAIN 60

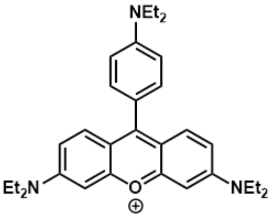


12

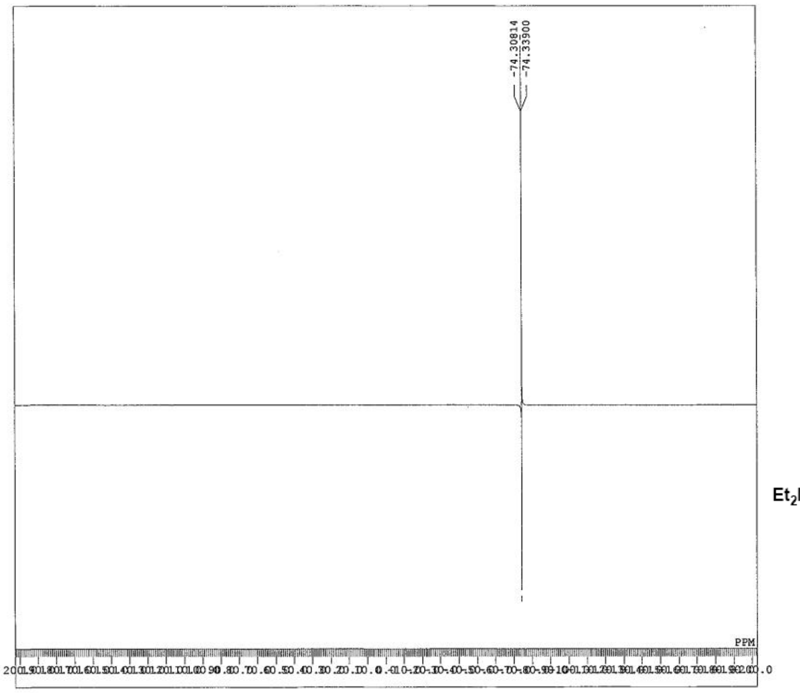


DFILE MF3221_column_19F-1-3.jdf
COMNT
DATIM 2022-05-19 20:08:28
OBNUC 19F
EXMOD proton.jxp
OBFRQ 368.64 MHz
OBSET 7.63 KHz
OBFIN 2.85 Hz
POINT 16400
FREQU 186567.17 Hz
SCANS 8
ACQTM 0.0878 sec
PD 6.0000 sec
PW1 4.10 usec
IRNUC 19F
CTEMP 20.6 c
SLVNT CDCL3
EXREF 0.00 ppm
BF 0.20 Hz
RGAIN 48

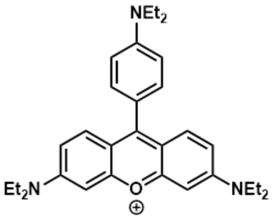


12

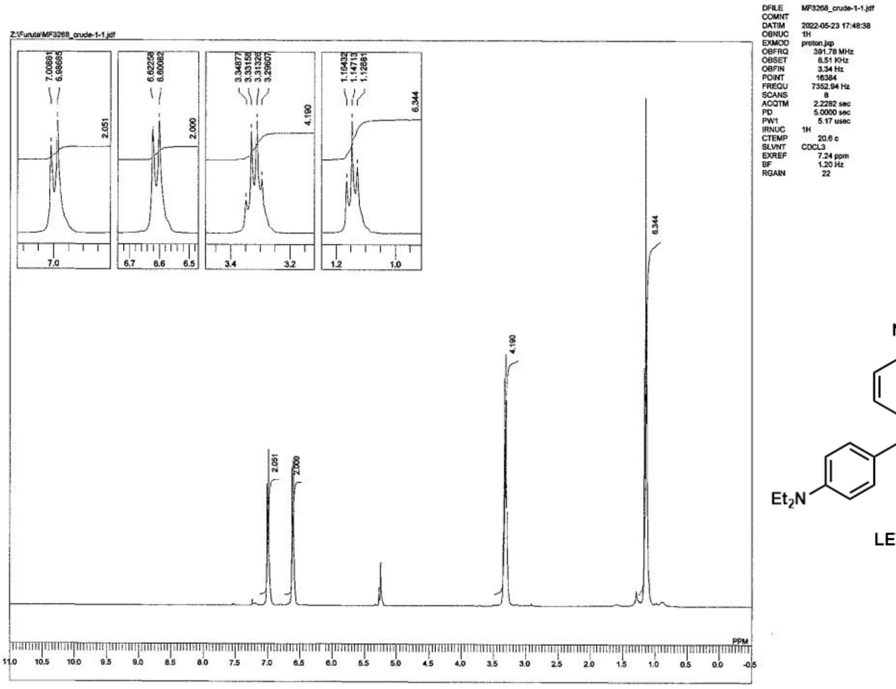
Z:\Furuta\MF3268_crude-1-1.jdf
DFILE MF3268_crude-1-1.jdf
COMNT
DATIM 2022-05-23 17:48:38
OBNUC 1H
EXMOD proton.jxp
OBFRQ 391.78 MHz
OBSET 8.51 KHz
OBFIN 3.34 Hz
POINT 16384
FREQU 7352.94 Hz
SCANS 8
ACQTM 2.2282 sec
PD 5.0000 sec
PW1 5.17 usec
IRNUC 1H
CTEMP 20.6 c
SLVNT CDCL3
EXREF 7.24 ppm
BF 1.20 Hz
RGAIN 22
7.00681
6.98685
6.62256
6.60082
3.34877
3.33158
3.31326
3.29607
1.16432
1.14713
1.12881
2.051
2.000
4.190
6.344
PPM

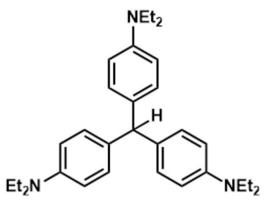
NEt2
H
Et2N
NEt2

LEV (2)

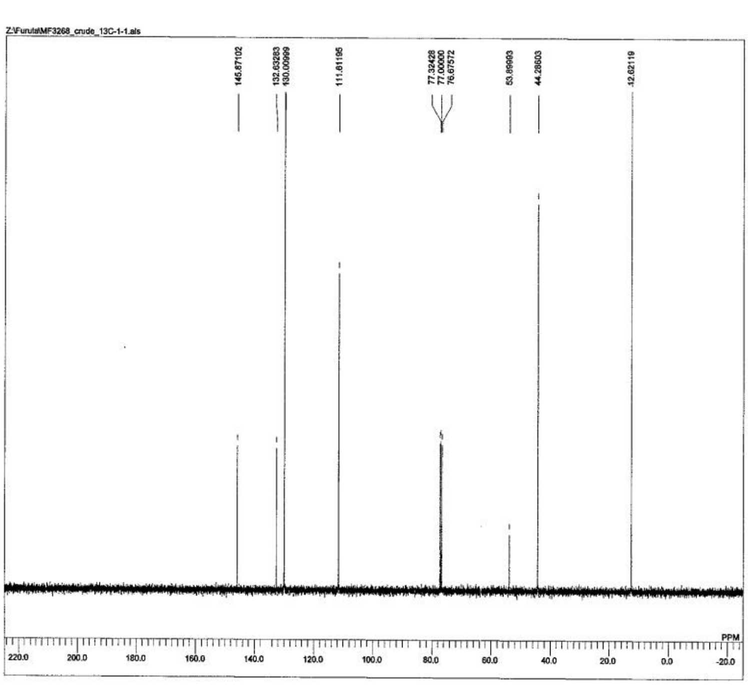
Z:\Furuta\MF3268_crude_13C-1-1.als
DFILE MF3268_crude_13C-1-1.als
COMNT
DATIM 2022-05-23 17:50:30
OBNUC 13C
EXMOD carbon.jxp
OBFRQ 98.52 MHz
OBSET 4.64 KHz
OBFIN 8.74 Hz
POINT 26214
FREQU 24630.54 Hz
SCANS 57
ACQTM 1.0643 sec
PD 2.0000 sec
PW1 3.12 usec
IRNUC 1H
CTEMP 20.9 c
SLVNT CDCL3
EXREF 77.00 ppm
BF 0.20 Hz
RGAIN 60
145.87102
132.63283
130.00999
111.61195
77.32428
77.00000
76.67572
53.89993
44.28603
12.62119
PPM

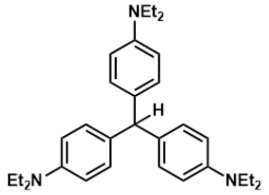
NEt2
H
Et2N
NEt2

LEV (2)
